# Folate and global health umbrella review series, part 2: syntheses on cancers

**DOI:** 10.7189/jogh.16.04102

**Published:** 2026-02-20

**Authors:** Samantha Yoo, Azita Montazeri, Derrick Bennett, Yacong Bo, Peizhan Chen, Susan Duthie, Natalie Jensen, Atipatsa Kaminga, Jun-Shi Lai, Xue Li, Amanda J MacFarlane, Homero Martinez, Helene McNulty, Franco Momoli, Peter Mossey, Patrick Mullie, Ron Munger, Rajendra Prasad Parajuli, Monique Potvin Kent, Michele Rubini, Marjanne Senekal, Lindsey Sikora, Alain Stintzi, Evropi Theodoratou, Hui Wang, Chittaranjan Yajnik, Ann Yaktine, Julian Little

**Affiliations:** 1 School of Epidemiology and Public Health, Faculty of Medicine, University of Ottawa, Canada; 2 Division of Medical Sciences, Division of Population Health, University of Oxford, UK; 3 School of Public Health, Xinxiang Medical University, China; 4 Clinical Research Centre, Ruijin Hospital, Shanghai Jiao Tong University School of Medicine, Shanghai, China; 5 School of Pharmacy and Life Sciences, Robert Gordon University, Scotland, UK; 6 Department of Mathematics and Statistics, Faculty of Science, Technology and Innovation, Mzuzu University, Malawi; 7 Singapore Institute for Clinical Sciences, Agency for Science, Technology and Research, Singapore; 8 Centre for Population Health Sciences, University of Edinburgh, UK; 9 Nutrition Research Division, Health Canada, Canada; 10 Research and Development Unit, Nutrition International, Canada; 11 Nutrition Innovation Centre for Food and Health, School of Biomedical Sciences, Ulster University, Coleraine, Northern Ireland, UK; 12 School of Dentistry, University of Dundee, Scotland, UK; 13 International Prevention Research Institute, Lyon, France; 14 Department of Nutrition, Dietetics, and Food Sciences, College of Agriculture and Applied Sciences, Utah State University, USA; 15 Herbert Wertheim School of Public Health and Human Longevity Science, University of California San Diego, USA; 16 Department of Neuroscience and Rehabilitation, University of Ferrara, Italy; 17 Department of Human Biology, Faculty of Health Sciences, University of Cape Town, South Africa; 18 School of Pharmacy, Faculty of Medicine, University of Ottawa, Canada; 19 Centre for Global Health, Usher Institute, College of Medicine and Veterinary Medicine, University of Edinburgh, UK; 20 School of Public Health, Faculty of Medicine, Shanghai Jiao Tong University, China; 21 Diabetes Unit, KEM Hospital Research Centre, India; 22 Food and Nutrition Board, Health and Medicine Division, National Academies of Sciences, Engineering, and Medicine, USA

## Abstract

**Background:**

Folate has been examined extensively in relation to carcinogenesis due to its role in one-carbon metabolism impacting the synthesis of DNA and RNA, methylation processes, and genomic integrity. Current evidence on the relationship between folate status and the risk of cancer is equivocal: low or deficient folate status may contribute to an increased risk of cancers, while high-dose folic acid supplementation may have adverse effects on carcinogenesis.

**Methods:**

We searched MEDLINE, Embase, CINAHL, the Cochrane Library, and the Database of Abstracts of Reviews of Effects up to February 2024 for systematic reviews and meta-analyses investigating the associations of folate (measured as dietary intake, supplementation, or blood concentrations) with any specific cancer outcome. Screening, data extraction, and risk of bias assessment were performed in duplicate. We assessed the credibility of the evidence using predefined criteria.

**Results:**

We found 67 syntheses, of which 57 provided meta-analyses. Over half of the syntheses had a high risk of bias. We identified 168 unique associations (unique exposure – unique outcome – unique setting) across 10 cancer types, 3 system cancers, and total cancer. Of these, we assessed 15 directional associations (colorectal, oesophageal, and total cancers) to be at a highly suggestive level of credibility, and 17 directional and 10 null associations to be at a suggestive level of credibility.

**Conclusions:**

The available evidence for each category of unique association was generally limited. Highly suggestive associations were found for oesophageal, colorectal, childhood brain and spinal tumours and total cancers. More robust primary studies are warranted to follow-up the signal of a positive relationship reported for prostate cancer warranting further research. Evidence was weak for all but colorectal and oesophageal cancers, or the central nervous system cancers in children.

**Registration:**

PROSPERO: CRD42021265041.

Cancer is one of the leading contributors to mortality globally [1], with the latest estimates from the World Health Organization placing it among the top five causes of death in 135 out of 185 countries in the world [2], with a total of 19.3 million cases in 2020. The incidence is projected to increase by 47% by 2040 [1]. Cancer accounted for a total of 19.3 million new cases in 2020 and approximately 17% of deaths globally, presenting a substantial disease burden. The incidence is projected to increase by 47% by 2040 [1].

Diet is a modifiable lifestyle factor that may lower the risk of cancer. Insufficient consumption of fruits and vegetables has been reported linked to a third of cancer mortality [3], while a recent modelling study suggests that up to 30% of cancer deaths may be prevented by changes in dietary choices [4]. Folate is a dietary component that has been extensively examined in the context of carcinogenesis; it is a water-soluble B vitamin found naturally in green leafy vegetables, cruciferous vegetables, citrus fruits, and nuts [5]. Foods fortified with folic acid (FA), for example, provide a highly bioavailable source that greatly enhances folate biomarker status in consumers [6]. Folate plays a critical role in one-carbon metabolism, which is required for the synthesis of DNA and RNA; it also supports methylation pathways and promotes genomic integrity and epigenetic stability [7–9]. Thus, sub-optimal folate status may potentially contribute to carcinogenesis and cancer progression [10–15]. For example, under conditions of low folate status, *de novo* synthesis of thymidylate is compromised, causing uracil misincorporation during DNA synthesis/repair, DNA mutagenesis, and consequently, malignant transformation [16,17].

Preclinical and epidemiological evidence on the relationship between folate status and cancer risk, however, is equivocal. Some studies indicated a directional link between low folate status and higher risk of cancers [7,15,18,19]. In contrast, low or deficient folate status has also been reported to be associated with lower risk of some specific types of cancers [20–26], such as colorectal [20], oesophageal [21–23,27], pancreatic [24,25], cervical [26], and squamous cell carcinoma of the head and neck [28], possibly due to growth inhibition of existing tumours. There is also some evidence to indicate that higher folate intake (particularly of the synthetic vitamin form, FA) may have adverse effects on carcinogenesis and be associated with an increased risk of breast [29,30], prostate [31], and colorectal cancer [32]. Notably one clinical trial suggested that high dose FA may promote colorectal tumorigenesis in patients with pre-existing lesions [33]. Other reports show inconsistent associations between dietary folate intake and the risks of gastric [24,27], colorectal [34], and oesophageal [25,27,35–38]. Thus, some have proposed a dual role for FA in carcinogenesis: while it may be protective against cancer at lower doses, it might stimulate the further development of existing lesions at higher doses [39].

In this umbrella review, we sought to critically synthesise the available evidence on the relationship between folate status and the risk of cancer, while delineating study populations, folate exposure measurements, and cancer subtypes to identify potential modifying factors of the associations between folate and cancer.

## METHODS

The methodological framework used in this umbrella review was described in detail in the first paper of the series [40]. Here, we briefly outline the key components of the framework. All findings are reported in accordance with the PRISMA guidelines [41].

### Data sources and search

We systematically searched MEDLINE, Embase, CINAHL, the Cochrane Library, and the Database of Abstracts of Reviews of Effects (DARE) from inception to 13 February 2024 for systematic reviews with or without meta-analyses that investigated the associations of folate, measured as dietary intake, supplementation, or blood concentrations, with any cancer outcome. The search strategy was developed and executed by a health sciences librarian (LS) in consultation with all authors who provided expertise on methodological, biological, and policy aspects of the work.

### Eligibility criteria

We included systematic reviews with or without meta-analyses that synthesised two or more component studies examining the relationship between folate intake/status and any cancers or precancerous conditions. The component studies could include randomised controlled trials (RCTs), non-randomised intervention trials, prospective or retrospective cohort studies, case-control studies, or cross-sectional studies. We excluded case reports, case series, commentaries, protocols or scoping reviews; syntheses investigating circulating homocysteine as a marker of folate status; and analyses of multivitamins or multiple nutrients without assessing the independent effect of folate. We set no restrictions on study population or date of publication. We also did not filter for articles published in non-English languages at the search stage, but did exclude them at the final screening stage to reduce potential bias in English language-based searches and to gauge the volume of non-English publications in the topical area.

### Data extraction

Two reviewers (SY, AM, or NJ) independently screened the retrieved articles. Data were extracted in duplicate (SY, AM) using a standardised and piloted (n = 10) template comprising six sections: study information, study population, exposure, outcome, qualitative synthesis, and quantitative data (Table S1 in the **Online Supplementary Document**). For syntheses reporting multiple exposures or outcomes (*e.g.* different measures of folate exposure, subcategories of cancer, subgroup analyses, sensitivity analyses, dose-response analyses, *etc*.), each exposure measure or outcome was extracted separately. Missing information was documented as ‘not reported’. Discrepancies during the screening or data extraction were resolved through discussion.

### Risk of bias assessment

Two reviewers (AM, NJ) independently assessed the quality of the syntheses using the ROBIS tool [42]. The risk of bias was assessed across four domains (study eligibility criteria, identification and selection of studies, data collection and study appraisal, and synthesis and findings), and an overall risk of bias was determined in the interpretation of review outcomes. Conflicts in the assessment were resolved through discussion. We did not generate a final score, but instead presented a descriptive summary of the levels of risk of bias across the domains.

### Synthesis

The articles were first described by specific types of exposure measure, outcome, and setting. The setting could include population subgroups (*e.g.* age, sex), geographical regions, or study population characteristics (*e.g.* individuals with a history of adenomas, current smokers). All of the associations identified from the reviews were then categorised into unique associations (unique exposure – unique outcome – unique setting). In each category of unique associations, we examined the available evidence and whether the summary effects were concordant in direction, magnitude, and statistical significance. If the summary effects were concordant, we selected the evidence with the largest number of total participants. If the summary effects were discordant, we selected studies with the largest number of total participants included in the synthesis; the largest number of cases (for binary outcomes), the recency of publication, and the highest methodological quality as assessed by ROBIS.

We considered study designs in our syntheses in two ways. First, we described all the available evidence in terms of the designs of component studies. Second, if the association selected for a unique association was entirely or predominantly based on cross-sectional or case-control studies, we also reported findings, whenever possible, from an alternative synthesis comprising entirely or predominantly prospective studies (intervention trials, prospective cohorts, nested case-control studies, or case cohort studies) as a sensitivity analysis.

We used Covidence (Covidence, Melbourne, Australia) for screening, Microsoft Excel (Microsoft, Redmond, Washington, USA) for data extraction, and SAS, version 9.4.1 (SAS Institute Inc., Cary, North Carolina, USA) for visualisation of forest plots.

### Assessment of credibility

We evaluated the credibility of evidence for all identified unique associations using a predefined set of criteria [43,44] which we subsequently modified (**Table 1**). For directional associations (*P* < 0.05), we ranked the evidence into four classes: convincing, highly suggestive, suggestive, and weak. For null associations (*P* > 0.05), we classified the evidence into two classes: suggestive and weak. If elements of information that determine the credibility of an association (*e.g.* number of total participants, number of cases, heterogeneity) were missing or incompletely reported, we downgraded the credibility by one level. For example, if the number of cases was not reported for a directional association of *P* < 0.001, we downgraded its credibility from ‘potentially suggestive’ to ‘weak’. For evidence that reported positive associations and assessed to be of a highly suggestive level of credibility, we validated, to the extent of availability of component study data, the summary effects and their 95% confidence intervals (CIs) along with between-study heterogeneity (*I*^2^) and small study effects (Egger’s test) [45]. We also calculated prediction intervals [46] to ensure the evidence meets the criteria for a convincing level of credibility.

**Table 1:** Criteria for credibility assessment

Category	Associations
**Directional associations**	
Convincing	
	With statistical significance of *P* < 10^−6^
	Based on ˃1000 cases (or ˃20 000 participants for continuous outcomes)
	For which largest component study reports a statistically significant result (*P* < 0.05) and has a 95% prediction interval that excludes the null
	Which do not have large heterogeneity (*I*^2^<50%)
	Show no evidence of small study effects (*P* ˃ 0.10) or of excess significance bias (*P* ˃ 0.10)
Highly suggestive	With statistical significance of *P* < 10^−6^
	Based on ˃1000 cases (or ˃20 000 participants for continuous outcomes)
	For which largest component study reports a statistically significant result (*P* < 0.05)
Suggestive	With statistical significance of *P* < 0.01
	Based on ˃1000 cases (or ˃20 000 participants for continuous outcomes)
Weak	With statistical significance of *P* < 0.05
**Null associations**	
Suggestive	Based on ˃1000 cases (or ˃20 000 participants for continuous outcomes)
	Which do not have large heterogeneity (*I*^2^<50%)
	With statistical significance of *P* > 0.10
Weak	With statistical significance of 0.05<*P* < 0.10

## RESULTS

### Overview of search results

Following a comprehensive search of syntheses on the topic of folate intake/status and various health outcomes, we included 287 articles for our overall series of umbrella reviews. Of these, 67 investigated the relationship between folate intake/status and the risk of various cancers (Table S2 in the **Online Supplementary Document**), with 57 providing pooled risk estimates from meta-analyses. Among the meta-analyses, breast cancer (7 syntheses, 32 unique associations), colorectal cancer (17 syntheses, 42 unique associations), and oesophageal cancer (9 syntheses, 17 unique associations) were examined in larger volumes of evidence compared to other cancer types. We identified no meta-analysis on skin cancer. Three syntheses reported on cancers of organ systems and total cancers.

Of the 168 unique associations identified across different cancer types, folate intake was investigated in 119 (71%) associations (dietary intake: 53 associations; supplement: 35 associations; total intake: 31 associations), while biomarkers of folate status were studied in 49 (29%) associations (serum/plasma: 40 associations; red blood cell (RBC): 2 associations; and combination of biomarkers: 7 associations). A total of 36 associations used aggregate measures of folate exposure (*i.e.* dietary intake/ total intake/ RBC folate concentrations); we did not count them as unique and presented them separately (Table S5 in the **Online Supplementary Document**).

Most of the syntheses categorised the folate exposure into high *vs*. low groups and reported the risks of cancer incidence or prevalence among the high folate group compared to the low group using odds ratios (OR) or relative risks (RR). Fewer studies examined biomarkers of folate status as a continuous variable and reported the risk estimates in standardised mean difference (SMD) or mean difference (MD). Below, we present the findings for colorectal and oesophageal cancers. Syntheses for other cancer types are provided in the **Online Supplementary Document**. A summary of meta-analyses for each cancer type is available in Tables S3–12 in the **Online Supplementary Document**.

Below, we present the findings for colorectal and oesophageal cancers (). We provide a summary of each synthesis in Tables S3–12 in the **Online Supplementary Document**.

### Colorectal cancer

A total of 18 syntheses [47–64] reported on the relationship between folate status and colorectal cancer and dysplasia. We identified 43 unique associations focusing on specific subgroups and settings.

Dietary intake of folate and the risk of colorectal cancer was examined in four syntheses [47–50]. High dietary folate intake was not associated with the risk of colorectal cancer in the general population (140 771 participants, 4480 cases; SMD = 0.36 µg/d; 95% CI = −0.2, 0.92; *I*^2^ = 99.5%) [49] or among women (HR = 0.93; 95% CI = 0.80, 1.08; *I*^2^ = 31%) [48].

High FA supplement intake was associated with lower risk of colorectal cancer in the general population (1 988 974 participants, 22 962 cases; RR = 0.88; 95% CI = 0.81, 0.95; *I*^2^ = 42.9%) [51]. This association was not consistently seen in subgroups: individuals with prior colorectal adenoma (2546 participants, 31 cases; RR = 0.81; 95% CI = 0.40, 1.62; *P*-value for heterogeneity <0.001), individuals with cardiovascular disease (31 192 participants, 346 cases; RR = 1.02; 95% CI = 0.83, 1.26; *P*-value for heterogeneity <0.001), or individuals of European descent (19 031 participants, 241 cases; RR = 0.91; 95% CI = 0.71, 1.17; *P*-value for heterogeneity <0.001) [52]. Among individuals with inflammatory bowel disease (IBD), high intake of FA supplement was associated with a lower risk of colorectal cancer (3607 participants, 20 cases; HR = 0.62; 95% CI = 0.41, 0.83) [53].

High combined intake of dietary folate and supplemental FA was associated with lower risk of colorectal cancer in the general population (6 165 894 participants, 37 280 cases; RR = 0.88; 95% CI = 0.83, 0.92; *I*^2^ = 34.3%; *P* = 0.05), as well as among men (RR = 0.76; 95% CI = 0.69, 0.84; *I*^2^ = 47.6%), women (RR = 0.89; 95% CI = 0.81, 0.98; *I*^2^ = 0%), individuals living in Europe (three prospective cohorts RR = 0.79; 95% CI = 0.64, 0.96; *I*^2^ = 0%), individuals living in the USA (RR = 0.86; 95% CI = 0.82, 0.90; *I*^2^ = 21.7%), individuals with high alcohol use (RR = 0.95; 95% CI = 0.92, 0.98; *I*^2^ = 55%), and individuals with medium alcohol use (RR = 0.97; 95% CI = 0.96, 0.99; *I*^2^ = 62.8%) [50]. No association was reported for individuals with no or low alcohol use (RR = 1.00; 95% CI = 0.98, 1.02; *I*^2^ = 0%) [50], nor were thresholds for categorising alcohol use.

Individuals with colorectal cancer had lower serum folate concentrations (3139 participants, 1181 cases; MD = −1.10; 95% CI = −1.60, −0.60) [54], but not plasma (8764 participants, 3515 cases; SMD = 0.01 nmol/L, 95% CI = −0.07, 0.08; *I*^2^ = 47.5%) [49].

The risk of colorectal hyperplastic polyps was inversely associated with a high dietary folate intake (33 332 participants, 1056 cases; RR = 0.65; 95% CI = 0.49, 0.85; *I*^2^ = 35%) [55], but not the risk of adenomatous polyps (1933 participants, 966 cases; SMD = 0.02 µg/d, 95% CI = −0.07, 0.11; *I*^2^ = 20.2%) [49].

High FA supplements intake did not have significant effects in lowering the risk of colorectal adenoma among individuals at risk of developing adenomas (3686 participants, 445 cases; OR = 1.09; 95% CI = 0.93, 1.29; *I*^2^ = 0%) [56] or among the general population (RR = 1.00; 95% CI = 0.86, 1.51) [57]. They also had no significant effect in lowering the risk of advanced adenoma among individuals with history of adenoma, whether used with aspirin (1870 participants, 203 cases; RR = 1.13; 95% CI = 0.84, 1.51; *I*^2^ = 21%) or without aspirin (749 participants, 104 cases; RR = 1.34; 95% CI = 0.77, 2.36; *I*^2^ = 55%) [58]. FA supplement use was associated with lower risk of colorectal dysplasia among individuals with IBD (HR = 0.63; 95% CI = 0.31, 0.94; *I*^2^ = 35.7%). In prevention of recurrence of colorectal adenomas among previously affected individuals, FA supplement use did not show significant effect whether used alone (RR = 0.93; 95% CI = 0.79, 1.25), total n = 1615; case n = 444; I^2^ = 71%) [59] or when used with aspirin (1870 participants, 647 cases; RR = 1.05; 95% CI = 0.93, 1.18; *I*^2^ = 0%) [58].

RBC, serum, or plasma folate concentrations were not significantly different in individuals with colorectal adenoma compared to healthy controls. Sun *et al*. [60] reported no differences in RBC folate (2058 participants, 790 cases; SMD = 0.28; 95% CI = −0.60, 1.15; *I*^2^ = 98.1%) and serum folate (864 participants, 241 cases; SMD = 0.04; 95% CI = −0.27, 0.36; *I*^2^ = 73.8%). A lack of noteworthy difference was also reported for plasma folate (1813 participants, 782 cases; SMD = −0.05 nmol/L; 95% CI = −0.74, 0.65; *I*^2^ = 96.5%), which remained consistent in the sensitivity analysis of prospective cohorts (885 participants, 449 cases; SMD = −0.03 nmol/L; 95% CI = −0.16, 0.1; 449; *I*^2^ = 0%) [49].

Three syntheses examined an aggregate outcome of colorectal cancer and dysplasia [49,53,57]. In the general population, use of FA supplements was not associated with a lower risk of colorectal cancer or dysplasia (1 962 281 participants, 11 096 cases; RR = 1.07; 95% CI = 0.86, 1.43) [57]. Plasma folate concentrations were not different between individuals with and without colorectal cancer or adenoma polyp (14 951 participants, 6212 cases; SMD = −0.08 nmol/L; 95% CI = −0.23, 0.08; *I*^2^ = 9.6%) [49]. Among individuals with IBD, FA supplement use was associated with lowered risk of colorectal cancer or dysplasia (4517 participants, 638 cases; HR = 0.58; 95% CI = 0.37, 0.80; *I*^2^ = 29.7%) [53]. This association remained significant in subgroups of studies conducted before FA fortification (two studies; HR = 0.47; 95% CI = 0.20, 0.75); studies assessed by the authors as of high quality using the Newcastle Ottawa Scale (eight studies; HR = 0.47; 95% CI = 0.26, 0.67); and studies conducted in the USA (seven studies; HR = 0.58; 95% CI = 0.36, 0.81) [53].

Specifically, both dietary intake (OR = 0.75; 95% CI = 0.57, 0.99; *I*^2^ = 53%) [48] and total intake of folate (RR = 0.86; 95% CI = 0.81, 0.92; *I*^2^ = 48.9%; *P* = 0.06)) [50] were associated with lower risks of colon cancer. Dietary intake (five case-control studies; OR = 0.89; 95% CI = 0.64, 1.25; *I*^2^ = 0%) [48] or total intake (RR = 0.92; 95% CI = 0.84, 1.02; *I*^2^ = 10.7%) [50] did not show significant associations with rectal cancer.

Dose-response relationship between FA supplement use and the risk of colorectal cancer was investigated by one synthesis reporting no association (RR = 0.98 per 100 mg/d increase in FA supplement; 95% CI =0.97, 1.00; *I*^2^ = 0%) [61].

The risk of early onset colorectal cancer and adverse events related to FA use were examined in systematic reviews without meta-analyses. Carroll *et al*. [65] found two case-control studies (68 254 participants) reporting significant associations in the same direction, but with different magnitudes: lower folate consumption was associated with greater risk of early onset colorectal, colon, and rectal cancer. The authors, however, noted that the association was attenuated when adjusted for age, sex, family history, and total energy consumption.

Cooper *et al*. [58] found two RCTs (749 participants) that found no difference between FA intervention and the placebo group in occurrence of serious adverse events (*e.g.* death, bleeding, stroke, myocardial infarction, vascular events, or dyspepsia). One of the two trials reported higher rates of cancer other than colorectal cancer in the intervention group, which the authors attributed to the higher baseline rate of prostate cancer in the intervention group.

### Oesophageal cancer

Nine meta-analyses [21,24,66–72] examined 17 unique associations between folate intake/status and oesophageal cancer.

High intake of dietary folate was associated with a lowered risk of oesophageal cancer (525 745 participants, 3743 cases; OR = 0.55; 95% CI = 0.43, 0.67; *I*^2^ = 61.7%) [66]. The direction and magnitude of the association remained consistent in a sensitivity analysis of prospective cohort studies (491 353 participants, 759 cases; OR = 0.51; 95% CI = 0.28, 0.94; *I*^2^ = 73%) [67]. Subgroup analyses by geographic region showed associations of similar direction and magnitude, with ORs ranging from 0.51 (95% CI = 0.40, 0.65) to 0.74 (95% CI = 0.58, 0.95) across the Americas, Europe, Australia [67], and Uruguay [24]. The pooled estimates for Asia showed an OR of 0.77 (95% CI = 0.59, 1.01; *I*^2^ = 0%) [67]. The pooled risk estimates were also comparable between the studies with a low risk of bias (4 477 445 participants, 1069 cases; OR = 0.60; 95% CI = 0.53, 0.69); *I*^2^ = 49.2%) and with a high risk of bias (1818 participants, 576 cases; OR = 0.69; 95% CI = 0.55, 0.86); *I*^2^ = 81.7%) [21].

High total folate intake was also associated with a lower risk of oesophageal cancer (493 761 participants, 1056 cases; OR = 0.69; 95% CI = 0.53, 0.85; *I*^2^ = 0%) [66].

High plasma folate concentration was associated with lower risk of oesophageal cancer generally (919 participants, 333 cases; OR = 0.71; 95% CI = 0.55, 0.92; *I*^2^ = 9.3%) and among Chinese individuals (438 participants, 187 cases; OR = 0.52; 95% CI = 0.44, 0.79; *I*^2^ = 92.5%) [21], but not among Europeans (529 participants, 152 cases; OR = 0.13; 95% CI = 0.77, 2.28; *I*^2^ = 0%) [21]. The association with high serum folate concentration was also not significant (31 901 participants, 1100 cases; OR = 0.71; 95% CI = 0.33, 1.09; *I*^2^ = 87.7%) [66].

Dietary folate was inversely associated with the risk of oesophageal adenocarcinoma (495 407 participants, 1863 cases; OR = 0.60; 95% CI = 0.51, 0.69; *I*^2^ = 34.1%) [67]. High dietary folate intake was associated with lower risk of oesophageal squamous cell carcinoma (497 653 participants, 1759 cases; OR = 0.61; 95% CI = 0.51, 0.73; *I*^2^ = 28.2%) [67], and this association was consistent in a subgroup of studies conducted in China (287 participants; HR = 0.41; 95% CI = 0.25, 0.69; *I*^2^ = 0%) [68]. High serum folate concentration was associated with a lowered risk of oesophageal squamous cell carcinoma (476 participants, 232 cases; OR = 0.44; 95% CI = 0.32, 0.61) [21].

Zhao *et al*. [69] examined the relationship between dietary folate intake and Barrett’s oesophagus and reported an inverse association (1404 participants, 371 cases; RR = 0.47; 95% CI = 0.31, 0.71; *I*^2^ = 0%). An investigation of the dose-response relationship showed that an increment of 100 μg/d in dietary folate intake was associated with 9% reduction in the risk of oesophageal cancer (25 335 participants, 1209 cases; OR = 0.91; 95% CI = 0.88, 0.94) [66].

### Risk of bias assessment

**Figure 1:**
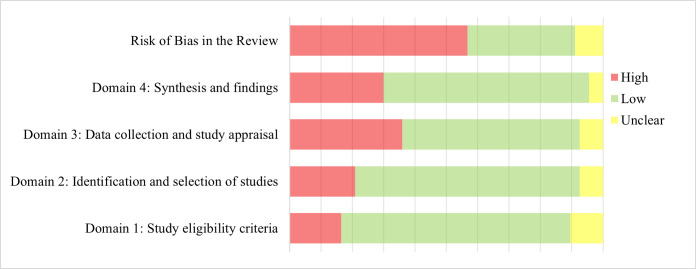
Distribution of the risk of bias across the included articles by domain.

Approximately 58% of the syntheses had a high risk of bias, 33% had a low risk of bias, and 9% had uncertain risk of bias (**Figure 1**). The overall risk of bias was determined through cumulative rating of the risks identified across the different domains as recommended by the authors [42], rather than by averaging the domain ratings. A high risk of bias identified in any one domain impacted the overall risk of bias.

At the domain level (Table S6 in the **Online Supplementary Document**), the risk of bias was generally low (73%) in definitions of scope and eligibility and selection of studies. In data abstraction and appraisal, fewer (56%) of the studies were identified to have low risk of bias. In synthesis and reporting of findings, 65% of the syntheses had low risk of bias. Items that were commonly rated high risk of bias across the syntheses were comprehensiveness of search (search strategies and number of databases searched); duplicate processes for screening and data extraction; assessment of risk of bias in the component studies including the use of formalised/validated tools; and discussion or investigation of the potential sources of heterogeneity among the studies. .

### Credibility assessment

Of the 168 unique associations, 15 and 27 were assessed to be of highly suggestive and suggestive levels of credibility, respectively (**Table 2**; Figures S1–4 and Table S4 in the **Online Supplementary Document**). A total of 48 unique associations were downgraded to a lower level of credibility because of the unavailability of key information, *i.e.* selected component studies and the number of total participants and/or cases. Most of the associations with missing information were subgroup analyses. The remaining 78 associations were assessed to be of weak credibility, primarily due to small sample sizes or high level of heterogeneity.

**Table 2:** Identified evidence on unique associations between folate intake/status and cancers and assessment of credibility*

Author (year)	Outcome (setting/subgroup)	Primary study design	Exposure	Total number (number of cases)	Metric	Summary estimate (95% CI)	*I*^2^ (%)	Credibility
Tio *et al*. (2014) [73]	Breast cancer, postmenopausal	PC, CC	Dietary folate	360 634 (15 484)	OR	0.84 (0.75, 0.94)†	62.6	Suggestive
Tio *et al*. (2014) [73]	Breast cancer, all	PC, CC	Dietary folate	608 265 (34 602)	OR	0.84 (0.77, 0.91)†	71.2	Suggestive
Chen *et al*. (2021) [74]	Breast cancer, China	PC, CC	Dietary folate	13 287 305 (>2590)	OR	0.63 (0.46, 0.85)†	78.2	Suggestive
Li *et al*. (2015) [75]	All-cause mortality, individuals with breast cancer	PC	Dietary folate	7299 (1604)	HR	0.74 (0.60, 0.92)†	35.7	Suggestive
Tio *et al*. (2014) [73]	Breast cancer, premenopausal	PC, CC	Total folate	92 682 (1600)	OR	1.10 (0.92, 1.31)	0	Suggestive
Liu *et al*. (2015) [51]	Colorectal cancer	PC	Supplement	1 988 974 (22 962)	RR	0.88 (0.81, 0.95)†	42.9	Suggestive
Fu *et al*. (2023) [50]	Colorectal cancer	PC	Total folate	6 165 894 (37 280)	RR	0.88 (0.83, 0.92)†	34.3	Highly suggestive
Fu *et al*. (2023) [50]	Colorectal cancer, men	PC	Total folate	NR (NR)	RR	0.76 (0.69, 0.84)†	47.6	Suggestive‡
Fu *et al*. (2023) [50]	Colorectal cancer, US	PC	Total folate	5 803 272 (32 786)	RR	0.86 (0.82, 0.90)†	21.7	Highly suggestive
Zhang *et al*. (2015) [54]	Colorectal cancer	CC	Serum folate	3139 (1181)	MD	−1.10 (−1.60, −0.60)†	NR	Suggestive
Shiao *et al*. (2018) [49]	Colorectal cancer	PC	Plasma folate	8764 (3515)	SMD	0.01 (−0.07, 0.08)	47.50	Suggestive
Bailie *et al*. (2017) [55]	Hyperplastic polyp	PC, CC	Dietary folate	33 332 (1056)	RR	0.65 (0.49, 0.85)†	35	Suggestive
Qiang *et al*. (2018) [67]	EAC	PC, CC	Dietary folate	495 407 (1863)	OR	0.60 (0.51, 0.69)†	34.1	Highly suggestive‡
Qiang *et al*. (2018) [67]	ESCC	PC, CC	Dietary folate	497 653 (1759)	OR	0.61 (0.51, 0.73)†	28.2	Highly suggestive‡
Larsson *et al*. (2006) [24]	Oesophageal cancer, Uruguay	CC	Dietary folate	5177 (1430)	RR	0.62 (0.53, 0.72)†	0.00	Highly suggestive‡
Zhao *et al*. (2017) [21]	Oesophageal cancer, NOS≥7	PC, CC	Dietary folate	4 477 445 (1069)	OR	0.60 (0.53, 0.69)†	49.2	Highly suggestive‡
Liu *et al*. (2017) [66]	Oesophageal cancer	PC, CC	Dietary folate	525 745 (3743)	OR	0.55 (0.43, 0.67)†	61.7	Highly suggestive
Qiang *et al*. (2018) [67]	Oesophageal cancer, Americas	CC	Dietary folate	498 551 (2159)	OR	0.58 (0.51, 0.67)†	37.3	Highly suggestive‡
Qiang *et al*. (2018) [67]	Oesophageal cancer, Europe	CC	Dietary folate	4668 (1482)	OR	0.51 (0.40, 0.65)†	49.8	Highly suggestive‡
Liu *et al*. (2017) [76]	Oesophageal cancer	PC, CC	Total folate	493 761 (1056)	OR	0.69 (0.53, 0.85)†	0	Suggestive
Liu *et al*. (2017) [76]	Gastric cancer	PC, CC	Dietary folate	826 498 (6026)	OR	0.71 (0.59, 0.84)†	71.8	Suggestive
Liu *et al*. (2017) [76]	Gastric cancer	PC	Total folate	554 820 (1095)	OR	0.88 (0.65, 1.12)	0	Suggestive
Wang *et al*. (2021) [77]	Ovarian cancer	PC, CC	Dietary folate	230 240 (5885)	RR	0.90 (0.77, 1.06)	38.8	Suggestive
Wang *et al*. (2021) [77]	Ovarian cancer	PC, CC	Total folate	240 493 (4320)	RR	1.06 (0.89, 1.27)	42.8	Suggestive
Tio *et al*. (2014) [78]	Prostate cancer	PC, CC	Dietary folate	146 782 (15336)	OR	0.97 (0.89, 1.06)	41.9	Suggestive
Tio *et al*. (2014) [78]	Prostate cancer	PC, CC	Total folate	93 781 (7114)	OR	0.99 (0.82, 1.19)	48.2	Suggestive
Collin *et al*. (2010) [79]	Prostate cancer	PC, CC	Plasma/serum/RBC folate	9778 (2958)	OR	1.11 (0.96, 1.28)	40	Suggestive
Fan *et al*. (2017) [80]	Head and neck cancer	CC	Dietary folate	12 743 (3462)	OR	0.42 (0.34, 0.50)†	7.1	Highly suggestive‡
Fan *et al*. (2017) [80]	Laryngeal cancer	CC	Dietary folate	6957 (1659)	OR	0.48 (0.34, 0.62)†	0	Suggestive
Yang *et al*. (2018) [81]	Lung cancer	NCC, CC	Serum folate	14 853 (6995)	SMD	−0.53 (−0.70, −0.35)†	89.4	Suggestive
Yang *et al*. (2018) [81]	Lung cancer, Europe	NCC, CC	Serum folate	NR (NR)	SMD	−0.23 (−0.30, −0.16)†	0	Highly suggestive‡
Yang *et al*. (2018) [81]	Lung cancer, Asia	CC	Serum folate	NR (NR)	SMD	−0.84 (−1.01, −0.67)†	0	Highly suggestive‡
Yang *et al*. (2018) [81]	Lung cancer, former smokers	NCC, CC	Serum folate	NR (NR)	OR	0.70 (0.62, 0.79)†	32.8	Highly suggestive‡
Chiavarini *et al*. (2018) [82]	Childhood brain and spinal cord tumours, before/during pregnancy	PC, CC	Maternal supplement	694 685 (2994)	OR	0.77 (0.66, 0.90)†	53.2	Suggestive
Chiavarini *et al*. (2018) [82]	Childhood brain and spinal cord tumours, before/during pregnancy	PC, CC	Maternal total folate	695 647 (3475)	OR	0.77 (0.78, 0.88)†	51.2	Highly suggestive
Wan Ismail *et al*. (2019) [83]	Acute lymphoblastic leukaemia, before/during pregnancy	CC	Maternal supplement	18 405 (6570)	OR	0.75 (0.66, 0.86)†	62	Suggestive
Zhang *et al*. (2015) [71]	Digestive system cancer	NR	Serum folate	5063 (1823)	MD	−2.61 (−2.98, −2.25)†	NR	Highly suggestive‡
Zhang *et al*. (2015) [71]	Genital system cancer	CC	Serum folate	9631 (4571)	MD	−1.65 (−2.45, −0.85)†	NR	Suggestive
Zhang *et al*. (2015) [71]	Respiratory system cancer	CC	Serum folate	3744 (1401)	MD	−2.11 (−3.15, −1.07)†	NR	Suggestive
Chen *et al*. (2021) [74]	Total cancer, China	PC, CC	Dietary folate	561 538 (10 073)	OR	0.73 (0.61, 0.88)†	75	Suggestive
Zhou *et al*. (2011) [84]	Total cancer, individuals with CV/renal conditions	RCT	Supplement	26 544 (2472)	RR	1.08 (0.98, 1.21)	26.7	Suggestive
Wien *et al*. (2012) [85]	Total cancer mortality	RCT	Supplement	32 327 (1134)	RR	1.09 (0.87, 1.22)	45	Suggestive
Zhang *et al*. (2015) [71]	Total cancer	CC	Serum folate	21 696 (9047)	MD	−2.68 (−3.21, −2.15)†	NR	Highly suggestive
Zhang *et al*. (2015) [71]	Total cancer, Europe	CC	Serum folate	10 692 (4870)	MD	−1.17 (−1.55, −0.79)†	NR	Highly suggestive‡
Zhang *et al*. (2015) [71]	Total cancer, Asia	CC	Serum folate	2923 (1157)	MD	−4.65 (−5.82, −3.47)†	NR	Highly suggestive‡
**Dose-response relationship**								
Liu *et al*. (2017) [66]	Oesophageal cancer	CC	Dietary folate	25 335 (1209)	OR	0.91 (0.88, 0.94) for every 100 μg/d increase†	0.02	Highly suggestive
Fan *et al*. (2017) [80]	Head and neck cancer	CC	Dietary folate	7281 (1969)	OR	0.96 (0.94, 0.98) for every 100 μg/d increase†	NR	Suggestive
Liu *et al*. (2017) [76]	Pancreatic cancer	PC	Dietary folate	418 612 (1113)	OR	0.94 (0.92, 0.97) for every 100 μg/d increase†	NR	Suggestive

ALL – acute lymphoblastic leukaemia, AML – acute myeloid leukaemia, CC – case-control study, EAC – oesophageal adenocarcinoma, EC – oesophageal carcinoma, ER – oestrogen receptor, ESCC – oesophageal squamous cell carcinoma, GAC – gastric cardiac adenocarcinoma, GPC – gastric precancerous condition, HER2 – human epidermal growth factor receptor 2, HR – hazards ratio, MD – mean difference, NR – not reported, OR – odds ratio, PDAC – pancreatic ductal adenocarcinoma, PR – progesterone receptor, RR – relative risk, SMD – standardised mean difference

*Associations between composite folate exposure measures (dietary and biomarkers) and cancers are presented in Table S1 in the **Online Supplementary Document**.

†Statistically significant associations.

‡Credibility levels downgraded due to unavailability of data.

A highly suggestive level of evidence was identified for oesophageal cancer (seven associations), colorectal cancer (two associations), head and neck cancer (one association), childhood brain and spinal cord tumours (one association), digestive system cancer (one association), and total cancer (three associations). Eight out of the 15 associations examined dietary folate intake, three examined total folate intake, and the other four examined serum folate concentrations. Significant inverse associations were demonstrated between high dietary folate intake and a lowered risk of oesophageal adenocarcinoma (OR = 0.60; 95% CI = 0.51, 0.69), oesophageal squamous cell carcinoma (OR = 0.61; 95% CI = 0.51, 0.73), and oesophageal cancer overall (OR = 0.55; 95% CI = 0.43, 0.67). These associations were consistent in studies conducted in Uruguay (RR = 0.62; 95% CI = 0.53, 0.72), Americas (OR = 0.58; 95% CI = 0.51, 0.67), and Europe (OR = 0.51; 95% CI = 0.40, 0.65); among high quality observational studies (OR = 0.60; 95% CI = 0.53, 0.69). High intake of dietary folate was also significantly associated with a lower risk of head and neck cancer (OR = 0.42; 95% CI = 0.34, 0.50). Higher total folate intake was associated with a lower risk of colorectal cancer overall (RR = 0.88; 95% CI = 0.83, 0.92) and in the USA (RR = 0.86; 95% CI = 0.82, 0.90)). Higher maternal total folate intake was associated with a lower risk of brain and spinal cord tumours in the offspring (OR = 0.77; 95% CI = 0.78, 0.88)). Lower serum folate concentration was associated with an increased risk of digestive system cancer (MD = −2.61; 95% CI = −2.98, −2.25)) and a higher risk of total cancer overall (MD = −2.68; 95% CI = −3.21, −2.15)) and in Europe (MD = −1.17; 95% CI = −1.55, −0.79)) and Asia (MD = −4.65; 95% CI = −5.82, −3.47)).

Of the 28 suggestive associations, 17 were directional and 10 were null. Inverse association was reported between high dietary folate intake and a lower risk of breast cancer in general (OR = 0.84; 95% CI = 0.77, 0.91), which was consistent among premenopausal women (OR = 0.84; 95% CI = 0.75, 0.94) and women in China (OR = 0.63; 95% CI = 0.46, 0.85). High dietary folate intake was also associated with a decreased risk of hyperplastic colorectal polyps (RR = 0.65; 95% CI = 0.49, 0.85), gastric cancer (OR = 0.71; 95% CI = 0.59, 0.84), laryngeal cancer (OR = 0.48; 95% CI = 0.34, 0.62), and total cancer (OR = 0.73; 95% CI = 0.71, 0.88). FA supplementation was associated with a lower risk of colorectal cancer (RR = 0.88; 95% CI = 0.81, 0.95). High total folate intake was associated with lower risks of oesophageal cancer (OR = 0.69; 95% CI = 0.53, 0.85) and colorectal cancer among men (RR = 0.76; 95% CI = 0.69, 0.84). Lower serum folate concentration was associated with an increased risk of colorectal cancer (MD = −1.10; 95% CI = −1.60, −0.60)), lung cancer (SMD = −0.53; 95% CI = −0.70, −0.35), genital system cancer (MD = −1.65; 95% CI = −2.45, −0.85), and respiratory system cancer (MD = -2.11; 95% CI = −3.15, −1.07). Maternal supplementation with FA was associated with lower childhood brain and spinal cord tumours (OR = 0.77; 95% CI = 0.66, 0.90) and acute lymphoblastic leukaemia (OR = 0.75; 95% CI = 0.66, 0.86) in the offspring.

Null associations were identified in 10 relationships measured in large samples (>1000 cases) and in studies with low heterogeneity (*I*^2^ < 50%). Dietary folate intake was not significantly associated with ovarian cancer (RR = 0.90; 95% CI = 0.77, 1.06) or prostate cancer (OR = 0.97; 95% CI = 0.89, 1.27); FA supplement use was not significantly associated with total cancer incidence (RR = 1.0; 95% CI = (0.98, 1.21) or total cancer mortality (RR = 1.09; 95% CI = 0.87, 1.22). Total folate intake was not significantly associated with the risks of breast cancer among premenopausal women (OR = 1.10; 95% CI = 0.92, 1.31), gastric cancer (OR = 0.88; 95% CI = 0.65, 1.12), ovarian cancer (OR = 1.06; 95% CI = 0.89, 1.27), or prostate cancer (OR = 0.99; 95% CI = 0.82, 1.19). Plasma folate concentration was not significantly associated with the risk of colorectal cancer (SMD = 0.01; 95% CI = −0.07, 0.08) or prostate cancer (OR = 1.11; 95% CI = 0.96, 1.28). The associations assessed to be highly suggestive or suggestive are visualised in Figures S1–4 in the **Online Supplementary Document**.

Three dose-response relationships were available for credibility assessment. One association was assessed to be highly suggestive: the risk of oesophageal cancer decreased per 100 μg/d increase in dietary folate intake (OR = 0.91; 95% CI = 0.88, 0.94). Two associations were at a suggestive level: the risk of head and neck cancer decreased per 100 μg/d increase in dietary folate intake (OR = 0.96; 95% CI = 0.94, 0.98) and the risk of pancreatic cancer decreased per 100 μg/d increase in dietary folate (OR = 0.94; 95% CI = 0.92, 0.97).

## DISCUSSION

### Summary of findings

We examined systematic reviews and meta-analyses investigating the relationship between folate intake/status and various cancers and precancerous conditions. Overall, 168 unique associations were identified, synthesised, and critically appraised. Of these, 15 directional associations were assessed to be highly suggestive and 27 (17 directional and 10 null) were suggestive. Of the reported dose-response relationships, one was assessed to be highly suggestive and two others were deemed as suggestive. The effect sizes in the dose-response were small. Overall risk of bias in these syntheses was medium to high, with more syntheses rated as high risk compared to those with low risk. Most of the syntheses did not account for risk of bias in their analyses by conducting sensitivity analyses or subgroup analyses.

The evidence landscape was somewhat uneven across cancer types, with a substantial volume of evidence concentrated on breast, colorectal, and oesophageal cancers. More subgroup analyses (by histologic type or underlying comorbidities) were available for these three cancer types compared to others. This bias in the evidence may simply reflect the most common cancers in the world in terms of incidence and mortality [1,86].

We did not identify multiple syntheses for each unique association, with most of the unique associations being reported by one or two articles. While the direction of the identified associations was in alignment in almost all categories of unique associations, the magnitude of the pooled point estimates and the level of heterogeneity varied, likely due to the differences in the study populations or designs of the component studies. Of the 168 selected unique associations, all but four associations indicated an inverse relationship between folate intake/status and the risk of cancer. These four associations were: plasma/serum folate concentration and ER+breast cancer risk [87]; FA supplement and prostate cancer risk [88]; serum folate concentration and prostate cancer risk [38]; and plasma/serum folate concentration and prostate cancer risk [31]. All these associations were assessed to be of weak credibility due to small or unreported sample size. Nevertheless, we note that these positive associations were reported predominantly for prostate cancer and warrant further research, for example on the potential role of non-metabolised FA.

Among highly suggestive associations, the evidence was consistent in the direction of a beneficial effect of dietary/total folate intake for oesophageal cancer (seven associations) and colorectal cancer (two associations). The evidence for other cancers was largely inconsistent with variations in the magnitude of associations across subgroups of individuals or subtypes of cancer. It is possible that folate may play a more crucial role in maintaining DNA integrity and facilitating DNA repair in rapidly proliferating gastrointestinal epithelia (*e.g.* colon and oesophagus) [89] compared to tissues with slower cell turnover (*e.g.* breast) or with less dependence on one-carbon metabolism [90].

Triangulated evidence, where reported associations from intake and biomarker measures aligned, was identified for five cancer outcomes. The risk of colorectal cancer in the general population was inversely associated with FA supplement use, total folate intake, and serum folate concentration; the risk of oesophageal cancer was inversely associated with dietary intake, total intake, and plasma concentration; the risk of oesophageal squamous cell carcinoma was inversely associated with dietary intake and serum concentration; and the risk of total cancer was inversely associated with dietary intake, supplement, and serum concentration. The risk of prostate cancer was positively associated with supplement use and plasma/serum concentration.

### Critical appraisal

Dietary intake was the most used measurement of folate exposure (32%), followed by biomarkers (29%). Meanwhile, FA supplementation was assessed in only 21% of the associations and mainly related to colorectal cancer and childhood cancer. We note potential errors and imprecision that are inherently present in dietary intake measurements, which are typically collected from self-reported food frequency questionnaires or intake diaries [91]. Systematic reviews included in our synthesis did not report on adjustment of measurement errors or adjustment for energy intake. Imprecision of dietary intake measures may be further compounded by varying levels of intake of FA fortified foods, which have higher bioavailability and should be weighed differently, ideally in dietary folate equivalent (DFE) units. The unit of the dietary or total folate intake was not reported in most of the syntheses.

Some syntheses reported associations using aggregated measures of exposure, such as combining dietary intake and total intake, dietary or total intake with biomarkers, or plasma folate and RBC folate concentrations. We also observed aggregation of outcomes, such as combining carcinoma and adenoma outcomes, where the authors did not provide rationale or methodological approach for the aggregation. Without careful consideration of the nature of each exposure measure and a robust approach to combine these, an aggregate exposure measure may introduce heterogeneity into the analysis.

A predominant share of the identified syntheses was limited to observational studies. Component RCTs were few and these were found only in colorectal cancer (prevention of recurrence or progression to advanced stage), gastric cancer (prevention of precancerous lesions), and prostate cancer (primary prevention). While observational research provides valuable information on potential relationships between exposures and outcomes in real world settings, confounders are often not sufficiently adjusted for and therefore pooled risk estimates may include some spurious effects. Limited contribution to the evidence by the RCTs also resulted in a smaller number of inquiries into the role of FA supplement in the treatment or prevention of specific cancer or precancerous conditions in their onset or recurrence.

We did not consider study design in our credibility assessment; however, for each unique association, we selected synthesis comprised of prospective studies, whenever possible. If a selected synthesis was comprised entirely of retrospective studies, we reported the findings from a synthesis in the same category that pooled from a smaller sample of prospective studies. Nevertheless, due to the small volume of evidence in each category, we could not conduct sensitivity analyses in some associations.

### Equity and global health in the evidence on cancers

The current evidence was derived from studies conducted in Europe, North America, and East Asia. We did not identify any systematic review with specific coverage of low- and middle-income countries, which is a concern in the light of the recent reports showing the majority of cancer incidence and mortality, especially of high-burden cancers, occurs in such contexts [86,92]. Resources required for cancer detection and treatment are severely lacking in these countries [93]. Inclusion of research in such countries would add to the evidence base by increasing the range of exposure, diversity of covariates and enabling consistency of association to be assessed in situations where confounding structures are different from those in high-income countries.

### Policy and research implications

Knowledge on the relationship between folate status and the risk of different cancers is still in development. More robust prospective studies are needed to assess risks across different sociodemographic factors, underlying comorbidities, and geographical regions. Current evidence is not sufficient to inform targeted policy interventions such as food fortification with folic acid for the prevention of cancer.

Policy discussion on folic acid fortification has been under way in many countries for several years. Decreases in colorectal cancer diagnoses and mortality have been observed in North America and Australia, where mandatory FA fortification to prevent NTDs has been in place for 30 years and 15 years, respectively [94,95]. FA fortification has also been reported to be associated with reductions in the incidence of neuroblastoma and Wilms tumour in children in Ontario, Canada [96,97] and in the USA [98]. While these ecological trends provide tentative signals on the potential of FA for prevention of certain cancers, they should be interpreted with caution when applying to more specific contexts. We recommend generating more data from rigorously designed primary studies that examine not only the benefits of folate in prevention of cancers but also the potential harms of unmetabolised folic acid.

### Limitations

We highlight two overall limitations of this umbrella review. First, baseline folate status in the study population was not reported in any of the syntheses that examined folate intake (dietary intake or total intake or supplementation) as exposure. It has been reported that the benefits of folate may be substantially reduced or become less detectable among individuals who are folate adequate [99]. We could not identify from the syntheses whether the study populations had inadequate levels of folate at baseline, or if the pooled estimates were different according to the baseline folate status. We also could not identify the timeline of collection of exposure data relative to the cancer diagnosis in most of the studies. Second, detailed information on the characteristics of the study population were often not available. Thus, information on the distribution of age, sex, and country of the participants was provided only in some synthesis and was mostly missing from subgroup analyses.

## CONCLUSIONS

We identified 168 unique associations between folate and the risks of various cancers from the existing systematic reviews and meta-analyses. The available evidence for each cancer type was generally limited with the evidence concentrated on a few cancers with high global prevalence. Most of the associations indicated an inverse relationship between folate and the risk of cancer. We assessed 15 associations to be of highly suggestive levels of credibility: low folate status and increased risk of oesophageal, colorectal, digestive system, and total cancer; and low maternal folate intake and increased risk of childhood brain and spinal cord tumours. We detected a tentative, but concerning positive signal for an elevated risk of prostate cancer associated with FA supplementation, which warrants targeted investigation. For other cancers, the pooled estimates were largely inconsistent and require well-designed primary studies.

## Additional material


Online Supplementary Document
