# Supplementary material for: Folate and global health umbrella review series, part 2: syntheses on cancers
Source: J Glob Health. 2026 Feb 20;16:04102. doi: 10.7189/jogh.16.04102 (PMC12922470; doi:10.7189/jogh.16.04102)
Supplement: Online Supplementary Document [file jogh-16-04102-s001.pdf]

Supplement to: Yoo S, Montazeri A, Bennett D, Bo Y, Chen P, Duthie S, Jensen N, Kaminga A, Lai J, Li X, MacFarlane AJ, Martinez H, McNulty H, Momoli F, Mossey P, Mullie P, Munger R, Parajuli RP, Kent MP, Rubini M, Senekal M, Sikora L, Stintzi A, Theodoratou E, Wang H, Yajnik C, Yaktine A, Little J. Folate and global health umbrella review series, part 2: syntheses on cancers. J Glob Health. 2026;16:04102.

Breast cancer

Seven articles reported on eight associations between folate status and breast cancer. From these, we identified 32 unique associations that focused on specific subgroups, i.e., menopausal status, hormone receptor subtype, geographic region, and lifestyle.

High intake of dietary folate was associated with lower incidence of breast cancer among women regardless of menopausal status (OR=0.84 (0.77, 0.91); total n=181,199; case n=34,602; I^2=71.2%)[1]. A sensitivity analysis comprised only of prospective cohort studies showed an attenuated association (RR=0.95 (0.97, 1.03); total n=634,304; case n=21,830; I^2=66.2%) in the same direction[2].

The direction and magnitude of the association was comparable between premenopausal (OR=0.81 (0.66, 1.00); total n=181,199; case n=6,376; I^2=69%) and postmenopausal women (OR=0.84 (0.75, 0.94); total n=360,634; case n=15,484; I^2=62.6%)[1]. Both estimates were attenuated in the sensitivity analyses of prospective studies (OR=1.02 (0.62, 1.68) and OR=0.93 (0.81, 1.07) for pre- and post-menopausal subgroups, respectively)[1].

Stratified analyses by hormone receptor subtype were reported by two articles. Across different estrogen receptor (ER) and progesterone receptor (PR) status (ER+, ER-, PR+, PR-, ER+ PR+, ER+ PR-, ER- PR+, ER- PR-), the association between dietary folate and the risk of breast cancer was not significant, ranging from OR=0.81 (0.53, 1.24) to OR=0.97 (0.81, 1.17)[1,2]. The subgroup analyses by hormone receptor subtype were based on small numbers of component studies and showed a comparable direction of association.

A focused analysis on Chinese women showed an inverse association between intake of dietary folate and risk of breast cancer (OR=0.63 (0.46, 0.85); total n=13,286,305; I^2=78.2%)[3]; while an analysis on North American women did not show an association (OR=0.98 (0.90, 1.08); I^2=53.3%)[2]. A subgroup of studies that adjusted for alcohol use also reported a non-significant association (RR=0.95 (0.86, 1.06); I^2=71.1%)[2].

The use of FA supplements appeared to be not associated with the risk of breast cancer (RR=1.19 (0.94, 1.18); I^2=48.3%)[4]. Meanwhile, a high total intake of folate, a combined measure of dietary folate and FA supplements, was significantly associated with a lowered risk of overall breast cancer in women (OR=0.85 (0.79, 0.92); total n=1,436,020; case n=51,120; I^2=75.2%)[5]. This association was attenuated in a sensitivity analysis of prospective studies (OR=0.97 (0.91, 1.03); total n=1,405,320; case n=37,917); I^2=53.3%)[5]. The total folate intake was not associated with breast cancer in the subgroups of premenopausal or postmenopausal women or ER/PR status[1]. When considering dietary or supplementary folate as the exposure, the association remained non-significant (OR=0.94 (0.88, 1.00); total n=1,171,048; case n=41,516; I^2>0.05).

Serum, plasma, or /plasma concentrations of folate were not associated with the risk of breast cancer (OR=1.04 (0.76, 1.42), OR=0.94 (0.82, 1.17), OR=0.86 (0.60, 1.25), respectively)[1,2,5]. Plasma/serum concentration of folate was not associated with the risk of ER+ or ER- breast cancer among 2,100 women (OR=1.59 (1.19, 2.12), OR=1.02 (0.52-2.00), respectively)[1].

Li et al. (2015)[6] examined the association between dietary or total folate intake and mortality among women with breast cancer. Intake of dietary folate was inversely associated with all-cause mortality (HR=0.74 (0.60, 0.92); total n=7,299; case n=1,604; I^2=35.7%), but not with breast cancer mortality (HR=0.79 (0.61, 1.01); total n=4,624; case n=505; I^2=0%). High total intake of folate was not associated

with all-cause mortality among women with breast cancer (HR=0.93 (0.75, 1.15); total n=4,183; case n=752; I^2=0%).

Dose-response analyses were available from three syntheses[2,4,5]. The risk of breast cancer decreased marginally with a 100 µg/d increment of total folate intake (OR=0.98 (0.97, 0.99))[5], but not associated with a 100 µg/d increase in dietary intake (RR=1.00 (0.98, 1.00))[2] or 5 ng/mL increase in plasma folate concentration (OR=0.99 (0.94, 1.04)). Sensitivity analyses showed comparable findings for total intake (OR=0.99 (0.98, 1.00))[5] and plasma folate (OR=1.10 (0.90, 1.36))[5]. A 100 µg/d increment in any measure of folate intake lowered the risk of breast cancer of ER- subtype (RR=0.94 (0.88, 0.99)) or ER-PR- subtype (RR=0.90 (0.85, 0.97))[4].

Gastric cancer

Five articles examined folate and its relationship with gastric cancer and precancerous lesions. Six unique associations were identified.

High intake of dietary folate was inversely associated with the risk of gastric cancer (OR=0.71 (0.59, 0.84); total n=826,498; case n=6,026; I^2=71.8%)[7]; however, this relationship was not replicated in a sensitivity analysis of prospective cohort studies (OR=1.19 (0.92, 1.54); total n=197,159; case n=776; I^2=0%)[8]. The risk of gastric cancer was not significantly associated with total folate intake (OR=0.88 (0.65, 1.12); total n=554,820; case n=1,095; I^2=0%)[7] or serum folate concentration (OR=1.22 (0.48, 1.96); total n=31,640; case n=855; I^2=58.6%)[7].

Lei et al. 2022[9] pooled findings from RCTs conducted in China that examined the effectiveness of using FA supplements, alone or with other therapies, on treatment of gastric precancerous conditions (chronic atrophic gastritis or intestinal metaplasia). FA supplement use was not more effective in relieving clinical symptoms of precancerous conditions (RR=1.12 (0.96, 1.13); total n=914; case n=482; I^2=63%); however, it was more effective in treatment of gastric mucosa atrophy lesions (RR=0.62 (0.42, 0.94); total n=503; case n=191; I2=69%) and treatment of intestinal metaplasia lesions (RR=0.57 (0.42, 0.76); total n=186; case n=89; I^2=0%) compared to placebo or routine therapies[9].

One dose-response analysis reported that the risk of gastric cancer was not strongly associated with a 100 µg/d increase in dietary folate intake (OR=0.99 (0.97, 1.00); total n=266,299; case n=2,897)[7]. In addition, FA supplementation at 20-30 mg/d for 3-6 months was effective in positive pathological changes of gastric precancerous lesions, while a regimen of 30 mg/d for 3 months had superior therapeutic effect on positive pathological changes of precancerous mucosa[9].

Genitourinary cancers

We identified 11 syntheses with meta-analyses examining 12 unique associations between folate intake/status and a group of genitourinary cancers. The specific types of cancers investigated were cervical cancer (2 articles), endometrial cancer (1 article), ovarian cancer (3 articles), and prostate cancer (5 studies).

Serum folate concentrations were lower among women with cervical cancer compared to healthy controls (OR=0.52 (0.30, 0.88); total n=2,383; case n=873; I^2=81%)[10] and this association remained consistent among the women in Asian countries (OR=0.29 (0.17, 0.49); total n=617; case n=275; I^2=17.7%) but not among women in the US (OR=0.90 (1.27, 0.64); total n=1,766; case n=598; I^2=44.5%)[10].

Dietary folate intake was examined in relation to the risk of endometrial cancer. High dietary intake did not show significant associations with the risk (OR=0.89 (0.76, 1.05); total n=270,542; case n=6,151;

I^2=59%). This association remained non-significant in the sensitivity analysis (OR=1.05 (0.90, 1.21); total n=259,997; case n=1,977; I^2=19.8%)[11] and in the subgroup analysis of North America (OR=0.89 (0.76, 1.05); total n=265,753; case n=4,408; I^2=54.9%)[11].

Ovarian cancer risk was not associated with dietary folate intake (RR=0.90 (0.77, 1.06); total n=230,240; case n=5,885; I^2=38.8%)[12]. The direction and magnitude of the association remained consistent in the sensitivity analysis (RR=0.84 (0.61, 1.14); total n=217,309; case n=1,158; I^2=40.8%) and in the subgroup analysis of North America (RR=0.88 (0.76, 1.03); total n=160,124; case n=2,881; I^2=23.8%)[13]. Total folate intake also showed non-significant association with the risk of ovarian cancer (RR=1.06 (0.89, 1.27); total n=240,483; case n=4,320; I^2=42.8%)[12].

For prostate cancer, the risk was not significantly associated with dietary folate intake (OR=0.97 (0.89, 1.06); total n=146,782; case n=15,336; I^2=41.9%)[14] or with total folate intake (OR=0.99 (0.82, 1.19); total n=93,781; case n=7,114; I2=48.2%)[14]. Both associations remained consistent in the sensitivity analyses (OR=1.00 (0.96, 1.05) and OR=1.00 (0.79, 1.27), respectively).

FA supplementation showed a direct relationship with the risk of prostate cancer (RR=1.24 (1.03, 1.49); total n=25,738; case n=632; I^2=17%) based on six RCTs[15]. High serum folate (RR=1.21 (1.05, 1.39); total n=9,810; I^2=0%)[16] and high plasma/serum folate (OR=1.43 (1.06, 1.93); total n=10,232; case n=6,122; I^2=79.5%)[14] were associated with higher risk of prostate cancer. The relationship between high plasma/serum/RBC folate concentration and prostate cancer was not significant when pooled across study designs (OR=1.11 (0.96, 1.28); total n=9,778; case n=2,958; I^2=40%)[17]; however, the association was stronger in a sensitivity analysis of prospective studies (OR=1.18 (1.00, 1.40); total n=6,810; case n=1,497; I^2=13%)[17]. Individuals included in the component studies of these syntheses were aged 49-80 years and mostly resided in Western Europe, North America, and Australia. Baseline folate status was not reported in any syntheses.

Head and neck cancer

Two syntheses[18,19] reported on the associations between folate status and different types of head and neck cancer. Five unique associations were identified and each of these was examined by a single meta-analysis of case-control studies.

High dietary intake of folate was associated with a lowered risk of head and neck cancer (OR=0.42 (0.34, 0.50); total n=12,743; case n=3,462; I^2=7.1%)[18]. The inverse relationship was not significant when measured with serum folate concentration (OR=0.80 (0.56, 1.04); total n=2,149; case n=628; I^2=18.3%)[18].

Low serum folate concentration was associated with a higher risk of head and neck squamous cell carcinoma in a smaller meta-analysis (MD= -3.97 (-4.62, -3.31); total n=905; case n=335)[19].

Higher dietary folate intake was associated with a lowered risk of nasopharyngeal carcinoma (OR=0.47 (0.15, 0.79); total n=1,992; case n=798; I^2=67.5%)[18] and a lowered risk of laryngeal cancer (OR=0.48 (0.34, 0.62); total n=6,957; case n=1,659; I^2=0%)[18].

A dose-response analysis was reported by one meta-analysis[18] pooling four case-control studies: the risk of head and neck cancer was lowered per 100 µg/d increase in dietary folate intake (OR=0.96 (0.94, 0.98); total n=7,281; case n=1,969).

Hepatocellular cancer

One article synthesized five small-sized case-control studies that examined the relationship between serum folate concentration and hepatocellular carcinoma[19]. The risk of hepatocellular carcinoma was associated with lower serum folate status (MD= -4.60 (-5.93, -3.26); total n=376; case n=152).

Kidney cancer

Three meta-analyses provided pooled estimates of the risk of kidney cancer associated with folate status. Four unique associations were identified. High intake of dietary folate was not significantly associated with the risk of kidney cancer (RR=0.83 (0.70, 1.00); total n=139,728; case n=4,145)[20] or with the risk of renal cell carcinoma (RR=0.85 (0.71, 1.01); total n=135,821; case n=3,264)[20]. The risk of renal cell carcinoma was significantly associated with lower serum folate concentration (MD= -0.95 (-1.90, -0.00); total n=1,013; case n=391) [19], but not with lower plasma/serum folate concentration (RR=0.87 (0.72, 1.05); total n=133,707; case n=2,297)[21].

Lung cancer

We identified three syntheses reporting on nine unique associations between folate status and the risk of lung cancer. High dietary folate intake was not significantly associated with the risk (OR=0.97 (0.77, 1.23); I^2=0%) in the Chinese population[3]; however, low serum folate concentration was associated with a higher risk (SMD= -0.53 (-0.70, -0.35); total n=14,853; case n=6,995; I^2=89.4%)[22]. Subgroup analyses were reported on the risk of lung cancer in relation to serum folate concentrations. The direction of association remained the same across all subgroups (Europe, Asia, male, female, never smoker, former smoker, and current smoker) with varying magnitudes among Europe (SMD= -0.23 (-0.30, -0.16); I^2=0%), Asia (SMD= -0.84 (-1.01, -0.67); I^2=0% (p=0.32)), male (OR= 0.82 (0.73, 0.92); I^2=25.9%), former smokers (OR=0.70 (0.62, 0.79), I^2=32.8%) and current smokers (OR=0.86 (0.75, 0.99); I^2=27.9%)[22].

Pancreatic cancer

Five meta-analyses examined the association between folate status and the risk of pancreatic cancer and reported on six unique associations. The risk of pancreatic cancer was lower with high intake of dietary folate (OR=0.67 (0.45, 0.89); total n=475,924; case n=2,659)[7]. This association remained consistent in a sensitivity analysis of four prospective cohorts (RR=0.52 (0.36, 0.75); total n=237,153; case n=618; I^2=17.1%)[23]. High intake of FA supplements was not significantly associated with the risk (RR=1.08 (0.82, 1.41); I^2=13.6%)[24], while high total folate intake was associated with a lowered risk of pancreatic cancer (OR=0.76 (0.56, 0.95); total n=180,888; case n=1,407; I^2=39.8%)[7].

Serum folate concentration (OR=0.76 (0.34, 1.19); total n=521,998; case n=1,011; I^2=65.7%)[7] or plasma/serum folate concentration (RR=0.80 (0.44, 1.45); I^2=71.5%)[24] were not significantly associated with the risk of pancreatic cancer. Serum folate concentration was not significantly associated with the risk of pancreatic ductal adenocarcinoma (RR=0.82 (0.52, 1.30); I^2=61%)[25].

In a dose-response analysis, Liu et al.[7] reported that the risk of pancreatic cancer decreased per 100 µg/d increase in dietary folate intake (OR=0.94 (0.92, 0.97); total n=418,612; case n=1,113).

Skin cancer

We found one systematic review that reported on the relationship between dietary folate intake and the risks of basal cell carcinoma and squamous cell carcinoma[26]. The risks were examined from two prospective cohorts of the general population including male health professionals in France and the US. In

basal cell carcinoma, high dietary folate intake was reported to be associated with increasing the risk. In squamous cell carcinoma, two studies reported conflicting results.

Childhood cancers

We identified four syntheses reporting on 18 unique associations between childhood cancers and folate intake/status in mothers or children. Component studies were predominantly retrospective studies.

The risk of childhood brain and spinal cord tumors was not associated with maternal intake of dietary folate before/during pregnancy (OR=0.76 (0.53, 1.07); I^2=55.7%) or during pregnancy (OR=0.69 (0.48, 1.01); I^2=51.9%)[27]; but the risk was inversely associated with maternal supplementation of FA before pregnancy (OR=0.64 (0.50, 0.81); I^2=2.3%), during pregnancy (OR=0.80 (0.67, 0.97); I^2=62.48%), and before/during pregnancy (OR=0.77 (0.66, 0.90); total n=694,685; case n=2,994; I^2=53.2%)[27]. Chiavarini et al. 2018[27] reported inverse relationships between maternal total folate intake and the risk of central nervous system and miscellaneous intracranial and intraspinal neoplasms (OR=0.82 (0.68, 0.99); I^2=62.6%), intracranial and intraspinal embryonal tumors (OR=0.70 (0.54, 0.90); I^2=37.8%), and low-grade glioma (OR=0.55 (0.39, 0.79); I^2=0%). The association between maternal total folate intake and the risk of astrocytoma among the offspring was not significant (OR=0.93 (0.63, 1.38); I^2=0%).

The risk of Wilms tumor was lowered with high maternal intake of dietary folate (OR=0.79 (0.69, 0.91); total n=50,290,620; case n=965; I^2=0%)[28].

High maternal intake of FA supplements was associated with a lowered risk of acute lymphoblastic leukemia (ALL) among the offspring (OR=0.75 (0.66, 0.86); total n=18,405; case n=6,570; I^2=62%)[29]. This association was significant for the maternal supplementation before pregnancy (OR=0.69 (0.50, 0.95); total n=10,327; case n=3,511; I^2=56.8%) but not during pregnancy (OR=0.87 (0.57, 1.34); total n=10,245; case n=3,346; I^2=89.3%)[30]. The risk of ALL was not significantly associated with the child's use of FA supplements (OR=0.79 (0.53, 1.18); total n=1,906; case n=540; I^2=0%)[30]. Acute myeloid leukemia (AML) risk was not significantly associated with maternal FA supplementation (OR=0.70 (0.46, 1.06); total n=5,627; case n=577; I^2=55%)[29].

Total cancer and organ system cancers

Three articles reported on the risks of cancers by organ system and total cancers, from which 15 unique associations were identified. Digestive system cancers consisted of colorectal cancer, pancreatic cancer, esophageal cancer, hepatocellular cancer, gastric cancer, and cholangiocarcinoma; respiratory system cancers consisted of lung cancer and laryngeal squamous cell carcinoma; and urinary system cancers consisted of renal carcinoma, bladder cancer, and urothelial carcinoma. Female reproductive system cancers were comprised of endometrial and ovarian cancers.

The risk of digestive system cancers was not associated with intake of dietary folate among Chinese individuals (OR=0.91 (0.67, 1.22); I^2=74.4%)[3], but was associated with lower serum folate concentration (MD= -2.61 (-2.98, -2.25); total n=5,063; case n=1,823)[19] based on 16 case-control studies. The risk of respiratory system cancers was higher in low serum folate concentrations (MD= -2.11 (-3.15, -1.07); total n=3,744; case n=1,401)[19]. Urinary system cancers were not associated with the serum folate concentrations (MD= -2.09 (-5.15, 0.96); total n=2,056; case n=724)[19]. Lower serum folate concentrations were also associated with increased risks of genital system cancers (MD= -1.65 (-2.45, -0.85); total n=9,631; case n=4,571)[19] and female reproductive system cancers among Chinese women (OR=0.59 (0.46, 0.75); I^2=0%)[3].

With regard to all cancers combined, high dietary folate intake was associated with a lower risk (OR=0.73 (0.61, 0.88); total n=561,538; case n=10,073; I^2=75%)[3]; however, this association was attenuated in a sensitivity analysis of seven prospective cohorts (OR=0.93 (0.82, 1.07); I^2=0%)[3]. Higher use of FA supplements was not significantly associated with total cancer incidence (RR=1.07 (1.00, 1.14); total n=38,233; case n=3,515; I^2=0%) or mortality (RR=1.09 (0.87, 1.22); total n=32,327; case n=1,134; I^2=45%) compared to the placebo in randomized controlled trials[15]. The non-significant association between FA supplement and total cancer incidence was also observed among individuals with underlying cardiovascular or renal conditions (RR=1.08 (0.98, 1.21))[31]. Based on 40 case-control studies, Zhang et al.[19] reported low serum folate concentrations were associated with the risk of all cancers (MD= -2.68 (-3.21, -2.15); total n=21,696; case n=9,047). This association was directionally consistent in the subgroup analyses by geographic region with varying magnitudes in Europe (MD= -1.17 (-1.55, -0.79); total n=10,692; case n=4,870), Asia (MD= -4.65 (-5.82, -3.47); total n=2,923; case n=1,157), the Middle East (MD= -1.40 (-2.40, -0.41); total n=1,728; case n=614), in America (MD= -0.24 (-0.54, 0.07); total n=3,548; case n=1,387)[19].

One dose-response analysis limited to China reported that the risk of all cancers was lowered per 100 µg/d increase in the intake of dietary folate (OR=0.86 (0.80, 0.92))[3].

References

1. Tio M, Andrici J, Eslick GD. Folate intake and the risk of breast cancer: A systematic review and meta-analysis. Breast Cancer Res Treat. 2014;145(2):513–24.
2. Chen P, Li C, Li X, Li J, Chu R, Wang H. Higher dietary folate intake reduces the breast cancer risk: A systematic review and meta-analysis. Br J Cancer. 2014;110(9):2327–38.
3. Chen Y, Zhan J, Wang Y, Chen S. Association between dietary intake of folate and the risks of multiple cancers in Chinese population: A dose-response meta-analysis of observational studies. Nutr Cancer. 2021;73(9):1644–56.
4. Zeng J, Wang K, Ye F, Lei L, Zhou Y, Chen J, et al. Folate intake and the risk of breast cancer: an up-to-date meta-analysis of prospective studies. Eur J Clin Nutr. 2019 Dec 1;73(12):1657–60.
5. Ren X, Xu P, Zhang D, Liu K, Song D, Zheng Y, et al. Association of folate intake and plasma folate level with the risk of breast cancer. Aging. 2020;12(21):21355-21375.
6. Li B, Lu Y, Wang L, Zhang CX. Folate intake and breast cancer prognosis: A meta-analysis of prospective observational studies. European Journal of Cancer Prevention. 2015;24(2):113–21.
7. Liu W, Zhou H, Zhu Y, Tie C. Associations between dietary folate intake and risks of esophageal, gastric and pancreatic cancers: an overall and dose-response meta-analysis. Oncotarget. 2017;8(49):86828-86842.
8. Tio M, Andrici J, Cox M, Eslick G. Folate intake and the risk of upper gastrointestinal cancers: A systematic review and meta-analysis. J Gastroenterol Hepatol. 2014;29:250–8.
9. Lei J, Ren F, Li W, Guo X, Liu Q, Gao H, et al. Use of folic acid supplementation to halt and even reverse the progression of gastric precancerous conditions: a meta-analysis. BMC Gastroenterol. 2022 Dec 1;22(1).
10. Zhou X, Meng Y. Association between serum folate level and cervical cancer: A meta-analysis. Arch Gynecol Obstet. 2016;293:871–7.
11. Du L, Wang Y, Zhang H, Zhang H, Gao Y. Folate intake and the risk of endometrial cancer: A meta-analysis. Oncotarget. 2016;7(51):85176-85184.
12. Wang K, Zhang Q, Yang J. The effect of folate intake on ovarian cancer risk: A meta-analysis of observational studies. Medicine. 2021;100:E22605.

13. Li C, Chen P, Hu P, Li M, Li X, Guo H, et al. Folate intake and MTHFR polymorphism C677T is not associated with ovarian cancer risk: Evidence from the meta-analysis. Mol Biol Rep. 2013 Dec;40(12):6547–60.
14. Tio M, Andrici J, Cox M, Eslick G. Folate intake and the risk of prostate cancer: A systematic review and meta-analysis. Prostate Cancer Prostatic Dis. 2014;17(3):213–9.
15. Wien TN, Pike E, Wisløff T, Staff A, Smeland S, Klemp M. Cancer risk with folic acid supplements: A systematic review and meta-analysis. BMJ Open. 2012;2:e000653.
16. Wang R, Zheng Y, Huang J, Zhang A, Zhou Y, Wang J. Folate intake, serum folate levels, and prostate cancer risk: A meta-analysis of prospective studies. BMC Public Health. 2014;14:1326.
17. Collin SM, Metcalfe C, Refsum H, Lewis SJ, Zuccolo L, Smith GD, et al. Circulating folate, vitamin B<inf>12</inf>, homocysteine, vitamin B <inf>12</inf> transport proteins, and risk of prostate cancer: A case-control study, systematic review, and meta-analysis. Cancer Epidemiology Biomarkers and Prevention. 2010;19(6):1632–42.
18. Fan C, Yu S, Zhang S, Ding X, Su J, Cheng Z. Association between folate intake and risk of head and neck squamous cell carcinoma: An overall and dose-response PRISMA meta-analysis. Medicine. 2017;96(42):e8182.
19. Zhang D, Wen X, Wu W, Guo Y, Cui W. Elevated homocysteine level and folate deficiency associated with increased overall risk of carcinogenesis: Meta-analysis of 83 case-control studies involving 35,758 individuals. PLoS One. 2015 May 18;10(5).
20. Clasen JL, Heath AK, Scelo G, Muller DC. Components of one-carbon metabolism and renal cell carcinoma: a systematic review and meta-analysis. Eur J Nutr. 2020 Dec 1;59(8):3801–13.
21. Mao B, Li Y, Zhang Z, Chen C, Chen Y, Ding C, et al. One-carbon metabolic factors and risk of renal cell cancer: A meta-analysis. PLoS One. 2015 Oct 29;10(10).
22. Yang J, Li H, Deng H, Wang Z. Association of one-carbon metabolism-related vitamins (Folate, B6, B12), homocysteine and methionine with the risk of lung cancer: Systematic review and meta-analysis. Frontiers in Oncology. 2018;8:493.
23. Larsson S, Giovannucci E, Wolk A. Folate intake, MTHFR polymorphisms, and risk of esophageal, gastric, and pancreatic cancer: A meta-analysis. Gastroenterology. 2006;131(4):1271–83.
24. Lin H, An Q, Wang Q, Liu C. Folate intake and pancreatic cancer risk: An overall and dose-response meta-analysis. Public Health. 2013;127(7):607–13.
25. Kumar S, Santos RJ, McGuigan AJ, Singh U, Johnson P, Kunzmann AT, et al. The role of circulating protein and metabolite biomarkers in the development of pancreatic ductal adenocarcinoma (PDAC): A systematic review and meta-analysis. Cancer Epidemiology Biomarkers and Prevention. 2022;31:1090–102.
26. Hezaveh E, Jafari S, Jalilpiran Y, Zargarzadeh N, Mahdavi R, Gargari BP. Dietary components and the risk of non-melanoma skin cancer: A systematic review of epidemiological studies. Critical Reviews in Food Science and Nutrition. 2023;63:5290–305.
27. Chiavarini M, Naldini G, Fabiani R. Maternal folate intake and risk of childhood brain and spinal cord tumors: A systematic review and meta-analysis. Neuroepidemiology. 2018;51:82–95.
28. Doganis D, Katsimpris A, Panagopoulou P, Bouka P, Bouka E, Moschovi M, et al. Maternal lifestyle characteristics and Wilms tumor risk in the offspring: A systematic review and meta-analysis. Cancer Epidemiol. 2020 Aug 1;67.
29. Wan Ismail WR, Rahman RA, Rahman NAA, Atil A, Nawi AM. The protective effect of maternal folic acid supplementation on childhood cancer: A systematic review and meta-analysis of case-control studies. Journal of Preventive Medicine and Public Health. 2019;52: 205–13.

30. Dessypris N, Karalexi MA, Ntouvelis E, Diamantaras AA, Papadakis V, Baka M, et al. Association of maternal and index child's diet with subsequent leukemia risk: A systematic review and meta-analysis. Cancer Epidemiology. 2017;47:64–75.
31. Zhou YH, Tang JY, Wu MJ, Lu J, Wei X, Qin YY, et al. Effect of folic acid supplementation on cardiovascular outcomes: A systematic review and meta-analysis. PLoS One. 2011;6(9).

Table S1. Template used to extract data from the included syntheses examining the relationship between folate intake/status and cancers

Study identifier	First author
	Year of publication
	Journal
	Title of the review
Study information	Type of synthesis (narrative synthesis, meta-analysis)
	Design of component studies
	Years searched
	Inclusion and exclusion criteria
	Number of studies included for synthesis
	Total number of participants included for synthesis
	Countries where component studies were conducted
	Characteristics of the study population (age, sex, comorbidities)
	Guidelines used for reporting
	Protocol registration
	Risk of bias assessment tool used
Exposure	Type of exposure measure
	Details of exposure measurement (FFQ, 24-h food diary, serum sample, etc.)
	Details of exposure intervention (dose, duration)
	Time of exposure measurement
Outcome	Outcome(s) examined in the review
	Details of outcome ascertainment
Findings	Authors' findings (narrative synthesis)
Meta-analysis	Number of studies included in the meta-analysis
	Model (random-effects, fixed effects)
	Total sample pooled
	Number of cases pooled
	Type of metric used (OR, RR, HR, SMD, etc.)
	Pooled estimates
	Adjustment for covariates
	Heterogeneity (I^2 and 95% CI)
	Small study effects (Egger's test, Begg's test, 95% CI)

Table S2. Characteristics of the evidence syntheses examining the relationship between folate intake/status and cancers (chronological)

First author (year)	Synthesis type	Study population	Exposure*	Outcome	No. of studies (Design)	Total (Case)	Country
Zeegers (2004)	SR	Individuals aged 40-75 years	Dietary intake	Bladder cancer	2 PC, 1 CC	169,428 (1,294)	Netherlands, US
Sanjoaquin (2005)	MA	Not described	Dietary intake	Colorectal cancer	8 PC	NR (2,394)	Netherlands, US, Canada
					10 CC	15,842 (6,166)	USA, Italy, France, Switzerland
		Not described	Total intake	Colorectal cancer	5 PC	NR (2,689)	US
					4 CC	2,467 (968)	Finland, US
Garcia-Closas (2005)	SR	NR	Dietary intake	HPV persistence	1 PC	201 (131)	"All but two studies were carried out in developed countries, mainly in the US"
			Dietary intake	Cervical neoplasia	2 PC, 5 CC	2,614 (754)	
			Plasma	HPV persistence	2 PC	215 (129)	
			Plasma/serum/ RBC	Cervical neoplasia	1 PC, 10 CC	3,943 (1,297)	
Larsson (2006)	MA	Not described	Dietary intake	EAC	3 CC	1,769 (501)	US
		Not described	Dietary intake	ESCC	4 CC	3,408 (929)	US, Japan, Italy
		Not described	Dietary intake	EC	7 CC	5,177 (1,430)	Uruguay
		Not described	Dietary intake	Gastric cancer	2 PC	64,994 (438)	Netherlands, Sweden
					9 CC	8,431 (2,767)	Italy, US, Mexico, Venezuela, Poland, Korea
		Not described	Dietary intake	Pancreatic cancer	4 PC<br>1 CC	237,153 (618)<br>357 (104)	Australia, Sweden, US, Finland
Davies (2006)	MA	Individuals with colorectal preinvasive lesions	Supplement (1-5 mg/d for 12-24 months)	Colorectal preinvasive lesion recurrence	2 RCT	77 (22)	NR
Ryan-Harshman (2007)	SR	NR	Supplement	Colorectal cancer	4 PC, 1 NCC, 3 CC	NR (NR)	NR
Cooper (2010)	MA	Individuals with history of adenoma	Supplement (0.5-5.0 mg/d for 3 years)	Colorectal adenoma recurrence	3 RCT<br>With aspirin<br>No aspirin	 1,870 (647)<br>749 (278)	US, UK
			Supplement	Advanced colorectal adenoma	2 RCT<br>With aspirin<br>No aspirin	 749 (203)<br>749 (104)	US, UK

Ibrahim (2010)	MA	Individuals aged 57-64 years with previous resection of colorectal adenomas	Supplement (0.5-1 mg/d for 1-3 years)	Colorectal adenoma recurrence	5 RCT	1,468 (564)	NR
Kubo (2010)	SR	Individuals with esophageal adenocarcinoma	Dietary intake	EAC	4 CC	2,200 (NR)	US
Collin (2010)	MA	Not described	Plasma/serum /RBC	Prostate cancer	6 PC	6,810 (1,497)	Finland, Sweden, Australia, Germany, Greece, Italy, Netherlands, Spain, UK, US, Norway
Kennedy (2011)	MA	Not described	Dietary intake (at enrolment and 5.3-22 years)	Colorectal cancer	11 PC	NR (NR)	US, Majorca, Italy, France, Finland, Switzerland, Japan, China, Australia, UK, Korea, Sweden, Netherlands, Canada
			Dietary intake (1-2 years preceding diagnosis or at enrolment)	Colorectal cancer	17 CC	3,276 (NR)	
Fife (2011)	MA	Individuals at risk of adenoma	Supplement (0.5-1 mg/d for 3 years)	All colorectal adenoma	2 RCT	33,686 (445)	US, UK
				Advanced colorectal adenoma	2 RCT	1,840 (203)	
Myung (2011)	MA	Women aged 18-84 years with cervical neoplasm	Dietary/serum /RBC	Cervical cancer	9 CC	5,203 (1,757)	US, Thailand, Poland, 4 Latin American countries
Zhou (2011)	MA	Individuals with cardiovascular or renal diseases	Supplement (0.5-40 mg/d for 8.3-87.6 months)	Total cancer	6 RCT	26,544 (2,472)	NR
Wien (2012)	MA	Individuals aged 57(10) - 69(7) years	Supplement	Total cancer	10 RCT	38,233 (3,515)	Denmark, US, Norway, Europe, Canada
		Individuals aged 62(11) - 64(9) years	Supplement	Total cancer mortality	6 RCT	32,327 (1,134)	Norway, Europe, Canada, Scotland
		Individuals aged 63(11) - 69(7) years	Supplement	Prostate cancer	6 RCT	25,738 (632)	US, UK, Europe, Canada, Norway
Misotti (2013)	SR	Postmenopausal women aged 50-76 years	Supplement 150-1400 µg/d	Breast cancer	2 PC	61,247 (1,815)	US, Denmark
					1 RCT	25,400 (691)	

		Premenopausal women aged >40 years	Supplement ≥ 400 µg/d	Breast cancer	1 CC	608 (297)	US
Vollset (2013)	MA	Individuals with previous adenoma, aged 64(10) years	Supplement (0.5-1 mg/d for 2.4-7.4 years)	Colorectal adenoma	3 RCT	2,652 (168)	US, UK
Li (2013)	MA	Not described	Dietary intake	Ovarian cancer	4 PC	217,309 (1,158)	Sweden, Canada, US
					4 CC	11,600 (4,519)	Italy, Australia, US, China
Lin (2013)	MA	Adults	Dietary intake	Pancreatic cancer	7 PC, 3 CC	NR (NR)	Europe, US, Canada, Australia
			Supplement		3 PC, 1 CC	NR (NR)	
			Plasma/serum		1 PC, 2 CC	NR (NR)	
Wang (2014)	MA	Men aged 40-79 years at baseline	Dietary intake (FU:4-17.3 years)	Prostate cancer	5 PC	192,702 (NR)	Finland, US, Australia, Netherlands, Denmark
		Men aged 49.1-69 years at baseline	Serum (FU: 4.9-15.7 years)	Prostate cancer	5 NCC	9,810 (NR)	Europe, Australia, Norway, Sweden, Finland
Chen (2014)	MA	Not described	Dietary intake	Breast cancer	15 (PC, NCC)	744,068 (26,205)	Canada, US, Australia, France, Sweden, Denmark, New Zealand, China, UK, Italy, Greece
					25 CC	38,646 (16,826)	US, Germany, China, Mexico, Uruguay, Italy, Switzerland, UK, Japan, Korea, Brazil
			Supplement	Breast cancer	3 PC	NR (24,151)	US, Denmark
			Total intake	Breast cancer	11 (PC, NCC)	187,106 (38,034)	US, Sweden, Denmark
					3 CC	54,305 (24,151)	Germany, US, Uruguay, Mexico, UK, China
Liu (2014)	MA	Women aged 26-87 years	Dietary intake	Breast cancer	15 PC	1,836,566 (24,083)	US, Canada, Australia, Denmark, France, Sweden, China
Crane (2014)	SR	Women aged 59-42 years at diagnosis, 50-56 years at baseline	Dietary intake	Incident ovarian cancer	4 PC	226,295 (NR)	Sweden, Canada, US
Tio (2014)	MA	Postmenopausal women	Dietary intake	Breast cancer	9 PC	349,285 (13,096)	France, Sweden, US, Canada, Denmark, China
					11 CC	14,486 (5,545)	Mexico, Japan, US, Korea, Switzerland, Brazil, China, Germany
		Premenopausal women	Dietary intake	Breast cancer	3 PC	172,314 (3,123)	China, Canada, US
					10 CC	16,047 (5,941)	Japan, China, US, Brazil, Switzerland, Korea, Mexico
		Postmenopausal women	Total intake	Breast cancer	7 PC	293,425 (10,165)	US, Denmark, Japan

		Premenopausal women			1 PC, 1 CC	92,682 (1,600)	
		All women	Plasma/serum	Breast cancer	5 PC, 2 CC	5,226 (2,403)	US, Australia, Sweden
Tio (2014)	MA	Not described	Dietary intake	Gastric cancer	3 PC<br>8 CC	197,159 (776)<br>12,530 (3,638)	Uruguay, Netherlands, US, China, Korea, Sweden, Italy, Serbia, Poland, Mexico, Venezuela, Europe
		Not described	Dietary intake	Pancreatic cancer	5 PC<br>3 CC	291,958 (1,247)<br>3,568 (962)	Australia, Italy, Europe, US, China, Netherlands, Sweden, Finland
		Not described	Dietary intake	EAC	3 CC	3,546 (1,019)	Uruguay, US, Switzerland, Italy, Australia, Iran, China, Japan
				ESCC	4 CC	3,977 (829)	
Tio (2014)	MA	Men aged 27-80 years	Dietary intake	Prostate cancer	5 PC<br>6 CC	140,428 (12,898)<br>6,354 (2,438)	Uruguay, Australia, Canada, US, Italy, Denmark, Netherlands, Serbia, Finland
		Men aged 50-86 years	Total intake	Prostate cancer	2 PC<br>3 CC	92,692 (6,729)<br>1,089 (385)	Denmark, US, Serbia
		Men aged 40-80 years	Plasma/serum	Prostate cancer	6 PC, 1 CC	10,232 (6,112)	Australia, UK, Sweden, Jamaica, Europe, Norway, Finland
Li (2015)	MA	Women with breast cancer, aged 54-65.7 years	Dietary intake (1-20 years prior to diagnosis)	Mortality from breast cancer	2 PC	4,624 (505)	US, Sweden
		Women with breast cancer, aged 58.8-65 years	Dietary intake (1-20 years prior to diagnosis)	All-cause mortality	5 PC	7,299 (1,604)	
			Total intake (1-20 years prior to diagnosis)	All-cause mortality	4 PC	4,183 (752)	
Zhang (2015)	MA	Individuals aged ≥18 years	Serum	All cancers	40 CC	21,696 (9,047)	NR
				Digestive system cancer	16 CC	5,063 (1,823)	
				Genital system cancer	10 CC	9,631 (4,571)	
				Respiratory system cancer	7 CC	3,744 (1,401)	
				Urinary system cancer	5 CC	2,056 (724)	
				Colorectal cancer	11 CC	3,139 (1,181)	
				Pancreatic cancer	4 CC	1,018 (288)	

				Esophagus cancer	4 CC	276 (107)	
				Hepatocellular carcinoma	5 CC	376 (152)	
				Gastric cancer	3 CC	254 (95)	
				Breast cancer	5 CC	2,199 (1,076)	
				Lung cancer	5 CC	3,519 (1,316)	
				Head & neck squamous cell carcinoma	3 CC	905 (335)	
				Renal cell carcinoma	3 CC	1,013 (391)	
				Prostate cancer	3 CC	7,290 (3,428)	
Mao (2015)	MA	Not described	Dietary/ plasma/serum	Renal cell carcinoma	2 PC, 1 NCC, 1 CH, 2 CC	133,707 (2,297)	Netherlands, US, Finland, Italy, 10 European countries
Qin (2015)	MA	Individuals aged mean 57-68.9 years with underlying risks	Supplement (0.5-2.5 mg/d for 27-88 months)	Colorectal cancer	8 RCT	34,598 (381)<br>Prior adenoma 31 (NR)<br>Prior CVD 346 (NR)	
Liu (2015)	MA	Individuals aged 30-79 years	Supplement	Colorectal cancer	23 PC	1,988,974 (22,962)	US, Australia, Denmark, Japan, Sweden, China, Netherlands
Heine-Broring (2015)	MA	Individuals aged 30-75 years	Total intake	Colorectal cancer	3 PC	291,006 (4,066)	US, Denmark
Van Dijk (2016)	MA	Colorectal cancer survivors in remission and individuals with history of colorectal adenoma	Supplement (0.5-5mg/d for 3-8 years)	Colorectal adenoma recurrence	4 RCT	1,615 (444)	North America, Europe
Zhou (2016)	MA	Individuals aged 18-74 years	Serum	Cervical cancer	6 CC	2,383 (873)	US, Thailand, China, India
Du (2016)	MA	Women aged 18-96 years	Dietary intake	Endometrial cancer	5 PC<br>9 CC	259,997 (1,977)<br>10,545 (4,174)	Canada, UA, Mexico, China, Italy, Switzerland
Burr (2017)	MA	Individuals with IBD	Supplement	Colorectal cancer or any dysplasia	4 PC, 6 CC	4,517 (638)	US, UK, Netherlands
Bailie (2017)	MA	Individuals aged ≥ 18 years	Dietary intake	Hyperplastic polyps	2 PC, 1 CC	33,332 (1,056)	US
Fan (2017)	MA	Not described	Dietary intake	Head & neck cancer	7 CC	12,743 (3,462)	China, Italy, Europe, Uruguay, US, Japan
			Serum	Head & neck cancer	2 CC	2,149 (628)	

Ni (2017)	MA	Individuals aged 18-89 years	Dietary intake	EC	2 PC<br>16 CC	NR (242)<br>15,820 (3,650)	US, China, Japan, Ital, Switzerland, Uruguay, Australia, Iran, Northern Ireland, Germany
		Individuals aged 39-70 years	Serum	EC	5 CC	2,044 (700)	China, Europe
Zhao et al. (2017)	MA	Not described	Dietary intake	EC	1 PC<br>14 CC	4,471,303 (21)<br>7,692 (1,676)	Ireland, China, Iran, Australia, Uruguay, US, Italy, Japan, Switzerland, Germany
			Plasma/serum	EC	5 CC	919 (333)<br>1,404 (371)	China, Europe
Liu et al. (2017)	MA	Not described	Dietary intake	EC	11 PC+CC	NR (NR)	US, China, Europe, Northern Ireland, Italy, Iran, Australia, Uruguay, Japan, Germany
			Total intake	EC	3 PC+CC	NR (NR)	
			Serum	EC	5 PC+CC	NR (NR)	
			Dietary/total/ serum	EAC	6 PC+CC	NR (NR)	
			Dietary/total/ serum	ESCC	7 PC+CC	NR (NR)	
			Dietary intake	Gastric cancer	18 PC+CC	NR (NR)	NR
			Total intake		2 PC+CC	NR (NR)	
			Serum		4 PC+CC	NR (NR)	
			Dietary intake	Pancreatic cancer	8 PC+CC	NR (NR)	Europe, Italy, US, Netherlands, Sweden, Finland, Canada
			Total intake		5 PC+CC	NR (NR)	
			Serum		3 PC+CC	NR (NR)	
Dessypris (2017)	MA	Mothers and children aged 0-19 years	Maternal supplement (before pregnancy)	Acute lymphoblastic leukemia	3 CC	10,327 (3,511)	“Multinational”, Israel, France
			Maternal supplement (during pregnancy)		3 CC	10,245 (3,346)	
		Children aged 10-19 years	Child supplement		3 CC	1,906 (540)	Israel, New Zealand, Australia
Ma et al. (2018)	MA	Not described	Dietary/plasma/ serum	EC	24 combined	NR (NR)	China, Ireland, Iran, Australia, Uruguay, US, Italy, Japan, Switzerland, Germany, Europe
Shiao (2018)	MA	Not described	Dietary intake	Colorectal cancer	8 PC<br>12 CC	140,771 (4,480)<br>17,524 (7,312)	NR
			Dietary intake	Adenoma polyp	2 PC<br>7 CC	1,933 (966)<br>4,622 (2,142)	
			Supplement	Colorectal cancer	3 CC	3,582 (1,637)	

			Plasma/serum	Colorectal cancer	9 PC<br>8 CC	8,764 (3,515)<br>3,393 (1,466)	
			Plasma/serum	Adenoma polyp	2 PC<br>6 CC	885 (449)<br>1,813 (782)	
Chiavarini (2018)	MA	Mothers and children aged 0-15 years	Maternal dietary/supplement (before or during pregnancy)	Childhood brain and spinal cord tumors	1 PC, 9 CC	695,647 (3,475)	France, Norway, Australia, Spain, Sweden, Italy, US, Canada
Qiang (2018)	MA	Individuals aged 50-71 years	Dietary intake	EC	2 PC	491,353 (759)	US
		Individuals aged 18-80 years			9 CC (hospital based)	9,579 (2,354)	Japan, Uruguay, China, Italy, Switzerland, Iran
		Individuals aged 30-79 years			10 CC (population based)	5,809 (2,045)	US, Australia, Ireland, Germany
		Individuals aged 18-85 years	Dietary intake	EAC	1 PC, 5 CC	495,407 (1,863)	US, Germany, Ireland, Australia, Greece
		Individuals aged 18-79 years	Dietary intake	ESCC	1 PC, 10 CC	497,653 (1,759)	Italy, Iran, Greece, France, Switzerland, Australia, Uruguay US, China, Germany
Yang (2018)	MA	Not described	Serum	Lung cancer	2 NCC	13,366 (6,255)	UK, Germany, Italy, Spain, Netherlands, Greece, Sweden, France, US, Australia, Finland, Norway, Singapore
					10 CC	1,487 (740)	China, Taiwan, New England, Turkey, Poland
Moazzen (2018)	MA	Not described	Supplement (0.5-5 mg/d for 27-110.4 months)	Colorectal cancer	4 RCT	31,192 (346)	NR
				Adenoma	10 RCT	5,896 (236)	
			Supplement (FU 5-22 years)	Colorectal cancer	3 PC	1,926,520 (10,514)	
		Not described	Dietary/total/RBC	Colorectal cancer	20 PC<br>22 CC	2,520,112 (NR)<br>12,042 (NR)	
Sun (2018)	MA	Not described	Serum	Colorectal cancer	6 CC	864 (241)	Greece, Australia, China, US
					3 CC	492 (118)	China
			RBC	Colorectal cancer	9 CC	2,058 (790)	Greece, Ireland, Australia, US
					4 CC	654 (131)	Australia
Ismail (2019)	MA	Mothers and children aged 0-19 years	Maternal supplement	Acute lymphoblastic leukemia	11 CC	18,405 (6,570)	US, Canada, Germany, Australia, France, Israel

		Mothers and children aged 0-20 years	Maternal supplement	Acute myeloid leukemia	5 CC	5,627 (577)	"North America", Germany, US, France
		Mothers and children aged 0-19 years	Maternal supplement	Brain tumor	6 CC	10,105 (2,665)	US, France, Israel, "Europe", Germany, Sweden, Spain, Australia
Zeng (2019)	MA	Not described	Dietary intake	Breast cancer	19 PC	NR (NR)	NR
			Supplement	Breast cancer	4 PC	NR (NR)	
			Total intake	Breast cancer	10 PC	NR (NR)	
			Plasma	Breast cancer	4 PC	NR (NR)	
Sun (2020)	MA	Individuals with ESCC	Dietary intake	ESCC mortality	2 PC	287 (NR)	China
Doganis (2020)	MA	Mothers and children aged 0-14 years	Maternal dietary (during pregnancy)	Wilms tumor	2 CS, 1 PC, 1 CC	50,290,620 (965)	Canada, Norway, US, Greece
Clasen et al. (2020)	MA	Individuals aged 20-85 years	Dietary intake	Renal cell carcinoma	2 PC, 3 CC, 2 NCC, 1 CH	135,821 (3,264)	US, Italy, Finland, Europe, Netherlands
			Plasma/serum		2 NCC	NR (780)	
		Individuals aged 20-89 years	Dietary intake	Kidney cancer	2 PC, 5 CC, 2 NCC, 1 CH	139,728 (4,145)	US, Italy, Finland, Europe, Netherlands, Uruguay
Ren et al. (2020)	MA	Women aged 26-87 years	Total intake	Breast cancer	19 PC	1,436,020 (51,120)	France, Europe, Italy, Australia, China, US, Canada, Denmark, Sweden
		Women aged 18-89 years		Breast cancer	20 CC	30,700 (13,203)	Canada, US, Japan, China, Korea, Denmark, Mexico, Germany, England, Switzerland, Uruguay
		Women aged 18-70 years	Plasma	Breast cancer	2 PC	979 (815)	Sweden, Canada
		Women aged 20-84 years			10 CC	16,748 (7,850)	US, Europe, Italy, Uganda, Sweden, Taiwan, Australia
Nucci (2021)	SR	Not described	Dietary intake	EAC	1 PC	492,292 (574)	US
				EAC	3 CC	1,988 (629)	US, Ireland
Zhao (2021)	MA	Not described	Dietary intake	Barrett's esophagus	2 CC		US, Ireland
Wang (2021)	MA	Women aged 18-85 years	Dietary intake	Ovarian cancer	4 PC, 6 CC	230,240 (5,885)	US, Sweden, China, Canada, Italy, Mexico, Australia
		Women aged 18-79 years	Total intake		4 PC, 2 CC	240,493 (4,320)	US, Australia
Khodavandi (2021)	MA	Women aged 27-93 years	Dietary intake (FU 8.1-16.4 years)	Ovarian cancer	9 PC	NR (NR)	NR

Hezaveh (2021)	SR	General population including male healthcare professionals	Dietary intake	Squamous cell carcinoma	2 PC	173,229 (NR)	US
			Dietary intake	Basal cell carcinoma	2 PC	178,580 (NR)	US, France
Chen (2021)	MA	Individuals aged 45.7-61.4 years	Dietary intake	All cancers	7 PC, 9 CC	561,538 (10,073)	China
		Individuals aged 45.7-52.5 years	Dietary intake	Breast cancer	1 PC, 4 CC	78,626 (NR)	
		Individuals aged 52.5-61.4 years	Dietary intake	Digestive system cancer	3 PC, 2 CC	212,825 (NR)	
		Women aged 47.2-54 years	Dietary intake	Female reproductive system cancer	2 CC	2,510 (NR)	
		Individuals aged 52.3-55.3 years	Dietary intake	Lung cancer	2 PC	132,359 (NR)	
Gu (2022)	MA	Individuals aged 27-93 years	Dietary intake/ serum	Urothelial carcinoma	10 PC<br>9 CC	475,116 (4,310)<br>14,944 (7,140)	US, Europe, Taiwan, Australia, Uruguay
Kumar (2022)	MA	Individuals aged 25-84 years	Serum	PDAC	3 PC	NR (797)	US, Finland, "multiple countries"
Lei (2022)	MA	Individuals aged 41.4-58.4 years with disease duration ranging 1-21 years	Supplement vs other therapies	GPC relief	9 RCT	814 (482)	China
			Supplement vs other therapies	Gastric mucosa atrophy reversal	5 RCT	503 (191)	
			Supplement vs other therapies	Intestinal metaplasia reversal	2 RCT	186 (89)	
Carroll (2022)	SR	Individuals aged 19-55 years	Dietary intake (dietary habits from 1-2 years before diagnosis)	Colorectal cancer	2 CC	68,254 (NR)	Italy, US, Canada, Australia, Asia, Europe
Fu (2023)	MA	Individuals aged 25-93 years	Supplement (FU 3-36 years)	Colorectal cancer	24 PC	6,165,894 (37,280)	US, Sweden, Japan, Canada, Netherlands, Denmark, Australia

CC: case-control study; CCoh: case-cohort study; EAC: esophageal adenocarcinoma; EC: esophageal carcinoma; ESCC: esophageal squamous cell carcinoma; FU: follow-up; GPC: gastric precancerous condition; MA: meta-analysis; NCC: nested case-control study; NR: not reported; PC: prospective cohort; PDAC: pancreatic ductal adenocarcinoma; RCT: randomized controlled trial; SR: systematic review

* In the syntheses of observational studies, risk estimates were reported comparing high intake/status against low intake/status. In the syntheses of randomized controlled trials, risk estimates were reported comparing folic acid supplement against none/placebo intervention unless otherwise indicated.

Table S3. Summary of meta-analyses reporting associations between folate intake/status and cancer risks

Table S3-1. Meta-analyses reporting the association between folate intake/status and risk of breast cancer

Author (year)	Outcome	Study population/ subgroup	No. studies (design)	No. total (case)	Summary effect	I^2	P Egger
**Breast cancer – Dietary intake**							
Chen (2014)	Breast cancer	Pre/postmenopausal	15 (PC, NCC)	634,304 (21,830)	RR=0.95 (0.87, 1.03)	66.2%	0.02
			25 (CC)	39,075 (17,784)	RR=0.79 (0.67, 0.92)	82.3%	0.006
			13 (PBCC)	NR (NR)	RR=0.86 (0.71, 1.05)	83.8% (p<0.001)	0.008
			12 (HBCC)	NR (NR)	RR=0.67 (0.54, 0.85)	70% (p<0.001)	0.53
		Postmenopausal	9 (PC, NCC)	332,630 (12,174)	RR=0.94 (0.81, 1.08)	69.3% (p=0.001)	0.26
			9 (CC)	NR (NR)	RR=0.73 (0.58, 0.92)	66.2% (p=0.003)	0.01
		Premenopausal	3 (PC, NCC)	NR (NR)	RR=1.02 (0.62, 1.67)	74% (p=0.02)	0.93
			8 (CC)	NR (NR)	RR=0.78 (0.53, 1.14)	79.8% (p<0.001)	0.55
		ER+	5 (PC, NCC)	NR (NR)	RR=1.07 (0.98, 1.17)	0% (p=0.48)	0.21
		ER-	6 (PC, NCC)	NR (NR)	RR=0.95 (0.82, 1.09)	0% (p=0.93)	0.54
		North America	10 (PC, NCC)	NR (NR)	RR=0.98 (0.90, 1.08)	53.3% (p=0.02)	0.003
			8 (CC)	NR (NR)	RR=0.91 (0.76, 1.09)	68.5% (p<0.001)	<0.001
		Adjusted for alcohol use	12 (PC, NCC)	NR (NR)	RR=0.95 (0.86, 1.06)	71.1% (p<0.001)	0.06
			8 (CC)	NR (NR)	RR=0.84 (0.66, 1.06)	72.3% (p<0.001)	0.05
		Unadjusted for alcohol use	3 (PC, NCC)	NR (NR)	RR=1.10 (0.90, 1.34)	14.6% (p=0.31)	0.20
			17 (CC)	NR (NR)	RR=0.74 (0.60, 0.90)	85.4% (p<0.001)	0.02
Liu (2015)	Breast cancer	Pre/postmenopausal	16 (PC)	1,836,566 (24,083)	RR=0.98 (0.90, 1.05)	53.8% (p=0.007)	0.57
Tio (2014)	Breast cancer	Postmenopausal	9 (PC)	349,285 (13,096)	OR=0.93 (0.81, 1.07)	68.4% (p<0.01)	0.24
			11 (CC)	14,486 (5,545)	OR-0.76 (0.66, 0.86)	20.9% (p=0.24)	0.14
			9 PC, 11 CC	360,634 (15,484)	**OR=0.84 (0.75, 0.94)**	62.6% (p<0.01)	<0.01
		Premenopausal	3 (PC)	172,314 (3,123)	OR=1.02 (0.62, 1.68)	73.8% (p=0.02)	0.93
			10 (CC)	16,047 (5,941)	OR=0.75 (0.61, 0.93)	58.8% (p=0.01)	0.67
			3 PC, 10 CC	181,199 (6,376)	OR=0.81 (0.66, 1.00)	69.0% (p<0.01)	0.99
		Pre/postmenopausal	14 PC, 22 CC	608,265 (34,602)	**OR=0.84 (0.77, 0.91)**	71.2% (p<0.01)	<0.01
		ER+	4 PC, 2 CC	NR (NR)	OR=0.91 (0.77, 1.08)	67.8% (p<0.01)	0.08
		ER-	6 PC, 3 CC	NR (NR)	OR=0.96 (0.83, 1.11)	9.0% (p=0.36)	0.45
		PR+	3 PC, 1 CC	NR (NR)	OR=0.81 (0.53, 1.24)	86.2% (p<0.01)	0.42
		PR-	4 PC, 2 CC	NR (NR)	OR=1.01 (0.90, 1.13)	0% (p=0.80)	0.38
		ER+ PR+	4 PC, 2 CC	NR (NR)	OR=0.92 (0.69, 1.22)	78.8% (p<0.01)	0.46
		ER+ PR-	2 PC, 2 CC	NR (NR)	OR=0.89 (0.72, 1.10)	0% (p=0.68)	0.97
		ER- PR+	1 PC, 1 CC	NR (NR)	OR=0.49 (0.17, 1.42)	55.9% (p=0.13)	NA
		ER- PR-	4 PC, 2 CC	NR (NR)	OR=0.97 (0.81, 1.17)	0% (p=0.82)	0.21
Zeng (2019)	Breast cancer	Pre/postmenopausal	19 (PC)	NR (NR)	RR=0.95 (0.89, 1.02)	59.1% (p=0.001)	NS
Chen (2021)	Breast cancer	China	1 PC, 4 CC	13,287,305 (>2,590)	**OR=0.63 (0.46, 0.85)**	78.2%	NR
Li (2015)	Breast cancer mortality	Women with breast cancer	2 (PC)	4,624 (505)	HR=0.79 (0.61, 1.01)	0% (p=0.90)	NA

	All-cause mortality	Women with breast cancer	5 (PC)	7,299 (1,604)	**HR=0.74 (0.60, 0.92)**	35.7% (p=0.18)	0.40
**Breast cancer – Supplement**							
Chen (2014)	Breast cancer		3 (PC)	NR (NR)	RR=1.07 (0.95, 1.21)	21.7%	0.02
Zeng (2019)	Breast cancer		4 (PC)	NR (NR)	RR=1.19 (0.94, 1.18)	48.3% (p=0.12)	
**Breast cancer – Total intake**							
Chen (2014)	Breast cancer	Pre/postmenopausal	11 (PC)	187,006 (38,034)	RR=0.97 (0.87, 1.08)	66.5%	0.38
			3 (CC)	54,305 (24,151)	RR=0.87 (0.61, 1.23)	67.4%	0.41
Zeng (2019)	Breast cancer	Pre/postmenopausal	10 (PC)	NR (NR)	RR=0.98 (0.88, 1.08)	57.4% (p=0.01)	NS
Tio (2014)	Breast cancer	Postmenopausal women	7 (PC)	293,425 (10,165)	OR=0.97 (0.84, 1.12)	67.5% (p=0.01)	NR
		Premenopausal women	1 PC, 1 CC	92,682 (1,600)	OR=1.1 (0.92, 1.31)	0% (p=0.93)	NA
		Pre/postmenopausal women	15 (PC, CC)	521,474 (21,001)	OR=0.98 (0.91, 1.07)	52.7% (p=0.01)	0.44
		ER+	4 PC, 1 CC	NR (NR)	OR=1.00 (0.97, 1.04)	0% (p=0.69)	0.10
		ER-	5 PC, 1 CC	NR (NR)	OR=0.93 (0.82, 1.05)	60.5% (p=0.02)	0.06
		PR+	3 (PC)	NR (NR)	OR=1.01 (0.97, 1.04)	0% (p=0.69)	0.32
		PR-	3 (PC)	NR (NR)	OR=1.00 (0.94, 1.05)	0% (p=0.57)	0.24
		ER+ PR-	2 (PC)	NR (NR)	OR=0.83 (0.68, 1.02)	0% (p=0.71)	NA
Ren (2020)	Breast cancer	Pre/postmenopausal	19 (PC)	1,405,320 (37,917)	OR=0.97 (0.91, 1.03)	53.3% (p=0.32)	NR
			20 (CC)	30,700 (13,203)	OR=0.68 (0.57, 0.81)	76.3% (p<0.001)	
			19 PC, 20 CC	1,436,020 (51,120)	OR=0.85 (0.79, 0.92)	75.2% (p<0.001)	
Li (2015)	All-cause mortality	Women with breast cancer	4 (PC)	4,183 (752)	HR=0.93 (0.75, 1.15)	0% (p=0.96)	0.71
**Breast cancer – Plasma/serum**							
Tio (2014)	Breast cancer	Pre/postmenopausal	5 PC, 2 CC	5,226 (2,403)	OR=0.86 (0.60, 1.25)	70.3% (p<0.01)	0.16
		ER+	2 (NCC)	2,100 (NR)	**OR=1.59 (1.19, 2.12)**	0% (p=0.45)	
		ER-	2 (NCC)	2,100 (NR)	OR=1.02 (0.52, 2.00)	0% (p=0.48)	
**Breast cancer - Plasma**							
Zeng (2019)	Breast cancer		4 (PC)	NR (NR)	RR=1.01 (0.87, 1.18)	69% (p=0.02)	
Ren (2020)	Breast cancer		2 (PC)	979 (815)	OR=1.63 (0.61, 4.37)	67.9% (p=0.33)	NR
			10 (CC)	16,748 (7,850)	OR=0.93 (0.77, 1.13)	63.4% (p=0.49)	
			2 PC, 10 CC	17,727 (8,665)	OR=0.98 (0.82, 1.17)	63.0% (p=0.82)	
**Breast cancer - Serum**							
Chen (2014)	Breast cancer		5 (PC, NCC)	5,017 (2,087)	RR=1.04 (0.76, 1.42)	58%	0.84
			3 (CC)	906 (399)	OR=0.59 (0.26, 1.31)	78%	0.12
Zhang (2015)	Breast cancer		5 (CC)	2,199 (1,076)	MD= -1.04 (-2.12, 0.05)	NR	NR
**Breast cancer – Diet/Supplement**							

Zeng (2019)	Breast cancer		23 (PC)	1,171,048 (41,516)	RR=0.94 (0.88, 1.00)	Not significant	
**Breast cancer – Dose response analysis**							
Chen (2014)	Breast cancer		NR	NR	RR=1.00 (0.98, 1.01) per 100 µg/d dietary intake	NR	0.23
	Breast cancer		NR	NR	RR=1.00 (0.995, 1.013) per 100 µg/d total intake	NR	0.38
Zeng (2019)	Breast cancer	ER-	NR	NR	**RR=0.94 (0.88, 0.99) per 100 µg/d any intake**	NR	NR
	Breast cancer	ER- PR-	NR	NR	**RR=0.90 (0.85, 0.97) per 100 µg/d any intake**	NR	NR
Ren (2020)	Breast cancer		15 PC, 13 CC	NR	OR=0.98 (0.97, 0.99) per 100 µg/d total intake	NR	NR
			15 PC	NR	OR=0.99 (0.98, 1.00) per 100 µg/d total intake		
			13 CC	NR	OR=0.95 (0.92, 0.98) per 100 µg/d total intake		
Ren (2020)	Breast cancer		2 PC, 7 CC	NR	OR=0.99 (0.94, 1.04) per 5 ng/ml plasma folate	NR	NR
			2 PC	NR	OR=1.10 (0.90, 1.36) per 5 ng/ml plasma folate	NR	NR
			7 CC	NR	OR=0.96 (0.90, 1.03) per 5 ng/ml plasma folate	NR	NR

Table S3-2. Meta-analyses reporting the association between folate intake/status and risk of colorectal cancer

Author (year)	Outcome	Setting	No. studies (design)	No. total (case) Comparator	Summary effect ()	I^2	P Egger
**Colorectal cancer – Dietary intake**							
Sanjoaquin (2005)	Colorectal cancer		8 (PC)	NR (2,394)	RR=0.75 (0.64, 0.89)	P=0.67	NR
			10 (CC)	15,842 (6,166)	RR=0.76 (0.60, 0.96)	P=0.01	NR
Kennedy (2011)	Colorectal cancer	General population	11 (PC)	NR (NR)	HR=0.92 (0.81, 1.05)	42% (p=0.07)	No
		Women	7 (PC)	NR (NR)	HR=0.93 (0.80, 1.08)	31% (p=0.19)	Some
		General population	17 (CC)	3,276 (NR)	OR=0.87 (0.74, 1.02)	63% (p=0.0002)	
		Women	5 (CC)	691 (NR)	OR=0.74 (0.55, 1.01)	59% (p=0.05)	
Shiao (2018)	Colorectal cancer	General population	8 (PC)	140,771 (4,480)	SMD= 0.36 mcg/d (-0.2, 0.92)	99.5% (p<0.0001)	NR
		General population	12 (CC)	17,524 (7,312)	SMD= -0.07 mcg/d (-0.14, 0.0)	76.6% (p<0.0001)	
Fu (2023)	Colorectal cancer	General population	10 (PC)	NR (NR)	RR=0.89 (0.84, 0.95)	0% (p=0.61)	
**Colorectal cancer - Supplement**							
Burr (2017)	Colorectal cancer	Individuals with IBD	1 PC, 4 CC	3,607 (420)	**HR=0.62 (0.41, 0.83)**		
Shiao (2018)	Colorectal cancer	General population	3 (CC)	3,582 (1,637)	SMD= -2.07 mcg/d (-4.51, 0.36)	99.9% (p<0.0001)	NR
Qin (2015)	Colorectal cancer	General population	8 (RCT)	34,598 (381)	RR=1.00 (0.82, 1.22)	82.1% (p<0.001)	0.33
		Individuals with prior colorectal adenoma	3 (RCT)	2,546 (31)	RR=0.81 (0.40, 1.62)	p<0.001	
		Individuals with CVD	4 (RCT)	31,192 (346)	RR=1.02 (0.83, 1.26)	p<0.001	
		Individuals of European descent	4 (RCT)	19,031 (241)	RR=0.91 (0.71, 1.17)	p<0.001	
Liu Y (2015)	Colorectal cancer	General population	23 (PC)	1,988,974 (22,962)	**RR=0.88 (0.81, 0.95)**	42.9%	0.08
Moazzen (2018)	Colorectal cancer	General population	3 (PC)	1,926,520 (10,514)	RR=0.96 (0.76, 1.21)	0% (p=0.88)	
			4 (RCT)	31,192 (346)	RR=1.07 (0.86, 1.43)	NR	
Fu (2023)	Colorectal cancer		6 (PC)	NR (NR)	RR=0.86 (0.80, 0.92)	0% (p=0.53)	NR
**Colorectal cancer – Total intake**							
Sanjoaquin (2005)	Colorectal cancer		5 (PC)	NR (2,689)	RR=0.95 (0.81, 1.11)	P=0.33	NR
			4 (CC)	2,467 (968)	RR=0.81 (0.62, 1.05)	P=0.50	NR
Kennedy (2011)	Colorectal cancer	All	10 (CC)	1,679 (NR)	OR=0.85 (0.74, 0.99)	11% (p=0.34)	
		Men	5 (CC)	NR (NR)	OR=0.89 (0.66, 1.19)	65% (p=0.02)	
Heine-Broring (2015)	Colorectal cancer		3 (PC)	291,006 (4,066)	RR=0.88 (0.78, 0.98)	6.2% (p=0.34)	
Fu (2023)	Colorectal cancer	All	24 (PC)	6,165,894 (37,280)	**RR=0.88 (0.83, 0.92)**	34.3% (p=0.05)	0.27
		Men	6 (PC)	NR (NR)	**RR=0.76 (0.69, 0.84)**	47.6% (p=0.09)	
		Women	6 (PC)	NR (NR)	**RR=0.89 (0.81, 0.98)**	0% (p=0.48)	
		Individuals with high alcohol use	7 (PC)	NR (NR)	**RR=0.95 (0.92, 0.98)**	55.0% (p=0.04)	

		Individuals with medium alcohol use	8 (PC)	NR (NR)	**RR=0.97 (0.96, 0.99)**	62.8% (p=0.01)	
		Individuals with no/low alcohol use	6 (PC)	NR (NR)	RR=1.00 (0.98, 1.02)	0% (p=0.45)	
		US	17 (PC)	5,803,272 (32,786)	**RR=0.86 (0.82, 0.90)**	21.7% (p=0.20)	
		Europe	3 (PC)	NR (NR)	**RR=0.79 (0.64, 0.96)**	0% (p=0.84)	
**Colorectal cancer – Serum folate**							
Zhang (2015)	Colorectal cancer		11 (CC)	3,139 (1,181)	**MD= -1.10 (-1.60, -0.60)**		
**Colorectal cancer – Plasma folate**							
Shiao (2018)	Colorectal cancer		9 (PC)	8,764 (3,515)	SMD= 0.01 nmol/L (-0.07, 0.08)	47.5% (p=0.06)	NR
			8 (CC)	3,393 (1,466)	SMD= -0.46 nmol/L (-0.98, 0.0)	97.3% (p<0.0001)	
**Colorectal adenoma – Dietary intake**							
Bailie (2017)	Hyperplastic polyp		2 PC, 1 CC	33,332 (1,056)	**RR=0.65 (0.49, 0.85)**	35% (p=0.21)	NR
Shiao (2018)	Adenoma polyp		2 (PC)	1,933 (966)	SMD=0.02 mcg/d (-0.07, 0.11)	20.2% (p=0.26)	NR
			7 (CC)	4,622 (2,142)	SMD=0.0 mcg/d (-0.2, 0.2)	88% (p<0.0001)	
**Colorectal dysplasia - Supplement**							
Burr (2017)	Any dysplasia	Individuals with IBD	8 (NR)	NR (NR)	**HR=0.63 (0.31, 0.94)**	35.7% (p=0.14)	
**Colorectal adenoma – Supplement**							
Cooper (2010)	Advanced adenoma	Individuals with history of adenoma FA (0.5-1.0 mg/d for 3 years) with aspirin *	2 (RCT)	1,870 (203)	RR=1.13 (0.84, 1.51)	21% (p=0.26)	NA
		Individuals with history of adenoma FA (0.5-1.0 mg/d for 3 years) without aspirin **	2 (RCT)	749 (104)	RR=1.34 (0.77, 2.36)	55% (p=0.14)	NA
Fife (2011)	Advanced adenoma	Individuals at the risk of adenoma	2 (RCT)	1,840 (203)	OR=1.14 (0.85, 1.53)	20% (p=0.26)	
	All adenoma		2 (RCT)	3,686 (445)	OR=1.09 (0.93, 1.29)	0% (p=0.70)	NR
Vollset (2013)	All adenoma	General population	3 (RCT)	2,652 (168)	RR=1.33 (0.98, 1.80)	P=0.29	NA
Moazzen (2018)	All adenoma	General population	10 (RCT)	5,896 (236)	RR=1.00 (0.86, 1.51)	NR	
**Colorectal adenoma – RBC folate**							
Sun (2018)	Colorectal polyp/ adenomatous polyp/ hyperplastic polyp	Greece, Ireland, Australia, US	9 (CC)	2,058 (790)	SMD= 0.28 (-0.60, 1.15)	98.1% (p<0.01)	0.71
		Australia only	4 (CC)	654 (131)	SMD= 0.87 (-1.89, 3.63)		
**Colorectal adenoma – Serum folate**							
Sun (2018)	Colorectal polyp/ adenomatous polyp/ hyperplastic polyp	Greece, Australia, China, US	6 (CC)	864 (241)	SMD=0.04 (-0.27, 0.36)	73.8% (p=0.002)	0.83
		China only	3 (CC)	492 (118)	SMD=-0.02 (-0.37, 0.33)		
**Colorectal adenoma – Plasma folate**							

Shiao (2018)	Adenoma polyp		2 (PC)	885 (449)	SMD= -0.03 nmol/L (-0.16, 0.1)	0% (p=0.64)	NR
			6 (CC)	1,813 (782)	SMD= -0.05 nmol/L (-0.74, 0.65)	96.5% (p<0.0001)	
**Colorectal cancer/adenoma – Plasma folate**							
Shiao (2018)	Colorectal cancer or adenoma polyp		11 PC, 14 CC	14,951 (6,212)	SMD= -0.08 nmol/L (-0.23, 0.08)	94.6% (p<0.0001)	NR
**Colorectal cancer/ dysplasia - Supplement**							
Burr (2017)	CRC and any dysplasia	Individuals with IBD	4 PC, 6 CC	4,517 (638)	**HR=0.58 (0.37, 0.80)**	29.7% (p=0.17)	0.83
		Matched/adjusted	4 CC	671 (288)	HR=0.80 (0.52, 1.08)		
		Not matched	4 PC, 2 CC	3,846 (NR)	HR=0.54 (0.28, 0.79)	46.7% (p=0.10)	
		Before folate fortification	2 (NR)	NR (NR)	**HR=0.47 (0.20, 0.75)**		
		After folate fortification	5 (NR)	NR (NR)	HR=0.66 (0.32, 1.00)	63.8% (p=0.03)	
		NOS ≥ 7	4 PC, 4 CC	NR (NR)	**HR=0.47 (0.26, 0.67)**	18.6% (p=0.29)	
		NOS <7	2 CC	927 (347)	HR=0.83 (0.56, 1.10)		
		US	7	NR (NR)	**HR=0.58 (0.36, 0.81)**		
		Europe	3	2,675 (201)	HR=0.84 (0.41, 1.26)	47.7% (p=0.87)	
**Colorectal adenoma recurrence - Supplement**							
Davies (2006)	Colorectal preinvasive lesion recurrence		2 (RCT)	77 (22)	OR=0.58 (0.19, 1.81)		
Cooper (2010)	Colorectal adenoma recurrence	Individuals with history of adenoma FA (0.5-1.0 mg/d for 3 years) with aspirin *	3 (RCT)	1,870 (647)	RR=1.05 (0.93, 1.18)	0% (p=0.85)	
		Individuals with history of adenoma FA (0.5 – 1.0 mg/d for 3 years) without aspirin **	2 (RCT)	749 (278)	RR=1.16 (0.97, 1.39)	0% (p=0.66)	
Ibrahim (2010)	Colorectal adenoma recurrence		4 (RCT)	1,468 (564)	OR=1.08 (0.87, 1.33)	0% (p=0.53)	No
Van Dijk (2016)	Colorectal adenoma recurrence	Colorectal cancer survivors in remission or individuals with history of adenomas (0.5-5 mg/d for 3-8 years)	4 (RCT)	1,615 (444)	RR=0.93 (0.79, 1.25)	71% (p=0.02)	NR
**Colon cancer – Dietary intake**							
Kennedy (2011)	Colon cancer		4 (PC)	NR (NR)	**OR=0.75 (0.57, 0.99)**	53% (p=0.09)	
			5 (CC)	NR (NR)	OR=1.03 (0.88, 1.20)	0% (p=0.58)	
**Colon cancer – Total intake**							
Fu (2023)	Colon cancer		8 (PC)	NR (NR)	**RR=0.86 (0.81, 0.92)**	48.9% (p=0.06)	
**Rectal cancer – Dietary intake**							
Kennedy (2011)	Rectal cancer		5 (CC)	NR (NR)	OR=0.89 (0.64, 1.25)	0% (p=0.50)	
**Rectal cancer – Total intake**							
Fu (2023)	Rectal cancer		8 (PC)	NR (NR)	RR=0.92 (0.84, 1.02)	10.7% (p=0.35)	

**Colorectal cancer Dose-response analysis**						
Heine-Broring (2015)			NR	NR (NR)	RR=0.98 (0.97, 1.00) per 100 mg/d of supplement intake	0% (p=0.71)

* compared against aspirin; ** compared against placebo

Table S3-3. Meta-analyses reporting the association between folate intake/status and risk of esophageal cancer

Author (year)	Outcome	Setting	No. studies (design)	No. total (case)	Summary effect ()	I^2	P Egger
**Esophageal adenocarcinoma (EAC) – Dietary intake**							
Larsson (2006)	EAC		7 (CC)	1,769 (501)	RR=0.50 (0.39, 0.65)	0% (p=0.74)	0.33
Qiang (2018)	EAC		1 PC, 5 CC	495,407 (1,863)	**OR=0.60 (0.51, 0.69)**	34.1% (p=0.18)	NR
**Esophageal squamous cell carcinoma (ESCC) – Dietary intake**							
Larsson (2006)	ESCC		4 (CC)	3,408 (929)	RR=0.66 (0.53, 0.83)	0% (p=0.85)	0.36
Sun (2020)	ESCC	China	2 (PC)	287 (NR)	**HR=0.41 (0.25, 0.69)**	0% (p=0.79)	1.00
Qiang (2018)	ESCC		1 PC, 8 CC	497,653 (1,759)	**OR=0.61 (0.51, 0.73)**	28.2% (p=0.19)	NR
**Esophageal squamous cell carcinoma (ESCC) – Serum folate**							
Zhao (2017)	ESCC		1 NCC, 1 CC	467 (232)	**OR=0.44 (0.32, 0.61)**	NA	NA
**Esophageal cancer – Dietary intake**							
Larsson (2006)	Esophageal carcinoma	Uruguay	7 (CC)	5,177 (1,430)	**RR=0.62 (0.53, 0.72)**	0%	0.33
Zhao (2017)	Esophageal cancer		1 PC, 14 CC	4,478,995 (1,697)	OR=0.63 (0.56, 0.71)	NR	NR
		Europe	6 (CC)	1,194 (402)	OR=0.68 (0.53, 0.87)	88.2% (p=0.00)	NR
		Asia	3 (CC)	804 (234)	OR=0.61 (0.35, 1.05)	0% (p=0.55)	NR
		Americas	7 (PC)	4,476,518 (928)	OR=1.07 (0.59, 1.94)	37.8% (p=0.14)	NR
		Australia	2 (CC)	921 (173)	OR=0.74 (0.58, 0.95)	0% (p=0.75)	
		NOS ≥ 7	1 PC, 9 CC	4,477,445 (1,069)	**OR=0.60 (0.53, 0.69)**	49.2% (p=0.04)	
		NOS < 7	7 (CC)	1,818 (576)	**OR=0.69 (0.55, 0.86)**	81.7% (p=0.00)	
Ni (2017)	Esophageal cancer		2 PC, 16 CC	NR (3,892)	OR=0.64 (0.53, 0.76)	57.9% (p=0.001)	0.09
Liu (2017)	Esophageal cancer		1 PC, 12 CC	525,745 (3,743)	**OR=0.55 (0.43, 0.67)**	61.7% (p=0.001)	0.01
Qiang (2018)	Esophageal cancer		2 (PC)	491,353 (759)	**OR=0.51 (0.28, 0.94)**	73.0% (p=0.05)	<0.05
			9 (HBCC)	9,579 (2,354)	OR=0.59 (0.44, 0.77)	55.3% (p=0.02)	
			10 (PBCC)	5,809 (2,045)	OR=0.61 (0.52, 0.70)	22.8% (p=0.23)	
		Asia	3 (CC)	1,864 (679)	OR=0.77 (0.59, 1.01)	0% (p=0.58)	
		Americas	10 (CC)	498,551 (2,159)	**OR=0.58 (0.51, 0.67)**	37.3% (p=0.11)	
		Europe	6 (CC)	4,668 (1,482)	**OR=0.51 (0.40, 0.65)**	49.8% (p=0.08)	
		Australia	2 (CC)	2,345 (838)	**OR=0.74 (0.58, 0.95)**	0% (p=0.76)	
**Esophageal cancer – Total intake**							
Liu (2017)	Esophageal cancer		1 PC, 2 CC	493,761 (1,056)	**OR=0.69 (0.53, 0.85)**	0% (p=0.57)	0.41
**Esophageal cancer – Serum folate**							
Ni (2017)	Esophageal cancer		5 (CC)	2,044 (700)	OR=0.45 (0.19, 1.07)	88.7% (p=0.000)	NR
Liu (2017)	Esophageal cancer		1 PC, 4 CC	31,901 (1,100)	OR=0.71 (0.33, 1.09)	87.7% (p<0.001)	0.46
Zhang (2015)	Esophageal cancer		4 (CC)	276 (107)	MD= -6.04 (-8.63, -3.46)	NR	NR
**Esophageal cancer – Plasma folate**							
Zhao (2017)	Esophageal cancer		5 (CC)	919 (333)	**OR=0.71 (0.55, 0.92)**	9.3% (p=0.00)	0.95
		Europe	2 (NCC)	529 (152)	OR=0.13 (0.77, 2.28)	0% (p=0.38)	
		China	3 (CC)	438 (187)	**OR=0.52 (0.44, 0.79)**	92.5% (p=0.00)	
**Barrett's esophagus – Dietary intake**							

Zhao (2021)	Barrett's esophagus		2 (CC)	1,404 (371)	**RR=0.47 (0.31, 0.71)**	0% (p=0.54)	NA
**Esophageal cancer – Dose response analysis**							
Qiang (2018)	Esophageal cancer		13 (NR)	NR (NR)	OR=0.88 (0.86, 0.91) per 100 µg/d increase in dietary folate intake		
Liu (2017)	Esophageal cancer		5 (CC)	25,335 (1,209)	OR=0.91 (0.88, 0.94) per 100 µg/d increase in dietary folate intake.<br><br>Non-linearity (p<.001): The lowest risk of esophageal cancer at 405 µg/d (OR=0.69 (0.57, 0.83))		

Table S3-4. Meta-analyses reporting the association between folate intake/status and risk of gastric cancer

Author (year)	Outcome	Setting	No. studies (design)	No. total (case)	Comparator	Summary effect ()	I^2	P Egger
**Gastric cancer – Dietary intake**								
Larsson (2006)	Gastric cancer		2 (PC)	64,994 (438)		RR=1.01 (0.72, 1.42)	0% (p=0.91)	
			9 (CC)	8,341 (2,767)		RR=0.88 (0.67, 1.14)	64.7% (p=0.004)	
			2 PC, 9 CC	76,335 (3,205)		RR=0.90 (0.72, 1.13)	NR	0.28
Tio (2014)	Gastric cancer		3 (PC)	197,159 (776)		OR=1.19 (0.92, 1.54)	0% (p=0.70)	
			8 (CC)	12,530 (3,638)		OR=0.87 (0.70, 1.09)	56.2% (p=0.01)	
			3 PC, 8 CC	209,689 (4,414)		OR=0.94 (0.78, 1.14)	55.1% (p=0.003)	0.31
Liu (2017)	Gastric cancer		4 PC, 13 CC	826,498 (6,026)		**OR=0.71 (0.59, 0.84)**	71.8% (p<0.001)	0.22
**Gastric cancer – Total intake**								
Liu (2017)	Gastric cancer		2 (PC)	554,820 (1,095)		OR=0.88 (0.65, 1.12)	0% (p=0.68)	0.02
**Gastric cancer – Serum folate**								
Liu (2017)	Gastric cancer		1 PC, 3 CC	31,640 (855)		OR=1.22 (0.48, 1.96)	58.6% (p=0.05)	0.85
Zhang (2015)	Gastric cancer		3 (CC)	254 (95)		MD= -4.67 (-6.66, -2.69)	NR	NR
**Gastric precancerous conditions – Supplement**								
Lei (2022)	Symptom relief	Chinese individuals aged 41.4-58.4 years	9 (RCT) ***	814 (482)		RR $_{effectiveness}$=1.21 (0.98, 1.31)	63% (p=0.006)	*relatively large*
			9 (RCT) ***	814 (115)		**RR $_{ineffectiveness}$=0.32 (0.21, 0.48)**	48% (p=0.05)	
	Reversal of gastric mucosa atrophy lesions		5 (RCT)	503 (191)		**RR=1.61 (1.07, 2.41)**	69% (p=0.01)	
	Reversal of intestinal metaplasia lesions		2 (RCT)	186 (89)		**RR=1.77 (1.32, 2.37)**	0% (p=0.37)	
**Gastric cancer – Dose response analysis**								
Liu (2017)	Gastric cancer		2 PC, 5 CC	266,299 (2,897)		OR=0.99 (0.972, 0.998) per 100 µg/d increase in dietary folate intake		
**Gastric precancerous conditions – Time dose interval analysis**								
Lei (2022)	Gastric precancerous conditions		NR (NR)			FA has significant therapeutic effect on the pathological changes of GPC at 20-30 mg/d for 3-6 months.		
			5 (RCT)			FA has better therapeutic effect on the pathological changes of gastric mucosa of GPC at 30 mg/d for 3 months.		

*** compared against various conventional therapies

Table S3-5. Meta-analyses reporting the association between folate intake/status and risk of genitourinary cancers

Author (year)	Outcome	Setting	No. studies (design)	No. total (case)	Summary effect ()	I^2	P Egger
**Endometrial cancer – Dietary intake**							
Du (2016)	Endometrial cancer		5 (PC)	259,997 (1,977)	OR=1.05 (0.90, 1.21)	19.8% (p=0.29)	NR
			9 (CC)	10,545 (4,174)	OR=0.79 (0.64, 0.99)	59% (p=0.01)	NR
			5 PC, 9 CC	270,542 (6,151)	OR=0.89 (0.76, 1.05)	59% (p=0.00)	0.37
		North America	5 PC, 6 CC	265,753 (4,408)	OR=0.92 (0.77, 1.09)	54.9% (p=0.01)	NR
**Ovarian cancer – Dietary intake**							
Li (2013)	Ovarian cancer		4 (PC)	217,309 (1,158)	RR=0.84 (0.61, 1.14)	40.8% (p=0.17)	0.47
			4 (CC)	11,600 (4,519)	OR=0.92 (0.74, 1.14)	53.3% (p=0.09)	0.83
			4 PC, 4 CC	228,909 (5,677)	RR=0.88 (0.75, 1.05)	42.6% (p=0.09)	0.72
		North America	3 PC, 1 CC	160,124 (2,881)	RR=0.88 (0.76, 1.03)	23.8% (p=0.27)	0.79
Wang (2021)	Ovarian cancer		4 PC, 6 CC	230,240 (5,885)	RR=0.90 (0.77, 1.06)	38.8% (p=0.10)	0.96
Khodavandi (2021)	Ovarian cancer		9 (PC)	NR (NR)	RR=0.99 (0.85, 1.15)	54.0% (p=0.01)	NR
**Ovarian cancer – Total intake**							
Wang (2021)	Ovarian cancer		4 PC, 2 CC	240,493 (4,320)	RR=1.06 (0.89, 1.27)	42.8% (p=0.12)	0.16
**Prostate cancer – Dietary intake**							
Wang (2014)	Prostate cancer		5 (PC)	192,702 (NR)	RR=1.02 (0.95, 1.09)	0% (p=0.96)	0.69
Tio (2014)	Prostate cancer		5 (PC)	140,428 (12,898)	OR=1.00 (0.96, 1.05)	0% (p=0.88)	0.67
			6 (CC)	6,354 (2,438)	OR=0.83 (0.57, 1.20)	57.7% (p=0.04)	0.67
			5 PC, 6 CC	146,782 (15,336)	OR=0.97 (0.89, 1.06)	41.9% (p=0.07)	0.22
**Prostate cancer – Supplement**							
Wien (2012)	Prostate cancer		6 (RCT)	25,738 (632)	**RR=1.24 (1.03, 1.49)**	17% (p=0.31)	
**Prostate cancer – Total intake**							
Tio (2014)	Prostate cancer		2 (PC)	92,692 (6,729)	OR=1.00 (0.79, 1.27)	77.6% (p=0.03)	NA
			3 (CC)	1,089 (385)	OR=0.93 (0.61, 1.44)	28.5% (p=0.25)	0.94
			2 PC, 3 CC	93,781 (7,114)	OR=0.99 (0.82, 1.19)	48.2% (p=0.10)	0.41
**Prostate cancer – Serum folate**							
Wang (2014)	Prostate cancer		5 (NCC)	9,810 (NR)	**RR=1.21 (1.05, 1.39)**	0% (p=0.72)	0.18
Zhang (2015)	Prostate cancer		3 (CC)	7,290 (3,428)	MD= 0.13 (0.05, 0.21)	NR	NR
**Prostate cancer – Plasma/serum folate**							
Tio (2014)	Prostate cancer		6 PC, 1 CC	10,232 (6,122)	**OR=1.43 (1.06, 1.93)**	79.5% (p<0.01)	0.16
**Prostate cancer – Plasma/serum/RBC folate**							
Collin (2010)	Prostate cancer		6 (PC)	6,810 (1,497)	**OR=1.18 (1.00, 1.40)**	13%	NR
			6 PC, 1 CC	9,778 (2,958)	OR=1.11 (0.96, 1.28)	40%	NR
**Cervical cancer – Serum folate**							
Zhou (2016)	Cervical cancer	Women aged 18-74 years in US, Thailand, China, India	6 (CC)	2,383 (873)	**OR_{low}=1.94 (1.14, 3.31)**	81% (p<0.01)	<0.05
		US	3 (CC)	1,766 (598)	OR_{low}=1.11 (0.79, 1.56)	44.5% (p=0.17)	0.22
		Asia	3 (CC)	617 (275)	**OR_{low}=3.45 (2.06, 5.76)**	17.7% (p=0.30)	0.33

Table S3-6. Meta-analyses reporting the association between folate intake/status and risk of head and neck cancer

Author (year)	Outcome	Setting	No. studies (design)	No. total (case)	Summary effect ()	I^2	P Egger
**Head and neck cancer – Dietary intake**							
Fan (2017)	Head and neck cancer		7 (CC)	12,743 (3,462)	**OR=0.42 (0.34, 0.50)**	7.1%	0.82
**Head and neck cancer – Serum folate**							
Fan (2017)	Head and neck cancer		2 (CC)	2,149 (628)	OR=0.80 (0.56, 1.04)	18.3%	NA
**Head and neck squamous cell carcinoma – Serum folate**							
Zhang (2015)	Head and neck squamous cell carcinoma		3 (CC)	905 (335)	**MD= -3.97 (-4.62, -3.31)**	NR	NR
**Nasopharyngeal carcinoma – Dietary intake**							
Fan (2017)	Nasopharyngeal carcinoma		2 (CC)	1,992 (798)	**OR=0.47 (0.15, 0.79)**	67.5%	NA
**Laryngeal cancer – Dietary intake**							
Fan (2017)	Laryngeal cancer		3 (CC)	6,957 (1,659)	**OR=0.48 (0.34, 0.62)**	0%	0.73
**Head and neck cancer – Dose response analysis**							
Fan (2017)	Head and neck cancer		4 (CC)	7,281 (1,969)	**OR=0.96 (0.94, 0.98) per 100 µg/d increase in dietary folate intake**	NR	NR

Table S3-7. Meta-analyses reporting the association between folate intake/status and risk of hepatocellular cancer

Author (year)	Outcome	Setting	No. studies (design)	No. total (case)	Summary effect ()	I^2	P Egger
**Hepatocellular carcinoma – Serum folate**							
Zhang (2015)	Hepatocellular carcinoma		5 (CC)	376 (152)	**MD= -4.60 (-5.93, -3.26)**	NR	NR

Table S3-8. Meta-analyses reporting the association between folate intake/status and risk of lung cancer

Author (year)	Outcome	Setting	No. studies (design)	No. total (case)	Summary effect ()	I^2	P Egger
**Lung cancer – Dietary intake**							
Chen (2021)	Lung cancer	China	2 (NR)	NR (NR)	OR=0.97 (0.77, 1.23)	0%	NA
**Lung cancer – Serum folate**							
Zhang (2015)	Lung cancer		5 (CC)	3,519 (1,316)	MD= -1.91 (-3.04, -0.78)	NR	NR
Yang (2018)	Lung cancer		2 NCC, 10 CC	14,853 (6,995)	**SMD=-0.53 (-0.70, -0.35)**	89.4% (p=0.00)	0.04
		Europe	2 NCC, 2 CC	NR (NR)	**SMD=-0.23 (-0.30, -0.16)**	0% (p=0.45)	
		Asia	9 (CC)	NR (NR)	**SMD=-0.84 (-1.01, -0.67)**	0% (p=0.32)	
		Male	NR (NR)	NR (NR)	**OR= 0.82 (0.73, 0.92)**	25.9% (p=0.03)	
		Female	NR (NR)	NR (NR)	OR= 0.94 (0.84, 1.05)	NA	
		Former smoker	1 NCC, 2 CC	NR (NR)	**OR=0.70 (0.62, 0.79)**	32.8% (p=0.07)	
		Current smoker	1 NCC, 2 CC	NR (NR)	**OR=0.86 (0.75, 0.99)**	27.9% (p=0.21)	
		Never smoker	1 NCC, 4 CC	NR (NR)	OR=0.86 (0.75, 1.00)	36.7% (p=0.11)	

Table S3-9. Meta-analyses reporting the association between folate intake/status and risk of kidney cancers

Author (year)	Outcome	Setting	No. studies (design)	No. total (case)	Comparator	Summary effect ()	I^2	P Egger
**Renal cell carcinoma – Dietary intake**								
Clasen (2020)	Renal cell carcinoma		6 (NR)	135,821 (3,264)		RR=0.85 (0.71, 1.01)	2.1 (0 – 49.1)	
**Renal cell carcinoma – Serum folate**								
Zhang (2015)	Renal cell carcinoma		3 (CC)	1,013 (391)		**MD= -0.95 (-1.90, -0.003)**	NR	NR
**Renal cell carcinoma – Plasma/serum folate**								
Clasen (2020)	Renal cell carcinoma		2 (NR)	NR (780)		RR=0.79 (0.54, 1.15)	1.4 (0 - 56.4)	
Mao (2015)	Renal cell carcinoma		2 PC, 1 NCC, 1 CCoh, 2 CC	133,707 (2,297)		RR=0.87 (0.72, 1.05)	NR	NR
**Kidney cancer – Dietary intake**								
Clasen (2020)	Kidney cancer		8 (NR)	139,728 (4,145)		RR=0.83 (0.70, 0.998)	NR	

Table S3-10. Meta-analyses reporting the association between folate intake/status and risk of pancreatic cancer

Author (year)	Outcome	Setting	No. studies (design)	No. total (case)	Summary effect ()	I^2	P Egger
**Pancreatic cancer – Dietary intake**							
Larsson (2006)	Pancreatic cancer		4 (PC)	237,153 (618)	**RR=0.52 (0.36, 0.75)**	17.1% (p=0.31)	0.28
			4 PC, 1 CC	237,510 (722)	RR=0.49 (0.35, 0.67)		
Lin (2013)	Pancreatic cancer		7 PC, 3 CC	NR (NR)	RR=0.66 (0.49, 0.88)	76.4% (p=0.00)	0.01
Liu (2017)	Pancreatic cancer		5 PC, 3 CC	475,924 (2,659)	**OR=0.67 (0.45, 0.89)**	NR	0.16
**Pancreatic cancer - Supplement**							
Lin (2013)	Pancreatic cancer		3 PC, 1 CC	NR (NR)	RR=1.08 (0.82, 1.41)	13.6% (p=0.33)	NR
**Pancreatic cancer – Total intake**							
Liu (2017)	Pancreatic cancer		2 PC, 2 CC	180,888 (1,407)	**OR=0.76 (0.56, 0.95)**	39.8% (p=0.12)	0.83
**Pancreatic cancer – Serum folate**							
Zhang (2015)	Pancreatic cancer		4 (CC)	1,018 (288)	MD= -4.46 (-9.46, 0.55)	NR	NR
Liu (2017)	Pancreatic cancer		1 PC, 2 CC	521,998 (1,011)	OR=0.76 (0.34, 1.19)	65.7% (p=0.05)	0.07
**Pancreatic cancer – Plasma/serum folate**							
Lin (2013)	Pancreatic cancer		1 PC, 2 CC	NR (NR)	RR=0.80 (0.44, 1.45)	71.5% (p=0.03)	NR
**Pancreatic ductal adenocarcinoma – Serum folate**							
Kumar (2022)	Pancreatic ductal adenocarcinoma		3 (PC)	NR (797)	RR=0.82 (0.52, 1.30)	61% (p=0.08)	NR
**Pancreatic cancer – Dose response analysis**							
Lin (2013)	Pancreatic cancer		7 PC, 2 CC	NR (NR)	RR=0.93 (0.90, 0.97) per 100 µg/d increase in dietary folate intake	81.1% (p=0.13)	
			5 (PC)	NR (NR)	RR=0.94 (0.90, 0.99) per 100 µg/d increase in dietary folate intake	86.1% (p=0.04)	
			2 (CC)	NR (NR)	RR=0.92 (0.87, 0.97) per 100 µg/d increase in dietary folate intake	56.9% (p=0.89)	
Liu (2017)	Pancreatic cancer		5 (PC)	418,612 (1,113)	**OR=0.94 (0.92, 0.97) per 100 µg/d increase in dietary folate intake**		

Table S3-11. Meta-analyses reporting the association between folate intake/status and risk of childhood cancers

Author (year)	Outcome	Setting	No. studies (design)	No. total (case)	Summary effect ()	I^2	P Egger
**Childhood brain and spinal cord tumors - Maternal dietary intake**							
Chiavarini (2018)	Childhood brain and spinal cord tumors	Before/during pregnancy	4 (CC)	2,611 (1,089)	OR=0.76 (0.53, 1.07)	55.7%	0.35
		During pregnancy	6 (CC)	NR (NR)	OR=0.69 (0.48, 1.01)	51.94% (p=0.07)	0.59
**Childhood brain and spinal cord tumors – Maternal supplement**							
Chiavarini (2018)	Childhood brain and spinal cord tumors	Before/during pregnancy	1 PC, 7 CC	694,685 (2,994)	**OR=0.77 (0.66, 0.90)**	53.2%	0.05
		Before pregnancy	7 (NR)	NR (NR)	**OR=0.64 (0.50, 0.81)**	2.3% (p=0.41)	0.78
		During pregnancy	13 (NR)	NR (NR)	OR=0.85 (0.68, 1.05)	65.5% (p=0.001)	0.33
Wan Ismail (2019)	Childhood brain tumor		6 (CC)	10,105 (2,665)	OR=1.02 (0.88, 1.19)	35% (p=0.17)	0.29
**Childhood brain and spinal cord tumors – Maternal total intake**							
Chiavarini (2018)	Childhood brain and spinal cord tumors	Before/during pregnancy	1 PC, 9 CC	695,647 (3,475)	**OR=0.77 (0.78, 0.88)**	51.2%	0.04
		Before pregnancy	9 (NR)	NR (NR)	**OR=0.71 (0.56, 0.89)**	25.86% (p=0.21)	0.63
		During pregnancy	19 (NR)	NR (NR)	**OR=0.80 (0.67, 0.97)**	62.48% (p<0.001)	0.13
**Wilms tumor – Maternal dietary intake**							
Doganis (2020)	Wilms tumor		2 CS, 1 PC, 1 CC	50,290,620 (965)	**OR=0.79 (0.69, 0.91)**	0%	NR
**Acute lymphoblastic leukemia (ALL) – Maternal supplement**							
Dessypris (2017)	ALL	Before pregnancy	3 (CC)	10,327 (3,511)	**OR=0.69 (0.50, 0.95)**	56.8% (p=0.10)	NA
		During pregnancy	3 (CC)	10,245 (3,346)	OR=0.87 (0.57, 1.34)	89.3% (p=0.00)	NA
Wan Ismail (2019)	ALL		11 (CC)	18,405 (6,570)	**OR=0.75 (0.66, 0.86)**	62% (p=0.003)	0.13
**Acute lymphoblastic leukemia (ALL) – Child supplement**							
Dessypris (2017)	ALL		3 (CC)	1,906 (540)	OR=0.79 (0.53, 1.18)	0% (p=0.66)	NA
**Acute myeloid leukemia (AML) – Maternal supplement**							
Wan Ismail (2019)	AML		5 (CC)	5,627 (577)	OR=0.70 (0.46, 1.06)	55% (p=0.06)	0.33
**CNS and miscellaneous intracranial and intraspinal neoplasms – Maternal total intake**							
Chiavarini (2018)	CNS and miscellaneous intracranial and intraspinal neoplasms		1 PC, 15 CC	NR (NR)	**OR=0.82 (0.68, 0.99)**	62.6% (p<0.001)	0.21
**Intracranial and intraspinal embryonal tumors – Maternal total intake**							
Chiavarini (2018)	Intracranial and intraspinal embryonal tumors		10 CC	NR (NR)	**OR=0.70 (0.54, 0.90)**	37.8% (p=0.11)	0.53
**Astrocytoma – Maternal total intake**							
Chiavarini (2018)	Astrocytoma		1 PC, 2 CC	NR (NR)	OR=0.93 (0.63, 1.38)	0% (p=0.96)	0.99
**Low-grade glioma – Maternal total intake**							
Chiavarini (2018)	Low-grade glioma		3 CC	NR (NR)	**OR=0.55 (0.39, 0.79)**	0% (p=0.74)	0.51

Table S3-12. Meta-analyses reporting the association between folate intake/status and risk of total/system cancers

Author (year)	Outcome	Setting	No. studies (design)	No. total (case)	Summary effect ()	I^2	P Egger
**Digestive system cancer – Dietary intake**							
Chen (2021)	Digestive system cancer [a]	Chinese individuals aged 52.5-61.4 years	5 (NR)	NR (NR)	OR=0.91 (0.67, 1.22)	74.4%	NR
**Digestive system cancer – Serum folate**							
Zhang (2015)	Digestive system cancer [b]		16 (CC)	5,063 (1,823)	**MD= -2.61 (-2.98, -2.25)**	NR	NR
**Genital system cancer – Serum folate**							
Zhang (2015)	Genital system cancer [c]		10 (CC)	9,631 (4,571)	**MD= -1.65 (-2.45, -0.85)**	NR	NR
**Urinary system cancer – Serum folate**							
Zhang (2015)	Urinary system cancer [d]		5 (CC)	2,056 (724)	MD= -2.09 (-5.15, 0.96)	NR	NR
**Female reproductive system cancer – Dietary intake**							
Chen (2021)	Female reproductive system cancer [e]	China	2 (NR)	NR (NR)	**OR=0.59 (0.46, 0.75)**	0%	NA
**Respiratory system cancer – Serum folate**							
Zhang (2015)	Respiratory system cancer [f]		7 (CC)	3,744 (1,401)	**MD= -2.11 (-3.15, -1.07)**	NR	NR
**All cancers – Dietary intake**							
Chen (2021)	All cancers	China	7 (PC)	NR (NR)	OR=0.93 (0.82, 1.07)	0%	
			9 (CC)	NR (NR)	OR=0.58 (0.48, 0.72)	72.7%	
			7 PC, 9 CC	561,538 (10,073)	**OR=0.73 (0.61, 0.88)**	75.0%	0.69
**All cancers – Supplement**							
Zhou (2011)	Total cancer incidence	Individuals with cardiovascular or renal conditions	6 (RCT)	26,544 (2,472)	RR=1.08 (0.98, 1.21)	26.7% (p=0.23)	
Wien (2012)	Total cancer incidence		10 (RCT)	38,233 (3,515)	RR=1.07 (1.00, 1.14)	0% (p=0.45)	
Wien (2012)	Total cancer mortality		6 (RCT)	32,327 (1,134)	RR=1.09 (0.87, 1.22)	45% (p=0.12)	
**All cancers – Serum folate**							
Zhang (2015)	All cancers		40 (CC)	21,696 (9,047)	**MD= -2.68 (-3.21, -2.15)**	NR	0.06
		Europe	15 (CC)	10,692 (4,870)	**MD= -1.17 (-1.55, -0.79)**		
		Asia	13 (CC)	2,923 (1,157)	**MD= -4.65 (-5.82, -3.47)**		
		America	5 (CC)	3,548 (1,387)	MD= -0.24 (-0.54, 0.07)		
		Middle East	5 (CC)	1,728 (614)	**MD= -1.40 (-2.40, -0.41)**		
**All cancers – Dose response analysis**							

Chen (2021)	All cancers	China	6 PC, 6 CC	NR (NR)	**OR=0.86 (0.80, 0.92)** per 100 µg/d increase in dietary folate intake	NR	NR

a: colorectal cancer, pancreatic cancer, esophageal cancer, gastric cancer; b: colorectal cancer, pancreatic cancer, esophageal cancer, hepatocellular cancer, gastric cancer, cholangiocarcinoma; c: cervical cancer, ovarian cancer, prostate cancer, breast cancer, endometrial cancer; d: renal carcinoma, bladder cancer, urothelial carcinoma; e: endometrial cancer, ovarian cancer; f: lung cancer, laryngeal squamous cell carcinoma

ALL: acute lymphoblastic leukemia; AML: acute myeloid leukemia; CC: case-control; CS: cross-sectional; EAC: esophageal adenocarcinoma; EC: esophageal carcinoma; ESCC: esophageal squamous cell carcinoma; FA: folic acid; FU: follow-up; GAC: gastric cardiac adenocarciniioma; GPC: gastric precancerous condition; SR: systematic review; MA: meta-analysis; NCC: nested case-control; NR: not reported; NTD: neural tube defects; PC: prospective cohort; PDAC: pancreatic ductal adenocarcinoma; RCT: randomized controlled trial

Table S4. Identified associations between folate intake/status and risk of cancers and assessment of credibility

Outcome (Subtype/subgroup)	Primary study design	Exposure	Total N (Case N)	Metric	Summary estimate (95% CI)	Estimated p-value	I^2 (%)	Ref No.	Credibility
Breast cancer, premenopausal	PC, CC	Dietary folate	181,199 (6,376)	OR	**0.81 (0.66, 1.00)**	0.04681655	69	Tio 2014	Weak
Breast cancer, postmenopausal	PC, CC	Dietary folate	360,634 (15,484)	OR	**0.84 (0.75, 0.94)**	0.00247180	62.6	Tio 2014	Suggestive
Breast cancer, all	PC, CC	Dietary folate	608,265 (34,602)	OR	**0.84 (0.77, 0.91)**	0.00004290	71.2	Tio 2014	Suggestive
Breast cancer, ER+	PC, CC	Dietary folate	NR (NR)	OR	0.91 (0.77, 1.08)	0.27451352	67.8	Tio 2014	Weak
Breast cancer, ER-	PC, CC	Dietary folate	NR (NR)	OR	0.96 (0.83, 1.11)	0.58198214	9	Tio 2014	Weak (Insuff. data)
Breast cancer, PR+	PC, CC	Dietary folate	NR (NR)	OR	0.81 (0.53, 1.24)	0.33114625	86.2	Tio 2014	Weak
Breast cancer, PR-	PC, CC	Dietary folate	NR (NR)	OR	1.01 (0.90, 1.13)	0.86391477	0	Tio 2014	Weak (Insuff. data)
Breast cancer, ER+PR+	PC, CC	Dietary folate	NR (NR)	OR	0.92 (0.69, 1.22)	0.56629442	78.8	Tio 2014	Weak
Breast cancer, ER+PR-	PC, CC	Dietary folate	NR (NR)	OR	0.89 (0.72, 1.10)	0.28109610	0	Tio 2014	Weak (Insuff. data)
Breast cancer, ER-PR+	PC, CC	Dietary folate	NR (NR)	OR	0.49 (0.17, 1.42)	0.18770454	55.9	Tio 2014	Weak
Breast cancer, ER-PR-	PC, CC	Dietary folate	NR (NR)	OR	0.97 (0.81, 1.17)	0.74540843	0	Tio 2014	Weak (Insuff. data)
Breast cancer, North America	PC, NCC	Dietary folate	NR (NR)	RR	0.98 (0.90, 1.08)	0.66402138	53.3	Chen 2014	Weak
Breast cancer, China only	PC, CC	Dietary folate	13,287,305 (>2,590)	OR	**0.63 (0.46, 0.85)**	0.00318025	78.2	Chen 2021	Suggestive
Breast cancer, adjusted for alcohol use	PC, NCC	Dietary folate	NR (NR)	RR	0.95 (0.86, 1.06)	0.33623365	71.1	Chen 2014	Weak
Breast cancer mortality	PC	Dietary folate	4,624 (505)	HR	0.79 (0.61, 1.01)	0.06687709	0	Li 2015	Weak
All-cause mortality in individuals with breast cancer	PC	Dietary folate	7,299 (1,604)	HR	**0.74 (0.60, 0.92)**	0.00575591	35.7	Li 2015	Suggestive
Breast cancer	PC	Supplement	NR (NR)	RR	1.19 (0.94, 1.18)	0.00271044	48.3	Zeng 2019	Weak (Insuff. data)
Breast cancer, premenopausal	PC, CC	Total folate	92,682 (1,600)	OR	1.10 (0.92, 1.31)	0.29043042	0	Tio 2014	Suggestive
Breast cancer, postmenopausal	PC	Total folate	293,425 (10,165)	OR	0.97 (0.84, 1.12)	0.67811128	67.5	Tio 2014	Weak
Breast cancer	PC, CC	Total folate	1,436,020 (51,120)	OR	0.85 (0.75, 1.15)	0.13611193	75.20	Ren 2020	Weak
Breast cancer, ER+	PC, CC	Total folate	NR (NR)	OR	1.00 (0.97, 1.04)	1.00000000	0	Tio 2014	Weak (Insuff. data)
Breast cancer, ER-	PC, CC	Total folate	NR (NR)	OR	0.93 (0.82, 1.05)	0.24989433	60.5	Tio 2014	Weak
Breast cancer, PR+	PC	Total folate	NR (NR)	OR	1.01 (0.97, 1.04)	0.57563077	0	Tio 2014	Weak (Insuff. data)
Breast cancer, PR-	PC	Total folate	NR (NR)	OR	1.00 (0.94, 1.05)	1.00000000	0	Tio 2014	Weak (Insuff. data)
Breast cancer, ER+PR-	PC	Total folate	NR (NR)	OR	0.83 (0.68, 1.02)	0.07163710	0	Tio 2014	Weak (Insuff. data)
All-cause mortality in individuals with breast cancer	PC	Total folate	NR (NR)	HR	0.93 (0.75, 1.15)	0.50571111	0	Li 2015	Weak (Insuff. data)

Breast cancer	PC, NCC	Serum folate	4,183(752)	RR	1.04 (0.76, 1.42)	0.80571673	58	Chen 2014	Weak
Breast cancer	PC, CC	Plasma folate	17,727 (8,665)	OR	0.98 (0.82, 1.17)	0.82369268	63	Ren 2020	Weak
Breast cancer	PC, CC	Plasma/serum folate	5,226 (2,403)	OR	0.86 (0.60, 1.25)	0.42052055	70.3	Tio 2014	Weak
Breast cancer, ER+	NCC	Plasma/serum folate	NR (NR)	OR	**1.59 (1.19, 2.12)**	0.00164407	0	Tio 2014	Weak (Insuff. data)
Breast cancer, ER-	NCC	Plasma/serum folate	NR (NR)	OR	1.02 (0.52, 2.00)	0.95404664	0	Tio 2014	Weak (Insuff. data)
Colorectal cancer	PC	Dietary folate	140,771 (4,480)	SMD	0.36 (-0.20, 0.92)	0.34032995	99.5	Shiao 2018	Weak
Colorectal cancer, women	PC	Dietary folate	NR (NR)	HR	0.93 (0.80, 1.08)	0.34316684	31	Kennedy 2011	Weak (Insuff. data)
Colorectal cancer	PC	Supplement	1,988,974 (22,962)	RR	**0.88 (0.81, 0.95)**	0.00167135	42.9	Liu 2015	Suggestive
Colorectal cancer in individuals with IBD	PC, CC	Supplement	3,607 (420)	HR	**0.62 (0.41, 0.83)**	0.00788388		Burr 2017	Weak
Colorectal cancer in individuals with prior adenoma	RCT	Supplement	2,546 (31)	RR	0.81 (0.40, 1.62)	0.55481518	<0.001	Qin 2015	Weak
Colorectal cancer in individuals with CVD	RCT	Supplement	31,192 (346)	RR	1.02 (0.83, 1.26)	0.85247817	<0.001	Qin 2015	Weak
Colorectal cancer in individuals of European descent	RCT	Supplement	19,031 (241)	RR	0.91 (0.71, 1.17)	0.45921222	<0.001	Qin 2015	Weak
Colorectal cancer	PC	Total folate	6,165,894 (37,280)	RR	**0.88 (0.83, 0.92)**	0.00000113	34.3	Fu 2023	Highly suggestive
Colorectal cancer, men	PC	Total folate	NR (NR)	RR	**0.76 (0.69, 0.84)**	0.00000005	47.6	Fu 2023	Suggestive (Insuff. data)
Colorectal cancer, women	PC	Total folate	NR (NR)	RR	**0.89 (0.81, 0.98)**	0.01649677	0	Fu 2023	Weak (Insuff. data)
Colorectal cancer in individuals with high alcohol use	PC	Total folate	NR (NR)	RR	**0.95 (0.92, 0.98)**	0.00145987	55	Fu 2023	Weak (Insuff. data)
Colorectal cancer in individuals with medium alcohol use	PC	Total folate	NR (NR)	RR	**0.97 (0.96, 0.99)**	0.00010437	62.8	Fu 2023	Weak (Insuff. data)
Colorectal cancer in individuals with no/low alcohol use	PC	Total folate	NR (NR)	RR	1.00 (0.98, 1.02)	1.00000000	0	Fu 2023	Weak (Insuff. data)
Colorectal cancer, US	PC	Total folate	5,803,272 (32,786)	RR	**0.86 (0.82, 0.90)**	0.00000000	21.7	Fu 2023	Highly suggestive
Colorectal cancer, Europe	PC	Total folate	NR (NR)	RR	**0.79 (0.64, 0.96)**	0.02267050	0	Fu 2023	Weak
Colorectal cancer	CC	Serum folate	3,139 (1,181)	MD	**-1.10 (-1.60, -0.60)**	0.00001618		Zhang 2015	Suggestive
Colorectal cancer	PC	Plasma folate	8,764 (3,515)	SMD	0.01 (-0.07, 0.08)	0.79383546	47.50	Shiao 2018	Suggestive
Hyperplastic polyp	PC, CC	Dietary folate	33,332 (1,056)	RR	**0.65 (0.49, 0.85)**	0.00217179	35	Bailie 2017	Suggestive
Adenoma polyp	PC	Dietary folate	1,933 (966)	SMD	0.02 (-0.07, 0.11)	0.66315922	20.2	Shiao 2018	Weak
Colorectal dysplasia in individuals with adenoma	NR	Supplement	NR (NR)	HR	**0.63 (0.31, 0.94)**	0.10252973	35.7	Burr 2017	Data unavailable

Colorectal adenoma in at-risk individuals	RCT	Supplement	3,686 (445)	OR	1.09 (0.93, 1.29)	0.30188197	0	Fife 2011	Weak
Colorectal adenoma in general population	RCT	Supplement	5,896 (236)	RR	1.00 (0.86, 1.51)	1.00000000		Moazzen 2018	Weak
Advanced adenoma in individuals with history of adenoma, with aspirin	RCT	Supplement	1,870 (203)	RR	1.13 (0.84, 1.51)	0.41397441	21	Cooper 2010	Weak
Advanced adenoma in individuals with history of adenoma, without aspirin	RCT	Supplement	749 (104)	RR	1.34 (0.77, 2.36)	0.30568438	55	Cooper 2010	Weak
Colorectal adenoma, overall	CC	RBC folate	2,058 (790)	SMD	0.28 (-0.60, 1.15)	0.53052814	98.1	Sun 2018	Weak
Colorectal adenoma, Australia	CC	RBC folate	654 (131)	SMD	0.87 (-1.89, 3.63)	0.53668999		Sun 2018	Weak
Colorectal adenoma, overall	CC	Serum folate	864 (241)	SMD	0.04 (-0.27, 0.36)	0.80344673	73.8	Sun 2018	Weak
Colorectal adenoma, China	CC	Serum folate	492 (118)	SMD	-0.02 (-0.37, 0.33)	0.91082341		Sun 2018	Weak
Colorectal adenoma, overall	CC	Plasma folate	1,813 (782)	SMD	-0.05 (-0.74, 0.65)	0.88786426	96.5	Shiao 2018	Weak
Colorectal cancer or adenoma	PC, CC	Plasma folate	14,951 (6,212)	SMD	-0.08 (-0.23, 0.08)	0.31172317	94.6	Shiao 2018	Weak
Colorectal cancer or any dysplasia in individuals with IBD	PC, CC	Supplement	4,517 (638)	HR	**0.58 (0.37, 0.80)**	0.00561994	29.7	Burr 2017	Weak
Colorectal cancer or any dysplasia in individuals with IBD before fortification	NR	Supplement	NR (NR)	HR	**0.47 (0.20, 0.75)**	0.02514227		Burr 2017	Weak
Colorectal cancer or any dysplasia in individuals with IBD after fortification	NR	Supplement	NR (NR)	HR	0.66 (0.32, 1.00)	0.15286077	63.8	Burr 2017	Weak
Colorectal cancer or any dysplasia in individuals with IBD in the US	NR	Supplement	NR (NR)	HR	**0.58 (0.36, 0.81)**	0.00845879		Burr 2017	Weak (Insuff. data)
Colorectal cancer or any dysplasia in individuals with IBD in Europe	NR	Supplement	NR (NR)	HR	0.84 (0.41, 1.26)	0.54268091	47.7	Burr 2017	Weak (Insuff. data)
Colorectal cancer or any dysplasia in individuals with IBD in high quality studies	PC, CC	Supplement	NR (NR)	HR	**0.47 (0.26, 0.67)**	0.00176801	18.6	Burr 2017	Weak (Insuff. data)
Colorectal adenoma recurrence	RCT	Supplement	1,615 (444)	RR	0.93 (0.79, 1.25)	0.53528610	71	Van Dijk 2016	Weak
Colorectal adenoma recurrence, with aspirin	RCT	Supplement	1,870 (647)	RR	1.05 (0.93, 1.18)	0.42179248	0	Cooper 2010	Weak
Colon cancer	PC	Dietary folate	NR (NR)	OR	**0.75 (0.57, 0.99)**	0.04108157	53	Kennedy 2011	Weak
Colon cancer	PC	Total folate	NR (NR)	RR	**0.86 (0.81, 0.92)**	0.00000344		Fu 2023	Weak (Insuff. data)
Rectal cancer	CC	Dietary folate	NR (NR)	OR	0.89 (0.64, 1.25)	0.49499259		Kennedy 2011	Weak (Insuff. data)
Rectal cancer	PC	Total folate	NR (NR)	RR	0.92 (0.84, 1.02)	0.09228406		Fu 2023	Weak
EAC	PC, CC	Dietary folate	495,407 (1,863)	OR	**0.60 (0.51, 0.69)**	0.00000000	34.1	Qiang 2018	Highly suggestive (Insuff. data)
ESCC	PC, CC	Dietary folate	497,653 (1,759)	OR	**0.61 (0.51, 0.73)**	0.00000007	28.2	Qiang 2018	Highly suggestive (Insuff. data)
ESCC, China	PC	Dietary folate	287 (NR)	HR	**0.41 (0.25, 0.69)**	0.00057608	0	Sun 2020	Weak

ESCC	NCC, CC	Serum folate	467 (232)	OR	**0.44 (0.32, 0.61)**	0.00000061		Zhao 2017	Weak
Esophageal cancer, Uruguay	CC	Dietary folate	5,177 (1,430)	RR	**0.62 (0.53, 0.72)**	0.00000000	0.00	Larsson 2006	Highly suggestive (Insuff. data)
Esophageal cancer, NOS ≥ 7	PC, CC	Dietary folate	4,477,445 (1,069)	OR	**0.60 (0.53, 0.69)**	0.00000000	49.2	Zhao 2017	Highly suggestive (Insuff. data)
Esophageal cancer	PC, CC	Dietary folate	525,745 (3,743)	OR	**0.55 (0.43, 0.67)**	0.00000013	61.7	Liu 2017	Highly suggestive
Esophageal cancer, Asia	CC	Dietary folate	1,864 (679)	OR	0.77 (0.59, 1.01)	0.05667035	0	Qiang 2018	Weak
Esophageal cancer, Americas	CC	Dietary folate	498,551 (2,159)	OR	**0.58 (0.51, 0.67)**	0.00000000	37.3	Qiang 2018	Highly suggestive (Insuff. data)
Esophageal cancer, Europe	CC	Dietary folate	4,668 (1,482)	OR	**0.51 (0.40, 0.65)**	0.00000005	49.8	Qiang 2018	Highly suggestive (Insuff. data)
Esophageal cancer, Australia	CC	Dietary folate	2,345 (838)	OR	**0.74 (0.58, 0.95)**	0.01675332	0	Qiang 2018	Weak
Esophageal cancer	PC, CC	Total folate	493,761 (1,056)	OR	**0.69 (0.53, 0.85)**	0.00207438	0	Liu 2017	Suggestive
Esophageal cancer	PC, CC	Serum folate	31,901 (1,100)	OR	0.71 (0.33, 1.09)	0.26116875	87.7	Liu 2017	Weak
Esophageal cancer	CC	Plasma folate	919 (333)	OR	**0.71 (0.55, 0.92)**	0.00906280	9.3	Zhao 2017	Weak
Esophageal cancer, Europe	NCC	Plasma folate	529 (152)	OR	0.13 (0.77, 2.28)	0.00000000	0	Zhao 2017	Weak
Esophageal cancer, China	CC	Plasma folate	438 (187)	OR	**0.52 (0.44, 0.79)**	0.00001187	92.5	Zhao 2017	Weak
Barrett's esophagus	CC	Dietary folate	1,404 (371)	RR	**0.47 (0.31, 0.71)**	0.00035492	0	Qiang 2018	Weak
Gastric cancer	PC, CC	Dietary folate	826,498 (6,026)	OR	**0.71 (0.59, 0.84)**	0.00014453	71.8	Liu 2017	Suggestive
Gastric cancer	PC	Total folate	554,820 (1,095)	OR	0.88 (0.65, 1.12)	0.35706959	0	Liu 2017	Suggestive
Gastric cancer	PC, CC	Serum folate	31,640 (855)	OR	1.22 (0.48, 1.96)	0.57954713	58.6	Liu 2017	Weak
GPC symptom relief (ineffectiveness), China	RCT	Supplement	814 (115)	RR	**0.32 (0.21, 0.48)**	0.00000007	48	Lei 2022	Weak
GPC, reversal of gastric mucosa atrophy lesions, China	RCT	Supplement	503 (191)	RR	**1.61 (1.07, 2.41)**	0.02149632	69	Lei 2022	Weak
GPC, reversal of intestinal metaplasia lesions, China	RCT	Supplement	186 (89)	RR	**1.77 (1.32, 2.37)**	0.00013111	0	Lei 2022	Weak
Endometrial cancer	CC	Dietary folate	270,542 (6,151)	OR	0.89 (0.76, 1.03)	0.13291787	59	Du 2016	Weak
Endometrial cancer, North America	PC, CC	Dietary folate	265,753 (4,408)	OR	0.92 (0.77, 1.09)	0.34697262	54.9	Du 2016	Weak
Ovarian cancer	PC, CC	Dietary folate	230,240 (5,885)	RR	0.90 (0.77, 1.06)	0.19630675	38.8	Wang 2021	Suggestive
Ovarian cancer, North America	PC, CC	Dietary folate	160,124 (2,881)	RR	0.88 (0.76, 1.03)	0.09927035	23.8	Li 2013	Weak
Ovarian cancer	PC, CC	Total folate	240,493 (4,320)	RR	1.06 (0.89, 1.27)	0.52059827	42.8	Wang 2021	Suggestive
Prostate cancer	PC, CC	Dietary folate	146,782 (15,336)	OR	0.97 (0.89, 1.06)	0.49457130	41.9	Tio 2014	Suggestive
Prostate cancer	RCT	Supplement	25,738 (632)	RR	**1.24 (1.03, 1.49)**	0.02238041	17	Wien 2012	Weak
Prostate cancer	NCC	Total folate	93,781 (7,114)	OR	0.99 (0.82, 1.19)	0.91574754	48.2	Tio 2014	Suggestive
Prostate cancer	NCC	Serum folate	9,810 (NR)	RR	**1.21 (1.05, 1.39)**	0.00772637	0	Wang 2014	Weak (Insuff. data)
Prostate cancer	PC, CC	Plasma/serum folate	10,232 (6,122)	OR	**1.43 (1.06, 1.93)**	0.01929785	79.5	Tio 2014	Weak

Prostate cancer	PC, CC	Plasma/serum/ RBC folate	9,778 (2,958)	OR	1.11 (0.96, 1.28)	0.15501883	40	Collin 2010	Suggestive
Cervical cancer	CC	Serum folate	2,383 (873)	OR_{low}	**1.94 (1.13, 3.31)**	0.01564449	81	Zhou 2016	Weak
Cervical cancer, US	CC	Serum folate	1,766 (598)	OR^{low}	1.11 (0.79, 1.56)	0.54767754	44.5	Zhou 2016	Weak
Cervical cancer, Asia	CC	Serum folate	617 (275)	OR_{low}	**3.45 (2.06, 5.76)**	0.00000235	17.7	Zhou 2016	Weak
Head and neck cancer	CC	Dietary folate	12,743 (3,462)	OR	**0.42 (0.34, 0.50)**	0.00000000	7.1	Fan 2017	Highly suggestive (Insuff. data)
Head and neck cancer	CC	Serum folate	2,149 (628)	OR	0.80 (0.56, 1.04)	0.15764607	18.3	Fan 2017	Weak
Head and neck squamous cell carcinoma	CC	Serum folate	905 (335)	MD	**-3.97 (-4.62, -3.31)**	0.00000000		Zhang 2015	Weak
Nasopharyngeal carcinoma	CC	Dietary folate	1,992 (798)	OR	**0.47 (0.15, 0.79)**	0.07483978	67.5	Fan 2017	Weak
Laryngeal cancer	CC	Dietary folate	6,957 (1,659)	OR	**0.48 (0.34, 0.62)**	0.00000168	0	Fan 2017	Suggestive
Hepatocellular cancer	CC	Serum folate	376 (152)	MD	**-4.60 (-5.93, -3.26)**	0.00000000		Zhang 2015	Weak
Lung cancer, China	NR	Dietary folate	NR (NR)	OR	0.97 (0.77, 1.23)	0.79878335	0	Chen 2021	Weak (Insuff. data)
Lung cancer	NCC, CC	Serum folate	14,853 (6,995)	SMD	**-0.53 (-0.70, -0.35)**	0.00000000	89.4	Yang 2018	Suggestive
Lung cancer, Europe	NCC, CC	Serum folate	NR (NR)	SMD	**-0.23 (-0.30, -0.16)**	0.00000000	0	Yang 2018	Highly suggestive (Insuff. data)
Lung cancer, Asia	CC	Serum folate	NR (NR)	SMD	**-0.84 (-1.01, -0.67)**	0.00000000	0	Yang 2018	Highly suggestive (Insuff. data)
Lung cancer, male	NR	Serum folate	NR (NR)	OR	**0.82 (0.73, 0.92)**	0.00077139	25.9	Yang 2018	Weak (Insuff. data)
Lung cancer, female	NR	Serum folate	NR (NR)	OR	0.94 (0.84, 1.05)	0.27704762		Yang 2018	Weak (Insuff. data)
Lung cancer, former smokers	NCC, CC	Serum folate	NR (NR)	OR	**0.70 (0.62, 0.79)**	0.00000001	32.8	Yang 2018	Highly suggestive (Insuff. data)
Lung cancer, current smokers	NCC, CC	Serum folate	NR (NR)	OR	**0.86 (0.75, 0.99)**	0.03321025	27.9	Yang 2018	Weak
Lung cancer, never smokers	NCC, CC	Serum folate	NR (NR)	OR	0.86 (0.75, 1.00)	0.03986589	36.7	Yang 2018	Weak
Renal cell carcinoma	NR	Dietary folate	135,821 (3,264)	RR	0.85 (0.71, 1.01)	0.07066767		Clasen 2020	Weak
Renal cell carcinoma	CC	Serum folate	1,013 (391)	MD	-0.95 (-1.90, 0.00)	0.04963460		Zhang 2015	Weak
Renal cell carcinoma	PC, CC, CC	Serum/Plasma	133,707 (2,297)	RR	0.87 (0.72, 1.05)	0.14792472		Mao 2015	Weak
Kidney cancer	NR	Dietary folate	139,728 (4,145)	RR	0.83 (0.70, 1.00)	0.03945634		Clasen 2020	Weak
Pancreatic cancer	PC, CC	Dietary folate	475,924 (2,659)	OR	**0.67 (0.45, 0.89)**	0.02133780		Liu 2017	Weak
Pancreatic cancer	PC, CC	Supplement	NR (NR)	RR	1.08 (0.82, 1.41)	0.57781656	13.6	Lin 2013	Weak (Insuff. data)
Pancreatic cancer	PC, CC	Total folate	180,888 (1,407)	OR	**0.76 (0.56, 0.95)**	0.04180451	39.8	Liu 2017	Weak
Pancreatic cancer	PC, CC	Serum folate	521,998 (1,011)	OR	0.76 (0.34, 1.19)	0.39048628	65.7	Liu 2017	Weak
Pancreatic cancer	PC, CC	Serum/plasma	NR (NR)	RR	0.80 (0.44, 1.45)	0.46325781	71.5	Lin 2013	Weak
Pancreatic ductal adenocarcinoma	PC	Serum folate	NR (NR)	RR	0.82 (0.52, 1.30)	0.39588321	61	Kumar 2022	Weak

Childhood brain and spinal cord tumors, before/during pregnancy	CC	Maternal dietary folate	2,611 (1,089)	OR	0.76 (0.53, 1.07)	0.12569609	55.7	Chiavarini 2018	Weak
Childhood brain and spinal cord tumors, during pregnancy	CC	Maternal dietary folate	NR (NR)	OR	0.69 (0.48, 1.01)	0.05055024	51.9	Chiavarini 2018	Weak
Childhood brain and spinal cord tumors, before/during pregnancy	PC, CC	Maternal supplement	694,685 (2,994)	OR	**0.77 (0.66, 0.90)**	0.00095538	53.2	Chiavarini 2018	Suggestive
Childhood brain and spinal cord tumors, before pregnancy	NR	Maternal supplement	NR (NR)	OR	**0.64 (0.50, 0.81)**	0.00028746	2.3	Chiavarini 2018	Weak (Insuff. data)
Childhood brain and spinal cord tumors, during pregnancy	NR	Maternal supplement	NR (NR)	OR	0.85 (0.68, 1.05)	0.14254383	65.5	Chiavarini 2018	Weak
Childhood brain and spinal cord tumors, before/during pregnancy	PC, CC	Maternal total folate	695,647 (3,475)	OR	**0.77 (0.78, 0.88)**	0.00000000	51.2	Chiavarini 2018	Highly suggestive
Childhood brain and spinal cord tumors, before pregnancy	NR	Maternal total folate	NR (NR)	OR	**0.71 (0.56, 0.89)**	0.00375646	25.9	Chiavarini 2018	Weak (Insuff. data)
Childhood brain and spinal cord tumors, during pregnancy	NR	Maternal total folate	NR (NR)	OR	**0.80 (0.67, 0.97)**	0.01807890	62.5	Chiavarini 2018	Weak
Wilms tumor	CS, PC, CC	Maternal dietary folate	509,290,620 (965)	OR	**0.79 (0.69, 0.91)**	0.00084132	0	Doganis 2020	Weak
Acute lymphoblastic leukemia, before/during pregnancy	CC	Maternal supplement	18,405 (6,570)	OR	**0.75 (0.66, 0.86)**	0.00002040	62	Wan Ismail 2019	Suggestive
Acute lymphoblastic leukemia, before pregnancy	CC	Maternal supplement	10,326 (3,511)	OR	**0.69 (0.50, 0.95)**	0.02343911	56.8	Dessypris 2017	Weak
Acute lymphoblastic leukemia, during pregnancy	CC	Maternal supplement	10,245 (3,346)	OR	0.87 (0.57, 1.34)	0.52305323	89.3	Dessypris 2017	Weak
Acute lymphoblastic leukemia	CC	Child supplement	1,906 (540)	OR	0.79 (0.53, 1.18)	0.24830642	0	Dessypris 2017	Weak
Acute myeloid leukemia	CC	Maternal supplement	5,627 (577)	OR	0.70 (0.46, 1.06)	0.09396252	55	Wan Ismail 2019	Weak
CNS and miscellaneous intracranial/intraspinal neoplasms	PC, CC	Maternal total	NR (NR)	OR	**0.82 (0.68, 0.99)**	0.03835011	62.6	Chiavarini 2018	Weak
Intracranial/intracranial embryonal tumors	CC	Maternal total folate	NR (NR)	OR	**0.70 (0.54, 0.90)**	0.00619890	37.8	Chiavarini 2018	Weak (Insuff. data)
Astrocytoma	PC, CC	Maternal total folate	NR (NR)	OR	0.93 (0.63, 1.38)	0.71675548	0	Chiavarini 2018	Weak (Insuff. data)
Low-grade glioma	CC	Maternal total folate	NR (NR)	OR	**0.55 (0.39, 0.79)**	0.00090027	0	Chiavarini 2018	Weak (Insuff. data)
Digestive system cancer, China	NR	Dietary folate	NR (NR)	OR	0.91 (0.67, 1.22)	0.53733133	74.4	Chen 2021	Weak
Digestive system cancer	CC	Serum folate	5,063 (1,823)	MD	**-2.61 (-2.98, -2.25)**	0.00000000		Zhang 2015	Highly suggestive (Insuff. data)
Genital system cancer	CC	Serum folate	9,631 (4,571)	MD	**-1.65 (-2.45, -0.85)**	0.00005288		Zhang 2015	Suggestive
Urinary system cancer	CC	Serum folate	2,056 (724)	MD	-2.09 (-5.15, 0.96)	0.17995818		Zhang 2015	Weak
Female reproductive system cancer, China	NR	Dietary folate	NR (NR)	OR	**0.59 (0.46, 0.75)**	0.00002326	0	Chen 2021	Weak (Insuff. data)
Respiratory system cancer	CC	Serum folate	3,744 (1,401)	MD	**-2.11 (-3.15, -1.07)**	0.00006993		Zhang 2015	Suggestive

Total cancer, China	PC, CC	Dietary folate	561,538 (10,073)	OR	**0.73 (0.61, 0.88)**	0.00076152	75	Chen 2021	Suggestive
Total cancer	RCT	Supplement	38,233 (3,515)	RR	**1.07 (1.00, 1.14)**	0.04295390	0	Wien 2012	Weak
Total cancer, individuals with CV/renal conditions	RCT	Supplement	26,544 (2,472)	RR	1.08 (0.98, 1.21)	0.15243093	26.7	Zhou 2011	Suggestive
Total cancer mortality	RCT	Supplement	32,327 (1,134)	RR	1.09 (0.87, 1.22)	0.31773487	45	Wien 2012	Suggestive
Total cancer	CC	Serum folate	21,696 (9,047)	MD	**-2.68 (-3.21, -2.15)**	0.00000000		Zhang 2015	Highly suggestive
Total cancer, Europe	CC	Serum folate	10,692 (4,870)	MD	**-1.17 (-1.55, -0.79)**	0.00000000		Zhang 2015	Highly suggestive (Insuff. data)
Total cancer, Asia	CC	Serum folate	2,923 (1,157)	MD	**-4.65 (-5.82, -3.47)**	0.00000000		Zhang 2015	Highly suggestive (Insuff. data)
Total cancer, America	CC	Serum folate	3,548 (1,387)	MD	-0.24 (-0.54, 0.07)	0.12300190		Zhang 2015	Weak
Total cancer, Middle East	CC	Serum folate	1,728 (614)	MD	**-1.40 (-2.40, -0.41)**	0.00581938		Zhang 2015	Weak
**Dose-response relationship**									
Esophageal cancer	CC	Dietary folate	25,335 (1,209)	OR	**0.91 (0.88, 0.94)** for every 100 µg/d increase	0.00000002	0.02	Liu 2017	Highly suggestive
Head and neck cancer	CC	Dietary folate	7,281 (1,969)	OR	**0.96 (0.94, 0.98)** for every 100 µg/d increase	0.00012305		Fan 2017	Suggestive
Pancreatic cancer	PC	Dietary folate	418,612 (1,113)	OR	**0.94 (0.92, 0.97)** for every 100 µg/d increase	0.00000458		Liu (2017)	Suggestive

Table S5. List of meta-analyses using composite exposure measures of folate in association with cancer risks

Author (year)	Outcome	Study population/ subgroup	No. studies (design)	No. total (case)	Summary effect	I^2	P Egger
**Breast cancer – Diet/Total/Biomarkers**							
Zeng (2019)	Breast cancer	Premenopausal	7 (NR)	NR (NR)	RR=0.94 (0.88, 1.00)	15.1% (p=0.32)	
		Postmenopausal	14 (NR)	NR (NR)	RR=0.96 (0.94, 1.18)	40.3% (p=0.06)	
		ER+	6 (NR)	NR (NR)	RR=1.01 (0.86, 1.17)	67% (p=0.01)	
		ER-	7 (NR)	NR (NR)	RR=0.88 (0.78, 1.00)	6.5% (p=0.38)	
		PR+	6 (NR)	NR (NR)	RR=1.01 (0.92, 1.11)	59.7% (p=0.03)	
		PR-	6 (NR)	NR (NR)	RR=0.93 (0.83, 1.05)	0% (p=0.48)	
		ER+ PR+	6 (NR)	NR (NR)	RR=1.01 (0.91, 1.11)	23.9% (p=0.26)	
		ER- PR-	6 (NR)	NR (NR)	**RR=0.82 (0.68, 0.97)**	0% (p=0.81)	
		HER2+	4 (NR)	NR (NR)	RR=0.95 (0.69, 1.31)	0% (p=0.80)	
		HER2-	4 (NR)	NR (NR)	RR=0.97 (0.82, 1.15)	45.7% (p=0.14)	
		Low alcohol use	10 (NR)	NR (NR)	RR=0.97 (0.92, 1.03)	0% (p=0.69)	
		Medium alcohol use	10 (NR)	NR (NR)	**RR=0.91 (0.84, 0.98)**	12.1% (p=0.34)	
		High alcohol use	10 (NR)	NR (NR)	**RR=0.82 (0.72, 0.94)**	50.7% (p=0.03)	
**Colorectal cancer – Dietary/Total intake/ RBC folate**							
Moazzen (2018)	Colorectal cancer		20 (PC)	2,520,112 (NR)	**RR=0.71 (0.59, 0.86)**	51.6% (p=0.00)	0.30
			22 (CC)	12,042 (NR)	RR=0.77 (0.62, 0.95)	58.9% (p=0.00)	
			20 PC, 22 CC	2,532,154 (NR)	RR=1.05 (0.85, 1.30)		
**Esophageal adenocarcinoma (EAC) – Dietary/ Total intake/ Serum folate**							
Liu (2017)	EAC		4 (CC)	1,656 (633)	**OR=0.56 (0.37, 0.75)**	70.2% (p=0.00)	0.14
**Esophageal squamous cell carcinoma (ESCC) – Dietary/ Total intake/ Serum folate**							
Liu (2017)	ESCC		1 PC, 9 CC	34,690 (2,027)	**OR=0.55 (0.37, 0.73)**	80.5% (p<0.001)	0.15
**Esophageal cancer – Dietary/ Total intake/ Serum folate**							
Liu (2017)	Esophageal cancer		2 (PC)	523,133 (1,257)	OR=0.82 (0.57, 1.07)	51.4% (p=0.13)	0.47
			17 (CC)	31,866 (3,226)	**OR=0.50 (0.39, 0.61)**	68.3% (p<0.001)	0.08
		US	1 PC, 3 CC	495,030 (1,445)	**OR=0.57 (0.47, 0.67)**	12.3% (p=0.34)	0.57
		China	1 PC, 4 CC	31,685 (1,103)	**OR=0.60 (0.26, 0.94)**	91.7% (p<0.001)	0.17
**Esophageal cancer – Dietary intake/ Plasma/Serum folate**							
Ma (2018)	Esophageal cancer		1 PC, 1 NCC, 17 CC	8,951,438 (2,047)	**OR=0.69 (0.55, 0.86)**	77.2% (p=0.00)	NR
**Gastric cancer – Dietary/ Total intake/ Serum folate**							
Liu (2017)	Gastric cancer		5 (PC)	842,601 (1,998)	OR=0.97 (0.80, 1.13)	0% (p=0.62)	0.55
			16 (CC)	15,537 (4,883)	OR=0.70 (0.56, 0.83)	72.7% (p<0.001)	0.96
			5 PC, 16 CC	857,938 (6,881)	**OR=0.76 (0.65, 0.88)**	67.6% (p<0.001)	0.81
		US	1 PC, 4 CC	4,237 (2,031)	**OR=0.63 (0.54, 0.72)**	37% (p=0.13)	0.51
		Asia	2 PC, 5 CC	170,147 (2,115)	OR=0.86 (0.58, 1.15)	69% (p=0.00)	0.24

		Europe	2 PC, 3 CC	187,012 (1,693)	OR=0.89 (0.56, 1.22)	48.5% (p=0.08)	0.23
		Male	2 (NR)	NR (NR)	OR=0.60 (0.09, 1.11)	66.4% (p=0.09)	0.66
		Female	3 (NR)	NR (NR)	OR=0.86 (0.41, 1.31)	66.7% (p=0.05)	0.42
**Urothelial carcinoma – Dietary intake/ Serum folate**							
Gu (2022)	Urothelial carcinoma	Individuals aged 27-93 years	10 (PC)	475,116 (4,310)	RR=0.97 (0.87, 1.09)	0% (p=0.69)	
			9 (CC)	14,944 (7,140)	RR=0.56 (0.39, 0.79)	80.7% (p=0.00)	
			10 PC, 9 CC	490,060 (11,450)	**RR=0.78 (0.66, 0.93)**	NR	0.045
**Cervical cancer – Dietary intake/ Serum/ RBC folate**							
Myung (2011)	Cervical cancer	Women aged 18-84 years	9 (CC)	5,203 (1,757)	**OR=0.60 (0.41, 0.88)**	59.8%	NR
**Head and neck cancer – Dietary intake/ Serum folate**							
Fan (2017)	Head and neck cancer		9 (CC)	14,992 (4,090)	**OR=0.51 (0.39, 0.62)**	50.9%	0.28
**Head and neck squamous cell carcinoma – Dietary intake/ Serum folate**							
Fan (2017)	Head and neck squamous cell carcinoma		3 (CC)	3,147 (865)	**OR=0.69 (0.50, 0.87)**	37.7%	0.60
**Pancreatic cancer – Dietary/ Total intake/ Serum folate**							
Liu (2017)	Pancreatic cancer		7 PC, 5 CC	997,922 (3,670)	**OR=0.73 (0.56, 0.91)**	69% (p<0.001)	0.09
			7 (PC)	992,617 (2,017)	OR=0.80 (0.51, 1.09)	78.9% (p<0.001)	0.03
			5 (CC)	5,305 (1,653)	OR=0.59 (0.46, 0.72)	33.5% (p=0.20)	0.83
		US	2 PC, 2 CC	181,141 (1,232)	OR=0.89 (0.57, 1.21)	67% (p=0.03)	0.60
		Europe	4 PC, 1 CC	752,146 (1,619)	**OR=0.46 (0.33, 0.59)**	30.5% (p=0.22)	0.07
		Male	5 (NR)	NR (NR)	OR=0.86 (0.71, 1.00)	0% (p=0.74)	0.84
		Female	5 (NR)	NR (NR)	**OR=0.72 (0.56, 0.87)**	0% (p=0.58)	0.56

Table S6. Risk of bias assessment of the included syntheses using ROBIS

	Domain 1: Study eligibility criteria	Domain 2: Identification and selection of studies	Domain 3: Data collection and study appraisal	Domain 4: Synthesis and findings	Risk of Bias in the Review
Bailie L (2017)	⊕	⊕	⊕	⊕	⊕
Burr NE (2017)	⊕	⊖	⊗	⊗	⊗
Carroll KL (2022)	⊕	⊕	⊖	⊕	⊕
Chen P (2014)	⊕	⊖	⊕	⊕	⊕
Chen Y (2021)	⊕	⊗	⊕	⊕	⊗
Chiavarini M (2018)	⊕	⊕	⊕	⊕	⊕
Clasen JL (2020)	⊗	⊕	⊕	⊕	⊗
Colapinto CK (2016)	⊖	⊕	⊕	⊕	⊖
Collin SM (2010)	⊕	⊗	⊖	⊗	⊗
Cooper K (2010)	⊕	⊕	⊕	⊗	⊗
Crane TE (2014)	⊕	⊕	⊗	⊖	⊗
Davies AA (2006)	⊕	⊕	⊗	⊗	⊗
Dessypris N (2017)	⊕	⊕	⊕	⊗	⊗
Doganis D (2020)	⊖	⊕	⊕	⊕	⊗
Du L (2016)	⊕	⊕	⊕	⊕	⊕
Fan C (2017)	⊕	⊕	⊕	⊕	⊕
Fife J (2011)	⊕	⊕	⊗	⊕	⊗
Fu H (2023)	⊕	⊕	⊕	⊕	⊕
Garcia-Closas R (2005)	⊕	⊕	⊗	⊗	⊗
Gu Y (2022)	⊕	⊕	⊕	⊕	⊕
Heine-Broring RC (2015)	⊖	⊕	⊕	⊕	⊖
Hezaveh E (2021)	⊕	⊕	⊕	⊗	⊗
Ibrahim EM (2010)	⊗	⊗	⊗	⊗	⊗
Wan Ismail WR (2019)	⊗	⊗	⊕	⊕	⊗
Kennedy DA (2011)	⊖	⊕	⊕	⊕	⊖
Khodavandi A (2021)	⊕	⊕	⊕	⊖	⊖
Kubo A (2010)	⊗	⊕	⊗	⊗	⊗
Kumar S (2022)	⊕	⊕	⊕	⊕	⊕
Larsson SC (2006)	⊕	⊗	⊗	⊕	⊗
Lei J (2022)	⊕	⊕	⊕	⊗	⊗
Li B (2015)	⊕	⊗	⊕	⊕	⊗
Li C (2013)	⊗	⊕	⊗	⊕	⊗
Lin H (2013)	⊕	⊕	⊗	⊕	⊗
Liu M (2014)	⊕	⊗	⊕	⊕	⊗

Liu W (2017)	⊕	⊕	⊗	⊕	⊗
Liu Y (2015)	⊕	⊗	⊕	⊕	⊗
Ma JL (2018)	⊕	⊗	⊕	⊗	⊗
Mao B (2015)	⊕	⊗	⊕	⊗	⊗
Misotti AM (2013)	⊖	⊕	⊗	⊗	⊗
Moazzen S (2018)	⊕	⊕	⊗	⊕	⊗
Myung SK (2011)	⊗	⊕	⊗	⊕	⊗
Ni Y (2017)	⊗	⊕	⊖	⊕	⊗
Nucci D (2021)	⊕	⊕	⊕	⊕	⊕
Qiang Y (2018)	⊕	⊕	⊕	⊕	⊕
Qin T (2015)	⊕	⊖	⊖	⊕	⊖
Ren X (2020)	⊕	⊕	⊕	⊕	⊕
Ryan-Harshman M (2007)	⊗	⊗	⊗	⊗	⊗
Sanjoaquin M (2005)	⊗	⊗	⊗	⊗	⊗
Shiao SPK (2018)	⊕	⊕	⊗	⊗	⊗
Sun LP (2020)	⊕	⊕	⊕	⊕	⊕
Sun M (2018)	⊖	⊕	⊗	⊕	⊗
Tio M (2014) - Gastrointestinal	⊕	⊕	⊗	⊕	⊗
Tio M (2014) – Prostate	⊕	⊕	⊗	⊗	⊗
Tio M (2014) - Breast	⊕	⊕	⊗	⊕	⊕
van Dijk M (2016)	⊕	⊕	⊕	⊕	⊕
Vollset SE (2013)	⊗	⊗	⊗	⊗	⊗
Wang K (2021)	⊕	⊕	⊕	⊕	⊕
Wang R (2014)	⊕	⊕	⊕	⊕	⊕
Wien TN (2012)	⊕	⊕	⊕	⊕	⊕
Yang J (2018)	⊕	⊕	⊕	⊕	⊕
Zeegers MP (2004)	⊗	⊖	⊗	⊗	⊗
Zeng J (2019)	⊖	⊗	⊗	⊗	⊗
Zhang D (2015)	⊕	⊕	⊖	⊖	⊖
Zhao Y (2017)	⊕	⊕	⊕	⊕	⊕
Zhao Z (2021)	⊕	⊕	⊕	⊕	⊕
Zhou X (2016)	⊕	⊕	⊕	⊕	⊕
Zhou YH (2011)	⊕	⊖	⊕	⊕	⊕
High	11	14	24	20	38
Low	49	48	38	44	23
Unclear	7	5	5	3	6

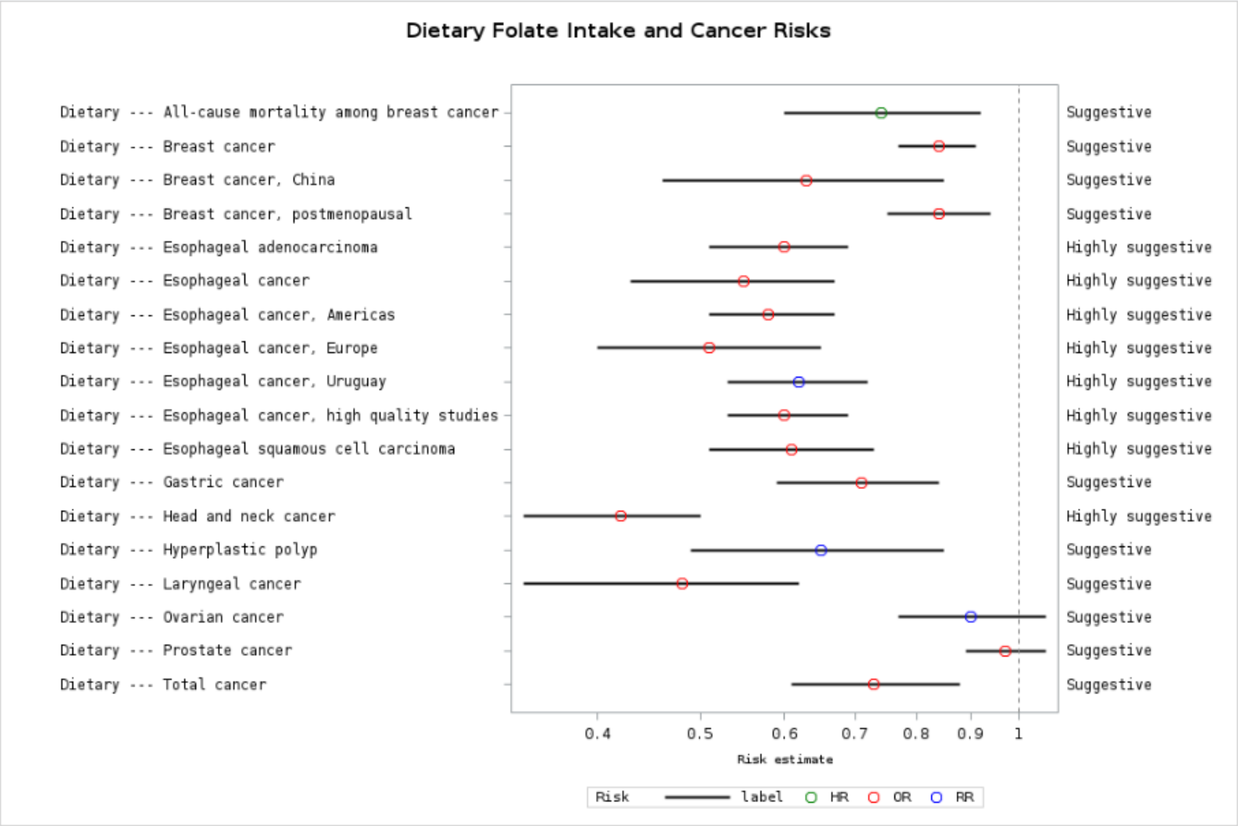

Figure S1. Unique associations between dietary folate intake and cancer risks, assessed to be highly suggestive or suggestive

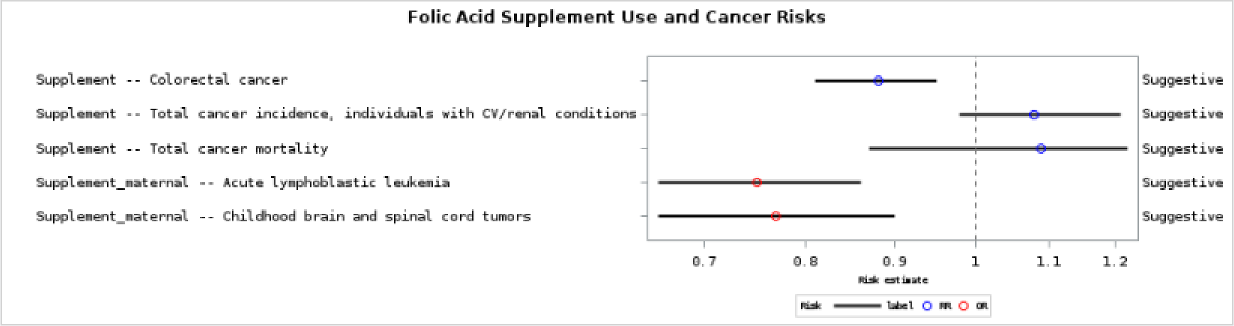

Figure S2. Unique associations between folic acid supplement use and cancer risks, assessed to be highly suggestive or suggestive

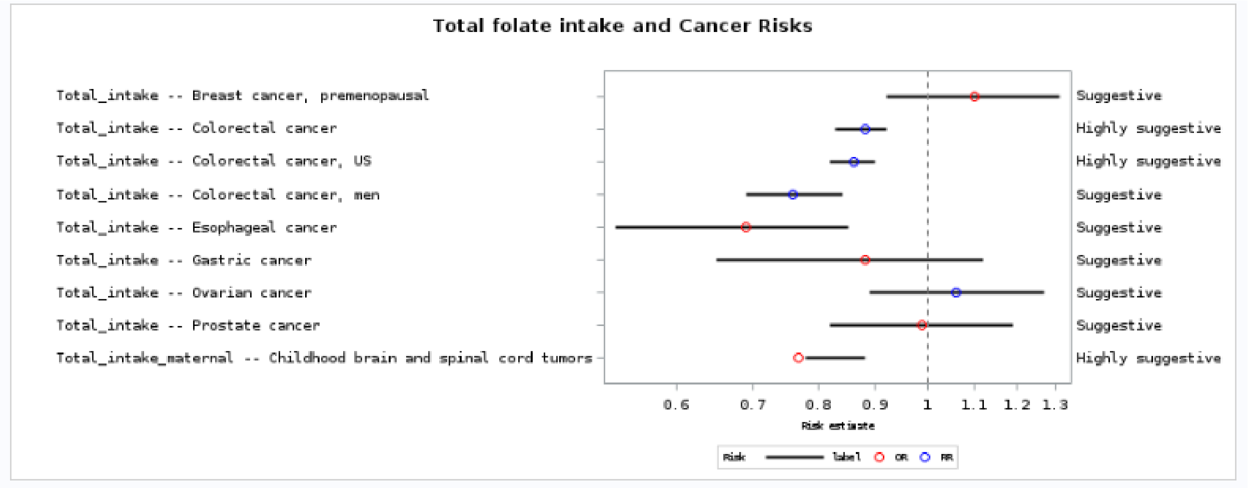

Figure S3. Unique associations between total folate intake and cancer risks, assessed to be highly suggestive or suggestive

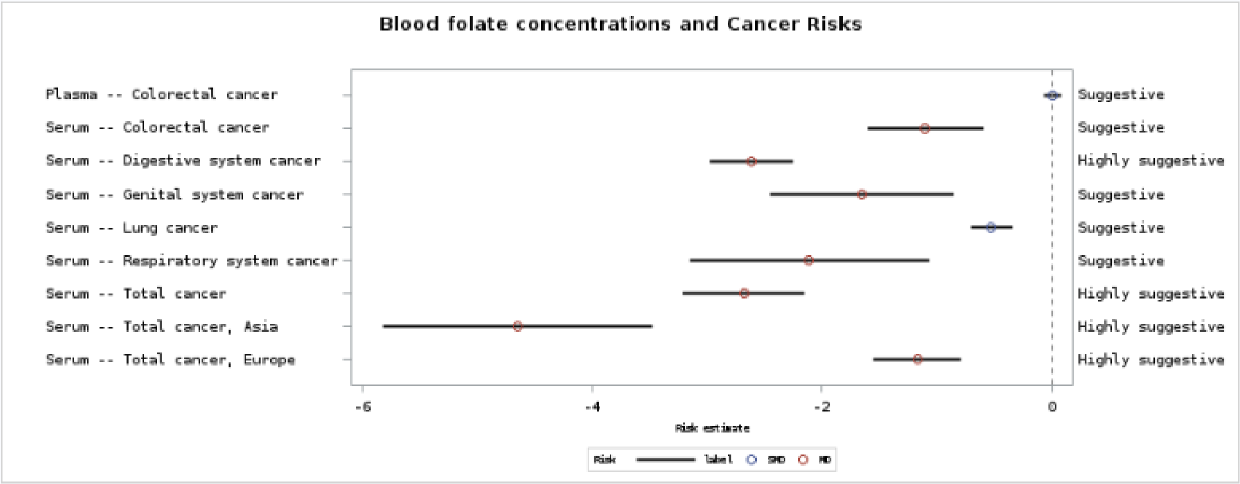

Figure S4. Unique associations between blood folate concentrations and cancer risks, assessed to be highly suggestive or suggestive
